# Identification and Characterization of Mortaparib^Plus^—A Novel Triazole Derivative That Targets Mortalin-p53 Interaction and Inhibits Cancer-Cell Proliferation by Wild-Type p53-Dependent and -Independent Mechanisms

**DOI:** 10.3390/cancers13040835

**Published:** 2021-02-17

**Authors:** Anissa Nofita Sari, Ahmed Elwakeel, Jaspreet Kaur Dhanjal, Vipul Kumar, Durai Sundar, Sunil C. Kaul, Renu Wadhwa

**Affiliations:** 1 AIST-INDIA DAILAB, National Institute of Advanced Industrial Science & Technology (AIST), Central 5-41, Tsukuba 305-8565, Japan; sari-anissa@aist.go.jp (A.N.S.); ahmed.elwakeel@aist.go.jp (A.E.); jaspreet@iiitd.ac.in (J.K.D.); 2 School of Integrative & Global Majors (SIGMA), University of Tsukuba, Tsukuba 305-8577, Japan; 3 DAILAB, Department of Biochemical Engineering & Biotechnology, Indian Institute of Technology (IIT) Delhi, Hauz Khas, New Delhi 110-016, India; vipul.kumar@dbeb.iitd.ac.in (V.K.); sundar@dbeb.iitd.ac.in (D.S.)

**Keywords:** cancer, mortalin-p53 interaction, novel small-molecule triazole derivative, PARP1, inhibition, potential anticancer drug

## Abstract

**Simple Summary:**

Functional inactivation of tumour suppressor protein p53 is frequently found in a large variety of cancers. One of the mechanisms by which p53 is inactivated is through its interaction with mortalin protein that inhibits its translocation, and hence the function, in the nucleus. Abrogation of mortalin-p53 interaction has been suggested as a target for cancer therapy. We report here a novel multimodal small molecule, called Mortaparib^Plus^, that causes growth arrest or apoptosis of cancer cells by abrogating mortalin-p53 interaction yielding reactivation of p53 function. It also causes upregulation of tumour suppressor protein p73, and inactivation of PARP1 and CARF proteins accounting for its multimodal anticancer activity.

**Abstract:**

p53 has an essential role in suppressing the carcinogenesis process by inducing cell cycle arrest/apoptosis/senescence. Mortalin/GRP75 is a member of the Hsp70 protein family that binds to p53 causing its sequestration in the cell cytoplasm. Hence, p53 cannot translocate to the nucleus to execute its canonical tumour suppression function as a transcription factor. Abrogation of mortalin-p53 interaction and subsequent reactivation of p53’s tumour suppression function has been anticipated as a possible approach in developing a novel cancer therapeutic drug candidate. A chemical library was screened in a high-content screening system to identify potential mortalin-p53 interaction disruptors. By four rounds of visual assays for mortalin and p53, we identified a novel synthetic small-molecule triazole derivative (4-[(1E)-2-(2-phenylindol-3-yl)-1-azavinyl]-1,2,4-triazole, henceforth named Mortaparib^Plus^). Its activities were validated using multiple bioinformatics and experimental approaches in colorectal cancer cells possessing either wild-type (HCT116) or mutant (DLD-1) p53. Bioinformatics and computational analyses predicted the ability of Mortaparib^Plus^ to competitively prevent the interaction of mortalin with p53 as it interacted with the p53 binding site of mortalin. Immunoprecipitation analyses demonstrated the abrogation of mortalin-p53 complex formation in Mortaparib^Plus^-treated cells that showed growth arrest and apoptosis mediated by activation of p21^WAF1^, or BAX and PUMA signalling, respectively. Furthermore, we demonstrate that Mortaparib^Plus^-induced cytotoxicity to cancer cells is mediated by multiple mechanisms that included the inhibition of PARP1, up-regulation of p73, and also the down-regulation of mortalin and CARF proteins that play critical roles in carcinogenesis. Mortaparib^Plus^ is a novel multimodal candidate anticancer drug that warrants further experimental and clinical attention.

## 1. Introduction

Colorectal cancer (CRC) is the third most common cause of cancer death worldwide [1], of which the incidence is rapidly increasing [2]. The essential treatment for CRC is either surgical resection or radiotherapy or both together and in combination with chemotherapy pre-and/or post-operation [3]. There are several FDA-approved chemotherapeutic drugs that are used as a specific regimen in CRC treatment. These include oxaliplatin, 5-fluorouracil, capecitabin, and/or a combination of irinotecan with targeted agents such as anti-epidermal growth-factor receptor (EGFR) drugs (cetuximab or panitumumab) or anti-angiogenetic compounds (bevacizumab or aflibercept) [4,5]. These also involve other drugs that target DNA damage response (DDR), cell-cycle checkpoint pathways, poly(ADP-Ribose) polymerase 1 (PARP1), vascular endothelial growth factor (VEGF), and heat-shock protein 70 family (HSP70) [6,7].

Mortalin (also known as mthsp70/HSPA9/GRP75) is a member of the heat-shock protein 70 (hsp70) family of proteins. It is enriched in a variety of cancers and has been considered as a candidate anticancer drug target [8,9,10,11,12,13]. Overexpression of mortalin was shown to cooperate with telomerase and extend the in vitro lifespan of normal human fibroblasts [14]. It has been shown to play a pioneering role in control of cell proliferation, malignant transformation, carcinogenesis, migration, metastasis, angiogenesis and cancer-cell stemness, protein folding, chaperoning, intracellular trafficking, and apoptosis [12,13,15,16,17,18,19,20]. Mortalin-knockdown using specific ribozymes and shRNA was shown to cause growth arrest and apoptosis in cancer cells [21,22,23,24]. Overexpression of mortalin was detected in CRC, hepatocellular carcinoma (HCC), ovarian carcinoma, and several other cancers [9,10,20,24,25]. It has been correlated with poor survival of CRC and HCC patients [9,25]. Mortalin knockdown was shown to reverse EMT, stemness, and drug resistance in cancer cells [13,26,27].

p53 plays key roles in control of cell-cycle checkpoints and DNA repair and is inactivated in a large majority of cancers [28,29]. Reactivation of wild type p53 activities in human transformed cells has been shown to trigger growth arrest/apoptosis in vitro and in vivo [30,31,32,33,34,35]. Mortalin protein has been known to interact with and inactivate tumour suppressor p53 in cancer cells in vitro and in vivo [16,17,22,23,36,37,38,39]. Mortalin-p53 interaction has been implicated in the deregulation of apoptosis contributing to carcinogenesis [15,22,23,40,41]. In line with this, abrogation of mortalin-p53 complex by synthetic and natural molecules, including MKT-077, Mortaparib, withaferin A, withanone, cucurbitacin B, and fucoxanthin were shown to cause growth arrest and/or apoptosis of cancer cells in in vitro, and cause tumour growth suppression in in vivo assays [32,42,43,44,45].

Inactivation of DNA-damage-response (DDR) pathways is a common characteristic of both familial and sporadic colorectal cancers [46]. Genotoxic chemotherapeutic drugs used as current medical regiments for CRC patients, including oxaliplatin, irinotecan, and 5-fluororacil (5-FU), are direct or indirect inducers of DNA damage that is recognized by specific repair pathways. Interfering with those repairing pathways is deemed beneficial for CRC patients. Hence, inhibition of proteins involved in DNA-damage-repair pathways, for instance topoisomerase I (Top1), thymidylate synthase, and PARP1, has been proposed as a novel therapeutic strategy for colorectal carcinogenesis [47,48,49]. Although PARP1 inhibitors are used to treat malignancies (such as cervical and ovarian cancer) that often possess BRCA1/2 mutations and an impaired homologous-recombination (HR) mechanism [49,50], the use of PAPR1 inhibitors in the CRC treatment has not yet been well established and is the subject of current investigations. In the present study, we identified a novel triazole analogue (4-[(1E)-2-(2-phenylindol-3-yl)-1-azavinyl]-1,2,4-triazole) as a molecule that inhibited mortalin-p53 interactions and caused activation of p53 function. Similar to the earlier-reported Mortaparib, it caused inhibition of PARP1. Furthermore, it also caused growth arrest/apoptosis of cancer cells by p53-independent (involving p73, CARF and p21^WAF1/CIP1^) mechanisms and was hence named Mortaparib^Plus^. We provide in silico and experimental evidence that Mortaparib^Plus^ induces growth arrest and apoptosis in CRC by p53-dependent and -independent pathways.

## 2. Results

### 2.1. Identification of Mortaparib^Plus^ as a Novel Anticancer Small Molecule That Disrupts Mortalin-p53 Interaction

**Figure 1:**
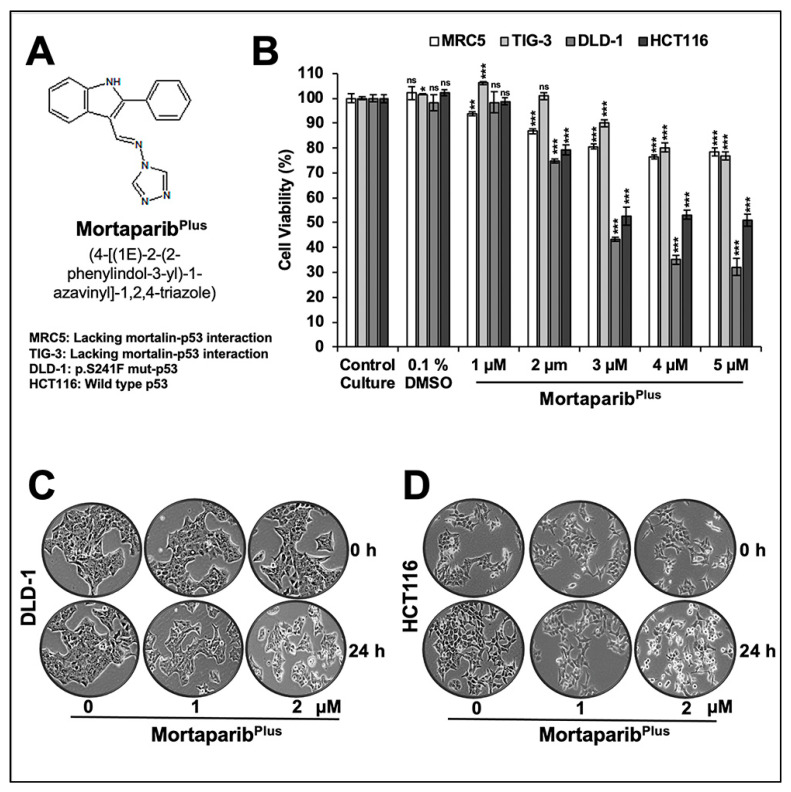
Mortaparib^Plus^ is cytotoxic to human colorectal cancer cells and relatively nontoxic to normal lung fibroblasts. Chemical structure of Mortaparib^Plus^ (**A**). Short-term (24 h) cell-viability assays for control and Mortaparib^Plus^-treated cells (MRC5 and TIG-3 lung fibroblasts; DLD-1 and HCT116 colorectal cancer cells) showed a dose-dependent cytotoxicity in colorectal cancer cells with a milder effect on normal lung fibroblasts (MRC5 and TIG-3; the nontumorigenic cell models) (**B**). Phase contrast micrographs of control and Mortaparib^Plus^-treated DLD-1 and HCT116 cancer cells showed a marked stress phenotype (condensation and blebbing morphologies) in Mortaparib^Plus^-treated cells as compared to the untreated control (**C**,**D**). The quantified cell-viability data represents mean ± SD obtained from three independent biological replicates; *p*-values were calculated using unpaired Student’s *t*-test. * < 0.05, ** < 0.01, and *** < 0.001 represent significant, very significant, and very very significant, respectively.

**Figure 2:**
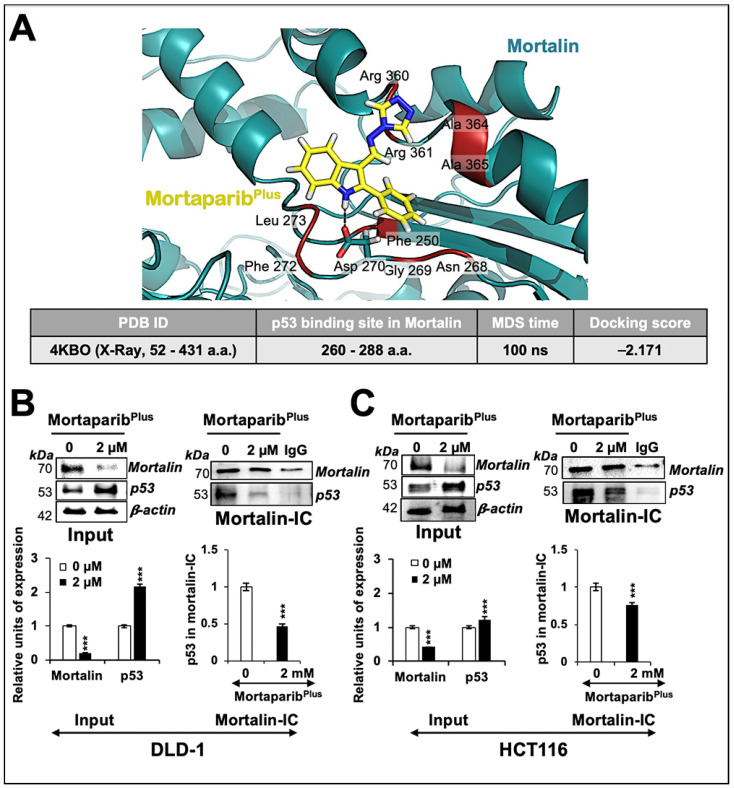
Mortaparib^Plus^ abrogated the interaction of mortalin and p53 in silico and in vitro. Molecular-docking analyses showed an interaction of Mortaparib^Plus^ with the interface of mortalin involved in p53 binding (**A**). Co-immunoprecipitation analyses using a rabbit polyclonal anti-mortalin antibody (**B**,**C**) showed a decrease in p53 fractions (wild type or mutant) only in mortalin complexes immunoprecipitated from Mortaparib^Plus^-treated (24 h) colorectal cancer cells. A decline in mortalin levels in the input non-immunoprecipitated samples from the same cell lysates was observed. The quantified data represents mean ± SD obtained from three independent biological replicates; *p*-values were calculated using unpaired Student’s *t*-test. *** < 0.001 represent significant, very significant and very very significant, respectively.

We had earlier screened a library of 12,000 compounds for their potential to disrupt p53-mortalin interaction. In four rounds of screenings, based on a shift in mortalin staining from perinuclear (typically characteristic of cancer cells) to pan-cytoplasmic (typically characteristic of normal cells) and nuclear translocation of p53 in treated cells, six compounds were selected. A small molecule, Mortaparib, that caused the inhibition of both mortalin-p53 interaction and PARP1 activity was reported earlier [45]. In the present study, we report a novel triazole derivative (4-[(1E)-2-(2-phenylindol-3-yl)-1-azavinyl]-1,2,4-triazole (Figure 1A) named Mortaparib^Plus^ that was selected as a potential mortalin-p53 disruptor in the same screening using human osteosarcoma (U2OS) and Luminal-A breast-cancer (MCF-7) cells, both possessing wild-type p53 protein. In order to validate the anticancer activity and mechanism of action of Mortaparib^Plus^, we recruited colorectal HCT116 (with wild-type p53 (p53^WT^)) and DLD-1 (with the mutant p53 (p53^S241F^)) cell lines. Firstly, as shown in Figure 1B, cell-viability assays revealed a dose-dependent decrease in cell viability in both HCT116 (p53^WT^) and DLD-1 (p53^S241F^). Of note, normal human lung fibroblasts (TIG-3 and MRC5) did not show an equivalent cytotoxicity when treated with Mortaparib^Plus^. Based on these data, we selected 1 μM (lower dose) and 2 μM (higher dose) of Mortaparib^Plus^ for further analyses. Microscopic observations of control and both the treated HCT116 and DLD-1 cancer-cell lines showed a distinctive morphology suggestive of growth arrest with the low dose and apoptosis with the high dose of Mortaparib^Plus^ (Figure 1C,D). We next investigated the structural homology of Mortaparib^Plus^ to the known chemicals in PubChem, ChemEMBL, and DrugBank databases, and to the drugs currently being used for treatment of colorectal cancer. Mortaparib^Plus^ turned out to be a novel compound and as shown in Supplementary Figure S1, structural alignment using PyMol revealed that Mortaparib^Plus^ has no similarity with currently known clinical and preclinical molecules for colorectal-cancer therapy. Based on these data, we predicted that Mortaparib^Plus^ may provide a novel drug involving new/different mechanism(s) of action. In premises of the identification of Mortaparib^Plus^ as a potential novel abrogator of mortalin-p53 interactions and a selective cytotoxic candidate to cancer cells, we performed computational analyses to confirm such potentiality in silico. Molecular docking analyses revealed that Mortaparib^Plus^ could bind to mortalin but not the wild-type p53 (Figure 2A). It showed interaction at the interface of mortalin involved in p53 binding with a docking score of −2.171 kcal/mol. The docked complex was then subjected to a molecular-dynamics simulation of 100 ns in the explicit water-model system. Mortaparib^Plus^ was found to be quite stable as observed in the structures equally spanned over the simulation trajectory (Supplementary Figure S2A). A representative structure was computed by averaging the structural coordinates of conformations extracted from the most stable duration of the simulation trajectory. Asp270 of mortalin was found to be involved in H-bond formation with Mortaparib^Plus^. The other residues contributing to this stable bonding via hydrophobic interactions included Phe250, Asn268, Gly269, Asp270, Phe272, Leu273, Arg360, Arg361, Ala364, and Ala365. Furthermore, we examined the interaction of Mortaparib^Plus^ with p53^S241F^ and two other mutants (p53^R248W^ and p53^R273H^) that are commonly found in CRC. Similar to the wild-type p53, these mutants showed only a very weak interaction with Mortaparib^Plus^; the binding site was away from the mutated residues and close to the mortalin-binding site (Supplementary Figure S3A,B). Also, the binding of Mortaparib^Plus^ to the mortalin-binding region of p53 was not affected by any of these mutations (Supplementary Figure S3A). In order to validate these in silico results, indirect co-immunoprecipitation analyses were performed using a rabbit polyclonal antibody to immunoprecipitate equal amounts of mortalin complex from the control and treated cell lysates. As shown in Figure 2B,C, mortalin complexes from control and treated cells showed a decrease in p53 in the latter, regardless of its status as mutant (DLD-1 (p53^S241F^)) or wild type (HCT116 (p53^WT^)). Of note, Mortaparib^Plus^-treated cells showed a decrease in mortalin level and an increase in p53 level as shown in Figure 2 (non-immunoprecipitated input samples from the same cell lysates used for immunoprecipitation).

### 2.2. Mortaparib^Plus^ Caused Downregulation of Mortalin

**Figure 3:**
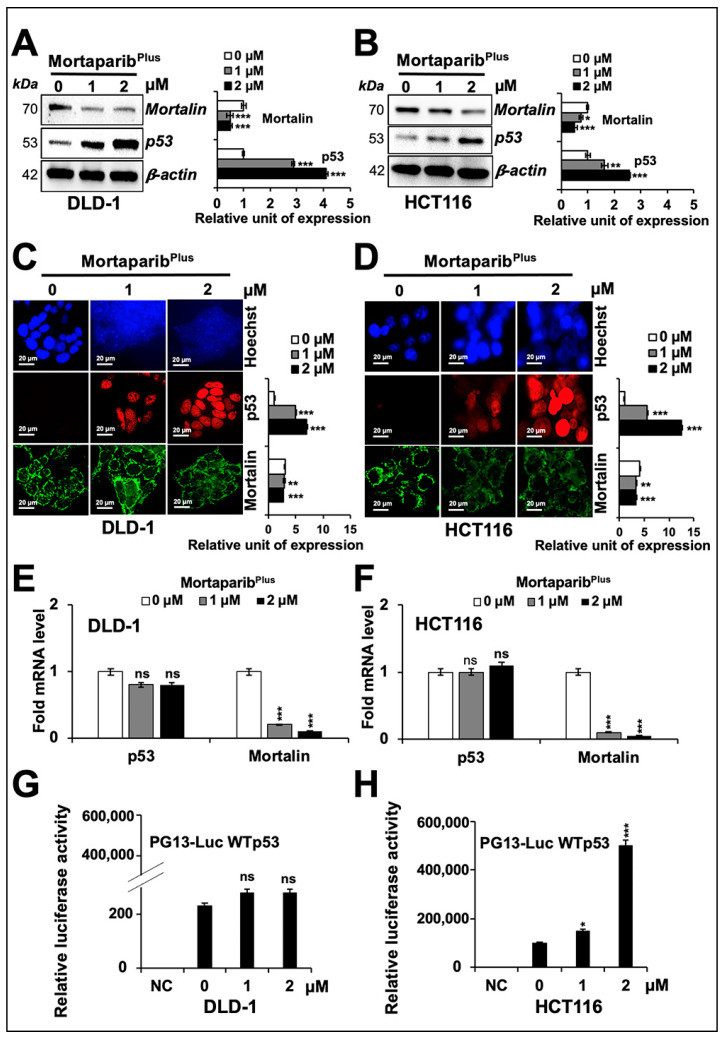
Mortaparib^Plus^ down-regulated mortalin levels in colorectal cancer cells. Cells treated with Mortaprib^Plus^ for 24 h were analysed for the expression levels of mortalin and p53 with respect to the untreated control cells by Western blotting (**A**,**B**), immunocytochemistry (**C**,**D**), and real-time quantitative polymerase chain reaction (RT-qPCR) (**E**,**F**). A decrease in mortalin and increase in p53 levels were confirmed in all the methods adopted. Wild-type p53-specific luciferase reporter assays (PG13-luc) did not show any significant increase in p53-driven luciferase activity in Mortaparib^Plus^-treated DLD1 (p53^S241F^) cells (**G**). HCT116 (p53^WT^) cells 24 h-treated with Mortaparib^Plus^ showed a dramatic increase in luciferase activity using the same reporter assay systems (**H**). The quantified data represents mean ± SD obtained from three independent biological replicates; *p*-values were calculated using unpaired Student’s *t*-test. * < 0.05, ** < 0.01, and *** < 0.001 represent significant, very significant and very very significant, respectively. Scale bar in C and D equals 20 μm.

Several experiments on expression analyses of mortalin by Western blotting in control and Mortaparib^Plus^-treated cells consistently revealed a dose-dependent decrease in mortalin, also confirmed by immunocytochemistry (Figure 3A–D). Additionally, p53 showed a dose-dependent increase (Figure 3A,B). p53 immunostaining was negligible in the control HCT116 (p53^WT^) and DLD-1 (p53^S241F^) cells, and Mortaparib^Plus^-treated cells showed clear p53 enrichment in the nuclei of both the cell lines (Figure 3C,D). Of note, as shown in Figure 3C,D, the shift in mortalin staining pattern from perinuclear in control to pan-cytoplasmic in treated cells was also confirmed in both HCT116 (p53^WT^) and DLD-1 (p53^S241F^) cells. Of note, mRNA-expression analyses showed a significant decrease in mortalin mRNA (Figure 3E,F), but p53 mRNA did not show an increase. These data suggested that (i) Mortaparib^Plus^ caused transcriptional down-regulation of mortalin, (ii) the accumulation in p53 protein was not mediated at a transcriptional level, and (iii) the nuclear translocation of p53 protein may be enhanced as an outcome of decrease in mortalin expression in Mortaparib^Plus^-treated cells. We also performed the wild-type p53-specific luciferase reporter (PG13-luc) assay to examine the transcriptional activation function of p53. As shown in Figure 3H, the wild-type p53-dependent luciferase reporter activity showed a dose-dependent remarkable increase in Mortaparib^Plus^-treated HCT116 (p53^WT^) cells only. Such an activation was not attained in the Mortaparib^Plus^-treated DLD-1 cells possessing the S241F point mutant p53 (Figure 3G). Taken together, these data endorsed the abrogation of mortalin-p53 interaction, re-translocation of p53 to the nucleus, and activation of transcriptional functions of the wild-type p53 in Mortaparib^Plus^ -treated cells.

### 2.3. Mortaparib^Plus^ Caused Apoptosis and Cell-Cycle Arrest in DLD-1 (p53^S241F^) and HCT116 (p53^WT^) Cells through p53-Dependent and -Independent Manners

**Figure 4:**
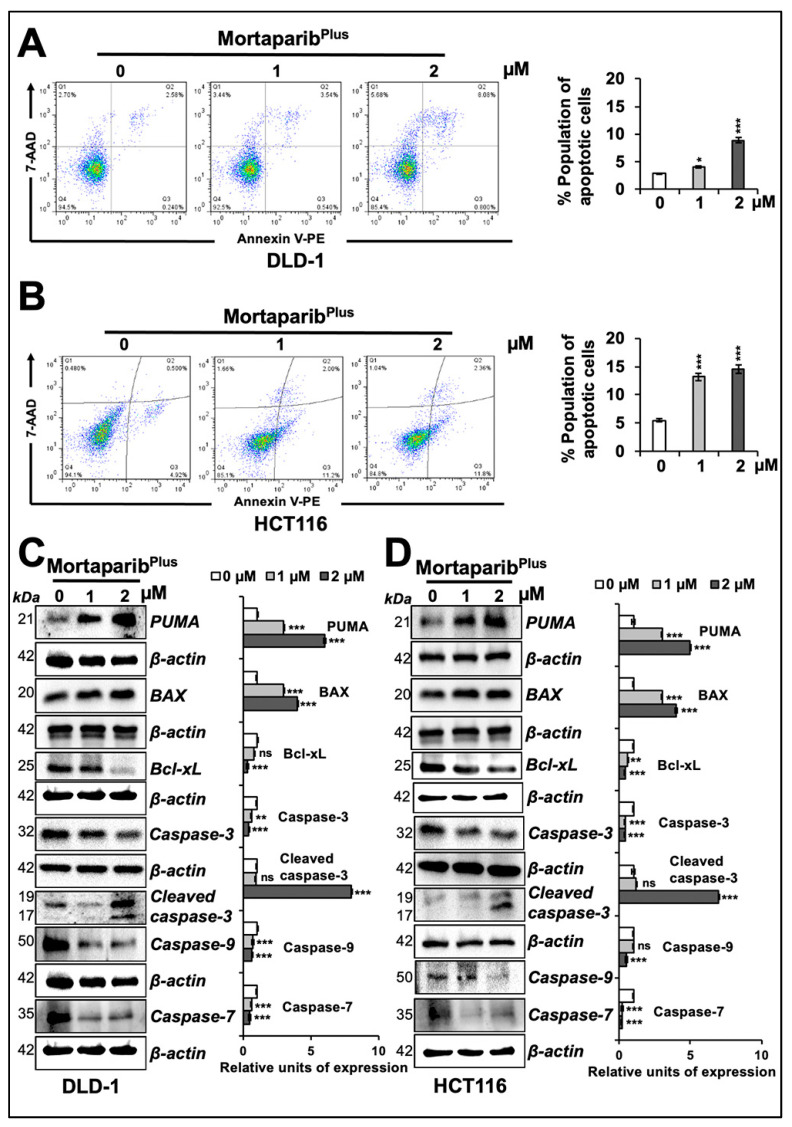
Mortaparib^Plus^ induced apoptosis in colorectal cancer cells. Flow cytometric analyses for control and 24 h Mortaprib^Plus^-treated cells showed an increase in the percentage of apoptotic cell populations in a dose-dependent manner (**A**,**B**). Western-blot analyses of control and 24 h Mortaparib^Plus^-treated colorectal cancer cells showed different changes in the levels of key proteins favouring the initiation of apoptosis as a tumour suppression fate (**C**,**D**). Note that the loading control (β-actin) for PUMA and Caspase-9, and Bcl-xL and Caspase-7 in panel **C** are the same. Similarly, β-actin for Bcl-xL and Caspase-7 & -9 in panel **D** is the same. β-actin for Caspase-3 in panel **D** is also the same as in Figure 3B. The quantified data represents mean ± SD obtained from at least three independent biological replicates; *p*-values were calculated using unpaired Student’s *t*-test. * < 0.05, ** < 0.01, and *** < 0.001 represent significant, very significant and very very significant, respectively.

**Figure 5:**
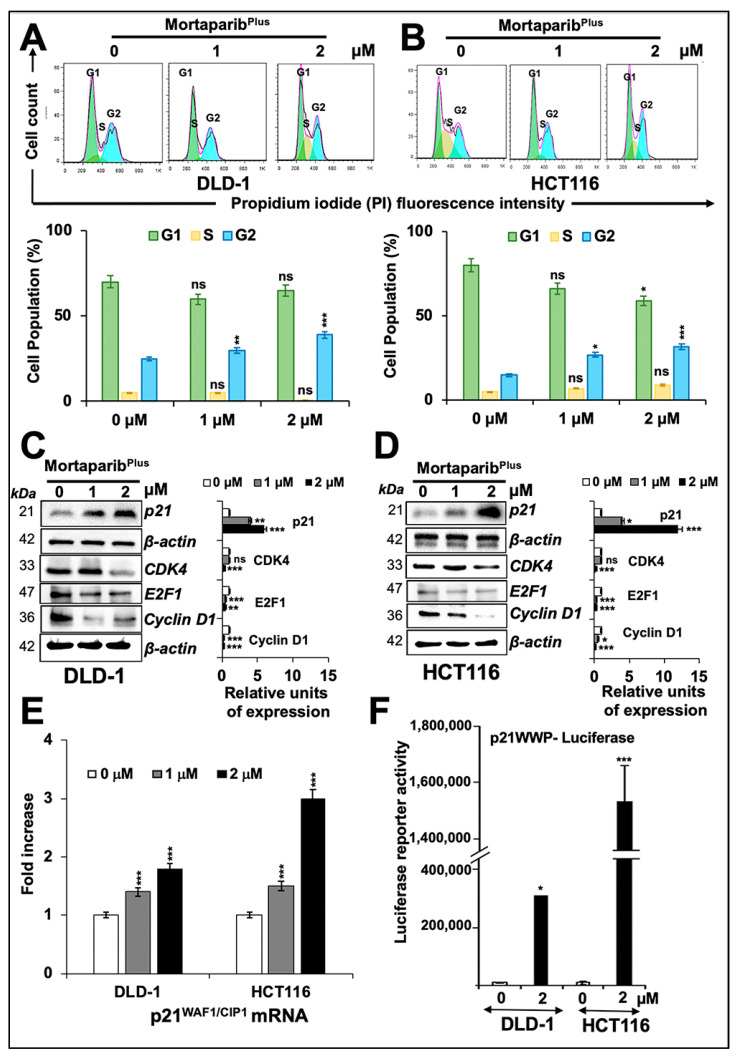
Mortaparib^Plus^-treated colorectal cancer cells underwent G_2_/M cell-cycle arrest. Flow cytometric analyses for control and Mortaprib^Plus^-treated cells showed an increase in the percentage of cell populations in the G_2_ phase of cell cycle (**A**,**B**). Western-blot analyses of control and 24 h Mortaparib^Plus^-treated cells showed an increase in p21^WAF1^ protein levels and a decline in CDK4, E2F1 and Cyclin D1 proteins (**C**,**D**). Note that the loading control (β-actin) for panel **C** and **D** (CDK4, E2F1 and Cyclin D1) is the same as the one used for Caspase-7 shown in Figure 4C,D, respectively. RT-qPCR analyses showed a dose-dependent increase in the *p21^WAF1^* gene expression in 24 h Mortaparib^Plus^-treated cells (**E**). Luciferase reporter assays using pWWP-Luc containing p21^WAF1^ promoter showed a strong increase in the luciferase activity in the 24 h Mortaparib^Plus^-treated HCT116 (p53^WT^) cells and a moderate increase in Mortaparib^Plus^-treated DLD1 (p53^S241F^) cells after the same incubation time (**F**). The quantified data represents mean ± SD obtained from three independent biological replicates; *p*-values were calculated using unpaired Student’s *t*-test. * < 0.05, ** < 0.01, and *** < 0.001 represent significant, very significant and very very significant, respectively.

In order to get further insights to the mechanism of action of Mortaparib^Plus^, we next examined control and Mortaparib^Plus^-treated cells for apoptosis/growth arrest. As shown in Figure 4A,B, both DLD-1 (p53^S241F^) and HCT116 (p53^WT^) cells showed a dose-dependent increase in the percentage of apoptotic cells in response to Mortaparib^Plus^ treatment. Consistent with this phenotype, the treated cells showed a dose-dependent increase in pro-apoptotic proteins (PUMA, BAX and cleaved Caspase-3); and a decrease in anti-apoptotic protein Bcl-xL with a decline in the levels of caspase enzymes (Caspase-3, Caspase-9, and Caspase-7) both in DLD-1 (p53^S241F^) and HCT116 (p53^WT^) cells (Figure 4C,D). The molecular response in HCT116 cells could be attributed to translocation and activation of wild-type p53. DLD-1 cells that possesses mutant p53 were anticipated to undergo apoptosis through decrease in mortalin and p53-independent pathways. We next performed cell-cycle analysis in control and Mortaparib^Plus^-treated cells. As shown in Figure 5A,B, with the Mortaparib^Plus^ treatment, both DLD-1 (p53^S241F^) and HCT116 (p53^WT^) cells showed a dose-dependent increase in the percentage of cell population in G_2_ phase. Expression analyses of key regulatory proteins controlling cell-cycle progression revealed an increase in p21^WAF1/CIP1^ and decline in CDK4, E2F1 and Cyclin D1 proteins in treated DLD-1 (p53^S241F^) and HCT116 (p53^WT^) cells (Figure 5C,D). Having a wild-type p53 status, the G_2_/M cell cycle arrest in Mortaparib^Plus^-treated HCT116 cells could be attributed to an activation of the p53-p21^WAF1/CIP1^ axis. However, DLD-1 cells, in contrast to HCT116, possessed a mutant p53, and hence, the increase in p21^WAF1/CIP1^, a wild-type p53 effector, remained to be addressed.

Earlier, we have shown that withaferin-A and withanone could selectively interact with the Y220C mutant form of p53 and restore its wild-type functional activity [44]. However, as previously mentioned (Figure 3G), Mortaparib^Plus^-treated DLD-1 cells (p53^S241F^) did not show significant increase in wild-type p53-dependent luciferase reporter activity. Additionally, as described above, Mortaparib^Plus^ was unable to bind strongly/stably to the mortalin binding site of p53. The interaction of Mortaparib^Plus^ with p53^S241F^ was comparable to its interaction with p53^WT^ because the site of point mutation in p53^S241F^ lies far from the mortalin binding site. Hence, in DLD-1 (p53^S241F^) cells, we hypothesized that Mortaparib^Plus^ may cause activation of p21^WAF1/CIP1^ in a p53-independent manner. Accordingly, to validate this hypothesis, we first confirmed that *p21^WAF1/CIP1^* mRNA expression was dose-dependently increased in Mortaparib^Plus^-treated DLD-1 (p53^S241F^) cells (Figure 5E). Furthermore, we performed reporter assays using pWWP-luc containing *p21^WAF1/CIP1^* promoter. As shown in Figure 5F, and as expected, a strong increase in pWWP-luc reporter activity was recorded in Mortaparib^Plus^-treated HCT116 (p53^WT^) cells. Interestingly, DLD-1 (p53^S241F^) cells also showed moderate and significant increase in pWWP-luc reporter activity upon Mortaparib^Plus^ treatment, suggesting that p21^WAF1/CIP1^ is activated in a p53-independent modality.

### 2.4. Mortaparib^Plus^ Activated p21^WAF1/CIP1^ in a p53-Independent Manner

**Figure 6:**
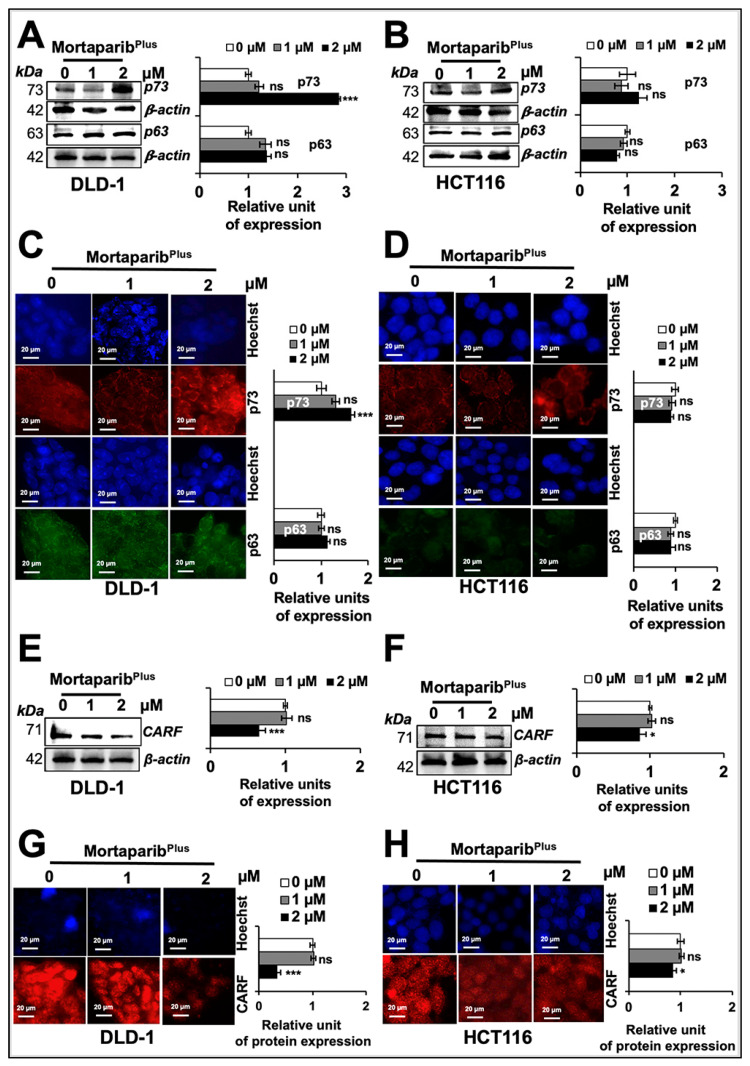
Mortaparib^Plus^ activates p21^WAF1^ in a p53-independent manner. Control and 24 h Mortaprib^Plus^-treated cells were analysed for the levels of p73 and p63 proteins by Western blotting (**A**,**B**) and immuno-cytochemistry (**C**,**D**). There were no significant changes in p63 protein levels between control and Mortaparib^Plus^-treated DLD1 and HCT116 cells. p73 protein levels showed an increase in Mortaparib^Plus^-treated DLD1 cells; but not in Mortaparib^Plus^-treated HCT116 cells. p63 and CARF in DLD-1 cell lysates were detected in the same blot (therefore, the same β-actin band was used for loading control). The collaborator of p14^ARF^ (CARF) protein levels in control and 24 h Mortaparib^Plus^-treated cells were analysed by Western blotting (**E**,**F**) and immuno-cytochemistry (**G**,**H**). CARF protein was down-regulated in Mortaparib^Plus^-treated DLD1 and HCT116 cells. The quantified data represents mean ± SD obtained from three independent biological replicates; *p*-values were calculated using unpaired Student’s *t*-test. * < 0.05 and *** < 0.001 represent significant, very significant and very very significant, respectively. Scale bar in C and D equals 20 μm.

In order to resolve Mortaparib^Plus^-induced p53-independent p21^WAF1^-activation in DLD1 (p53^S241F^) cells, we next investigated the expression of p63 and p73, two other members of the p53 family of transcription factors. Both p63 and p73 share a high degree of sequence similarity with p53, particularly in the DNA-binding domain, allowing them to transactivate, at least in part, p53-target genes responsible for cell-cycle arrest and apoptosis [51,52]. As shown in Figure 6A,B, using an antibody that could detect the full-length TAp63-α, an isoform known to bind to DNA through p53 responsive elements, control and Mortaparib^Plus^-treated cells showed no change in p63 expression. These findings were also supported by the immuno-cytochemistry data (Figure 6C,D). Next, to examine the expression levels of p73 transcription factor in control and Mortaparib^Plus^-treated cells, we recruited an antibody that was raised using a synthetic peptide fragment within the amino acid sequence 50 to 150 of the p73 protein (a fragment between the DNA-binding domain and the transactivation domain). Interestingly, there was an increase in expression of p73 in DLD-1 (p53^S241F^) cells only; HCT116 (p53^WT^) cells did not show any significant changes (Figure 6B). The results were also supported by the immuno-cytochemistry data (Figure 6C,D). Taken together, the data suggested the Mortaparib^Plus^-induced p21^WAF1/CIP1^ activation and growth arrest in DLD-1 (p53^S241F^) cells might be through an activation of p73 member of the p53 family of proteins.

We recently reported that the CARF (collaborator of p14^ARF^) protein could cause the transcriptional repression of p21^WAF1^ in cancer cells in a p53-independent way [53]. In light of these findings, we next examined whether or not CARF expression level could be affected in Mortaparib^Plus^-treated cells. As shown in Figure 6E,F, Mortaparib^Plus^-treated cells showed a down-regulation of CARF. Of note, in several independent experiments on expression analyses by Western blotting and immuno-cytochemistry, DLD-1 (p53^S241F^) cells showed stronger down-regulation of CARF than in HCT116 (p53^WT^) cells (Figure 6E,H).

### 2.5. Mortaparib^Plus^ Impaired DNA-Damage-Repair Signalling through Multiple Modalities

**Figure 7:**
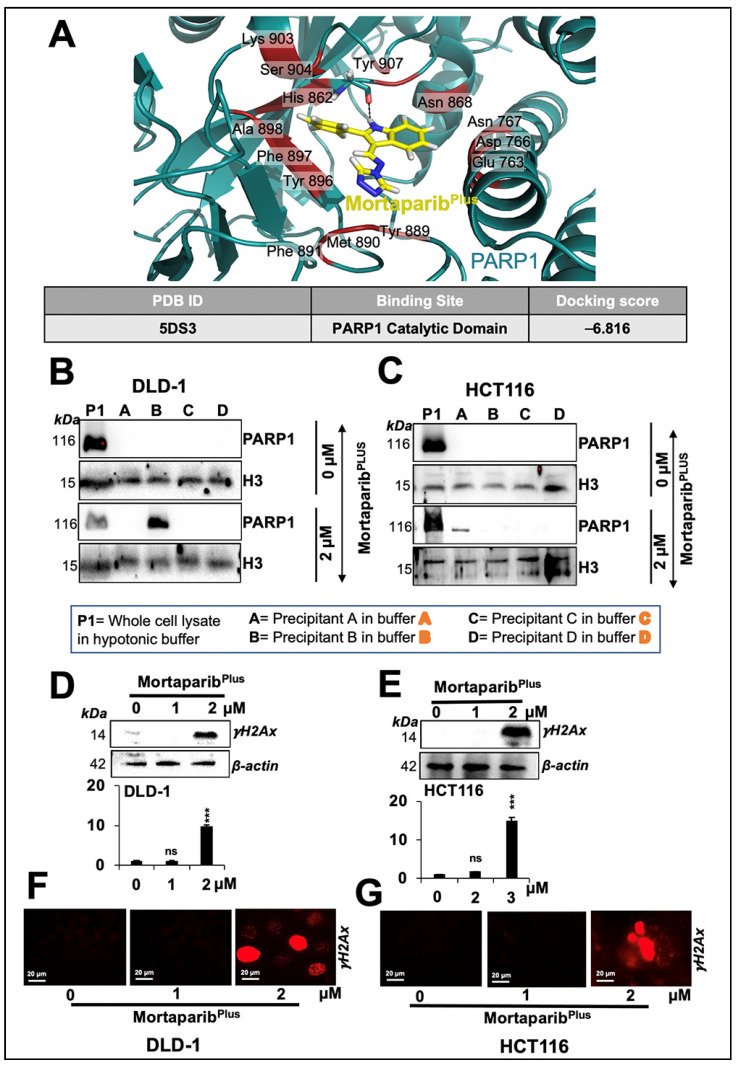
Mortaparib^Plus^ caused inhibition of PARP1 catalytic activity resulting in an impairment of the DNA-damage-repair signalling. Molecular-docking analyses showed an interaction of Mortaparib^Plus^ with PARP1 catalytic domain (**A**). Western-blotting analyses of PARP1-DNA complexes of 24 h Mortaparib^Plus^-treated colorectal cancer cells showed trapping of PARP1 in DNA (**B**,**C**). The levels of the phosphorylated H2A histone variant X (γH2AX) in control and 24 h Mortaparib^Plus^-treated cells were analysed by Western blotting (**D**,**E**) and immuno-cytochemistry (**F**,**G**) that showed an increase in the number of γH2AX foci in the treated cells. Scale bar in immuno-cytochemistry equals 20 μm. The quantified data represents mean ± SD obtained from three independent biological replicates; *p*-values were calculated using unpaired Student’s *t*-test *** < 0.001 represent significant, very significant and very very significant, respectively.

**Figure 8:**
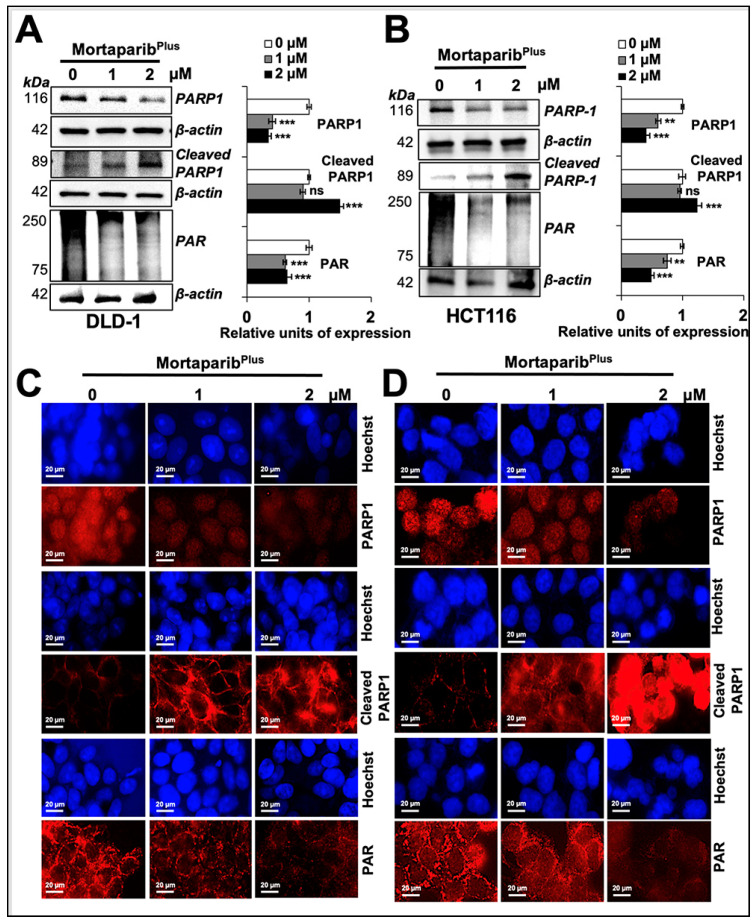
Mortaparib^Plus^-induced apoptosis in colorectal cancer cells resulted in the cleavage of PARP1 and the further decline in its catalytic activity. PARP1, cleaved PARP1, and PAR levels were analysed in control and 24 h Mortaparib^Plus^-treated cells by Western blotting (**A**,**B**) and immuno-cytochemistry (**C**,**D**). As compared to the untreated control, Mortaparib^Plus^-treated cells showed a decrease in full-length PARP1 with an increase in its 89-kDa cleaved fragment leading to the decumulation of PAR polymer. Scale bar in immuno-cytochemistry equals 20 μm. The quantitation of immunocytochemistry results are provided in Supplementary Figure S4B. The data represent mean ±SD obtained from three independent biological replicates; *p*-values were calculated using unpaired Student’s *t*-test. ** < 0.01 and *** < 0.001 represent significant, very significant and very very significant, respectively.

CRC is a complex and heterogenous disease with a well-characterized cascade of molecular oncogenic events. One of these events is the anomalies in the DNA-mismatch repair machinery. Accordingly, CRC could be classified into two major subtypes: microsatellite instable or microsatellite stable. Previously, it had been proposed that microsatellite-instable CRC cells are vulnerable to PARP1 inhibition. This could be attributed to the MSI-induced mutagenesis in genes responsible for the homologous recombination repair of the double-strand DNA damage governing a “BRCAness-like” phenotype [48,54]. Additionally, not only a microsatellite-instable type of CRC cells, HCT116 (p53^WT^) and DLD-1 (p53^S241F^) cells, have mis-sense point mutations in ATM and ATR, respectively, according to the Catalogue of Somatic Mutations in Cancer (COSMIC) databases. Hence, we anticipated that Mortaparib^Plus^ may result in an inactivation of DNA-damage-repair signalling in both wild-type and mutant p53 harbouring colorectal cancer cells. Accordingly, we next studied the inhibitory potential of Mortaparib^Plus^ against the PARP1 protein using computational methods. The known inhibitors of PARP1 have been shown to interact with its catalytic domain [55,56,57]. The residues of PARP1 responsible for this catalytic activity mainly include His862, Tyr896, and Glu988 [58]. Firstly, we docked Mortaparib^Plus^ against PARP1 by generating a grid around these functional residues. The binding affinity was found to be good as evident from the docking score of -6.816 kcal/mol. The molecule showed hydrogen-bonded interaction with one of the critical residues, His862. Moreover, the collective effect of nonbonded interactions of Mortaparib^Plus^ with many other residues of PARP1 (Figure 7A) further strengthened this binding. To further account for the dynamic stability of this binding, we subjected the docked complex to 100 ns molecular dynamics simulations. Mortaparib^Plus^ did not deviate much from its docked orientation and stably stayed inside the catalytic pocket of the protein throughout the simulation trajectory (Supplementary Figure S2B), suggesting Mortaparib^Plus^ may directly inhibit catalytic activity of PARP1. In light of these computational predictions, we performed a PARP1-DNA trapping assay in control and treated cells. As shown in Figure 7B,C, PARP1 was trapped into the DNA in the Mortaparib^Plus^-treated cells. Inhibition of DNA repair in treated cells was also supported by the accumulation of the phosphorylated H2A histone variant X (γH2AX) (Figure 7D,E) that also showed the DNA-damage foci by immuno-cytochemistry (Figure 7F,G). We next investigated PARP1 and PAR levels in control and Mortaparib^Plus^-treated cells. As shown in Figure 8A,B, decrease in full-length PARP1 and increase in the 89-kDa cleaved fragment of PARP1 were observed both in DLD-1 (p53^S241F^) and HCT116 (p53^WT^) cells. Furthermore, using an anti-PAR polymer antibody that could detect both PAR polymer and auto-PARylated PARP1 [59,60], we observed a significant decline in the PAR accumulation levels in treated cells. These data were also supported by immuno-cytochemistry results (Figure 8C,D and Supplementary Figure S4). Taken together, the data demonstrated that Mortaparib^Plus^ possesses the ability to inactivate the DNA-damage-repair signalling and causes growth arrest/apoptosis of cancer cells by multiple p53-dependent and -independent modalities.

## 3. Discussion

Annually, CRC account for approximately 10% of all the diagnosed cancers and cancer-related mortalities around the globe. Furthermore, it is the second-most-commonly diagnosed cancer in women and third most in men [61]. Despite the recent endeavours to improve the early detection and treatment methods, CRC remains yet an unmet medical need. Interventional strategies for CRC range from endoscopic treatments, surgery, radiotherapy, and systemic therapies as adjuvant and neoadjuvant modalities. However, surgery is still considered the mainstay with adjuvant and/or neoadjuvant chemotherapy to prevent the relapse and target micro-metastases. At a molecular level, there are two major precursor anomalous pathways deriving colorectal cancer: (i) the chromosomal instability pathway (the canonical adenoma–carcinoma pathway) that leads to approximately 70–90% of colorectal cancer cases and (ii) the serrated neoplasia pathway that leads to almost 10–20% of colorectal cancers [61]. Ample scientific evidence suggests that these pathways represent a sequential acquisition of genetic and epigenetic aberrations that govern the initiation, promotion, and progression of CRCs. For instance, chromosomal instability phenotypes are typically initiated by a mutation in the adenomatous polyposis gene (*APC*), followed by a mutation-derived overactivation of the KRAS proto-oncogene GTPase (KRAS), and/or the function loss of p53 [62]. Accordingly, drug-discovery regimes have recently been focusing on targeting of multiple CRC-implicated oncoproteins and/or signalling pathways.

Mortalin (GRP75/HSPA9/mthsp70) is a highly conserved member of the heat-shock protein 70 family of chaperones with multimodal oncogenic functions [19,22,63,64,65] and a candidate anticancer drug target [10,66,67,68,69]. Overexpression of mortalin in colorectal adenocarcinomas has been correlated with poor patient survival rates [9]. Furthermore, in an independent study, improved short-term survival rates were found in early or advanced CRC stages associated with lower mortalin levels [70]. Those reports suggested the involvement of the mortalin’s multimodal oncogenic roles in CRC progression. Then, in an attempt to elucidate one of the major oncogenic roles of mortalin in CRC, Gestl and Böttger [71] had previously concluded that mortalin inactivates the wild-type p53 protein through cytoplasmic sequestration in four CRC cell lines (HCT116, HT-29, LS123, and LoVo). Accordingly, in the present study, we recruited HCT116 (with wild-type p53 (p53^WT^)) and DLD-1 (with the mutant p53 (p53^S241F^)) cells to validate the anticancer activity and mechanism of action of Mortaparib^Plus^ (a potent mortalin-p53 interaction disruptor selected after a library screening step as previously mentioned). Mortaparib^Plus^ showed a potent cytotoxicity against both CRC cell lines regardless of their p53 status. Interestingly, as a normal nontumorigenic cell model, Mortaparib^Plus^-treated MRC5 and TIG-3 cells were relatively resistant. Cancer-cell-selective mortalin and p53 interaction may be attributed to such differential cytotoxicity of Mortaparib^Plus^ [23]. At a molecular level, and based on in vitro and in silico analyses, we concluded that Mortaparib^Plus^ could successfully abrogate the interaction between mortalin and p53 in both HCT116 [p53^WT^] and DLD-1 [p53^S241F^] cells. Additionally, we found that Mortaparib^Plus^ caused down-regulation of mortalin both at the transcript and protein levels. Such a dual effect (abrogation of mortalin’s interaction with p53 and down-regulation of its mRNA) of Mortaparib^Plus^ was sufficient enough for the p53 (the wild type and the S241F point mutant) to translocate to and accumulate in the nucleus. Then, owing to its functional status, p53 protein’s signalling was successfully activated in the Mortaparib^Plus^-treated HCT116 (p53^WT^) cells. The data was supported by the activation of transcriptional activation function of p53 as evidenced by the increase in p53-driven proteins (p21^WAF1^, PUMA, and BAX) involved in apoptosis and cell-cycle arrest. On the contrary, DLD-1 cells possessing the S241F point mutant did not show reactivation of transcriptional activation function of p53 but possessed an increased level of expression of *p21^WAF1^*. The latter was attributed to the activation of the p73 member of the p53 family of transcription factors and down-regulation of the collaborator of p14^ARF^ (CARF) protein that has been previously shown to repress p21^WAF1/CIP1^ in a p53-independent manner. Furthermore, mortalin has previously been reported to have several oncogenic interaction partners [19,63,72]. For instance, it interacts with the human telomerase complex and the heterogenous nuclear ribonucleoprotein-K (hnRNP-K) in the nucleus causing their stabilization and activation, hence, contributing to malignancy and metastasis [19]. Additionally, by the physical interaction with the mitogen-activated protein-kinase (MAP2K or MEK) proteins, mortalin was found to be as a negative regulatory focal point of the aberrant Raf/MEK/ERK pathway-mediated growth-inhibitory signalling [72]. In light of these molecular findings, together with its up-regulated oncogenic expression in human colorectal cancer tissues, the Mortaparib^Plus^-mediated down-regulation of mortalin expression in colorectal cancer cells possessing a mutant p53 (as in the case of DLD-1 cells (p53^S241F^)) could provide a successful molecular therapeutic strategy.

In the so-called “BRCAness”, BRCA1/2 mutations are the biomarkers to predict cancer patients that could benefit from PARP1 inhibitors as a therapeutic strategy. However, it was previously reported that not all patients carrying mutant BRCA1/2 could benefit from PARP1 inhibitors. Hence, a further broadening of the patient spectrum was proposed to include those with a mutation or an inactivation in other genes involved in the homologous recombination repair pathway in the so-called BRCAness-like phenotype. Hence, to harness the BRCAness-like phenotype of our in vitro CRC model, and with the aim to valorise our newly identified Mortaparib^Plus^ as a multimodal anticolorectal-cancer candidate molecule, we next investigated its effect on PARP1 signalling. In vitro and in silico analyses revealed that Mortaparib^Plus^ inhibited the PARP1 catalytic activity without interfering with its DNA-binding capabilities in both HCT116 (p53^WT^) and DLD-1 (p53^S241F^) cells as follows: (i) the decrease in the poly ADP ribose (PAR) polymer and/or the auto-PARylated PARP1 protein levels, and (ii) the trapping of PARP1 into the DNA after the Mortaparib^Plus^ treatment. Such inhibition led to an accumulation of the DNA damage as measured by an increase in γH2AX foci. Furthermore, owing to the initiation of apoptosis in the Mortaparib^Plus^-treated cells, the full length PARP1 decreased with a subsequent increase in the 89-kDa cleaved fragment of PARP1 (known for its reduced catalytic activity [73]) suggesting an intensification of the decline in the DNA-repair capability. Taken together, Mortaparib^Plus^ could serve as a multimodal anticancer candidate drug for CRC treatment and hence, warrant further attention in laboratory and clinic for deciphering its molecular mechanisms of action and efficacy, respectively.

## 4. Materials and Methods

### 4.1. Cell Culture and Reagents

Human colorectal cancer (DLD-1 and HCT116), and normal (MRC5 and TIG-3) cells were procured from the Japanese Collection of Research Bioresources (JCRB) Cell Bank, Osaka, Japan. All the cells were cultured in Dulbecco’s Modified Eagle’s Medium (DMEM) (Fujifilm WAKO Pure Chemical Corporation, Osaka, Japan) and supplemented with 5–10% fetal bovine serum (Thermo Fisher Scientific, Japan) and 1% penicillin/streptomycin (Invitrogen, Carlsbad, CA, USA) in a humidified incubator (37 °C and 5% CO_2_).

### 4.2. Library Screening

The screening of 12,000 compounds (synthetic and natural) had been previously described [45].

### 4.3. Drug Preparation and Treatment

Stock solution (5 mM) of 4-((1E)-2-(2-phenylindol-3-yl)-1-azavinyl)-1,2,4-triazole (Mortaparib^plus^) (NAMIKI SHOJI Co., Ltd. (Shinjuku, Japan)) was prepared in Dimethyl Sulfoxide (DMSO) (WAKO, Osaka, Japan) and then added to the complete cell-culture medium to obtain different working concentrations as indicated. Cells were treated with Mortaparib^plus^ after being grown at 60–70% confluency. For microscopic observations, cells were cultured in six-well plates, and routinely examined in both live and fixed states (as described below in immuno-cytochemistry section).

### 4.4. Cytotoxicity/Growth-Inhibition Assay

Cells (5 × 10^3^)/well were seeded in a 96-well plate (TPP^®^, Trasadingen, Switzerland) and allowed to adhere overnight by incubation at 37 °C in a humidified CO_2_ incubator. The cells were treated with the highest (*v/v*) concentration of DMSO that was used as a vehicle and different concentrations of Mortaparib^plus^ (1–5 μM) for 24 h. Cytotoxicity of Mortaparib^plus^ was evaluated by the quantitative colorimetric assay using the MTT (3-(4,5-dimethylthiazol-2-yl)-2, 5-diphenyltetrazolium bromide (Sigma-Aldrich, Japan) as follows: Cell-culture medium was replaced with fresh medium and MTT (0.5 mg/mL) solution and incubated for 4 h. Then, the medium-plus-MTT solution was replaced with DMSO (100 μL), and plates were shaken for 5 min to dissolute the produced formazan crystals. The absorbance was measured at 570 nm on a microplate reader (TECAN Infinite 200 PRO, Mannedorf, Switzerland).

### 4.5. PG13-luc and pWWP-luc Luciferase-Reporter Assays

DLD-1 and HCT116 colorectal cancer cells (18 × 10^4^/well) were seeded in 6-well plate and allowed to adhere at 37 °C in a humidified CO_2_ incubator overnight. Cells were transfected with the wild-type p53 responsive luciferase-reporter plasmid (PG13-luc) and the firefly luciferase-reporter gene driven by p21^WAF1/CIP1^ (pWWP-luc) (both plasmids were a kind gift from Professor Bert Vogelstein). Cells were transfected with the plasmids using X-tremeGENE HP DNA transfection reagent (Roche, Basel, Switzerland) in a volume ratio of 1:1 plasmid DNA to transfection reagent in serum free DMEM medium for 24 h. The transfected cells were treated with control and Mortaparib^plus^ for 24 h. The cells were then washed with PBS, collected and lysed using the passive lysis buffer (PLB) (Promega, WI, USA). The activity of luciferase was detected using the Luciferase^®^ Reporter Assay System (Promega, Madison, WI, USA) by a luminescence microplate reader (Tecan infinite M200^®^ Pro) (Mannedorf, Switzerland).

### 4.6. Immunoblotting

Control and Mortaparib^plus^-treated cells were harvested after 24 h, lysed using RIPA Lysis Buffer (Thermo Fisher Scientific, Waltham, MA, USA) containing complete protease inhibitor cocktail (Roche Applied Science, Mannheim, Germany) and shaken in a cold room for 30 min. Lysates were centrifuged at 15,000 rpm for 15 min, and the supernatants were used for the Western-blotting analysis as follows: The protein concentrations of whole-cell lysates were measured by the Pierce BCA Protein Assay Kit (Thermo Fisher Scientific, Waltham, MA, USA). The cell lysates (10–40 μg) were separated in 6–15% SDS-polyacrylamide gel electrophoresis (SDS-PAGE) and transferred to a polyvinyl dene difluoride (PVDF) membrane (Millipore, Billerica, MA, USA) using a semidry transfer blotter (ATTO Corporation, Tokyo, Japan). Membranes were blocked with 3% fraction-V bovine serum albumin at room temperature for 2 h. Blocked membranes were probed with the following target protein-specific primary antibodies: p53 (DO-1), PARP1/2 (H-250), pro-caspase 3 (H-277), Bcl-xL (8362), BAX (H20X), CDK4 (C-22), E2F1 (KH-95), Cyclin D1 (DSC-6), Caspase 9 (H-83), Caspase 7 (C-12), and Histone 3 (FL-136) (Santa Cruz Biotechnology, Paso Robles, CA, USA); p63 (4892S), PUMA (D30C10), p21^WAF1/CIP1^ (12D1), cleaved-PARP (D214), cleaved-caspase 3 (Asp175) and phospho-histone H2A.X (Ser139) (20E3) (Cell Signaling Technology, Danvers, MA, USA); and PAR (ab14459) and p73 (ab40658), (Abcam, Cambridge, UK) at 4 °C overnight. Anti-mortalin (376) and -CARF antibodies were generated in our laboratory. The blots were incubated with the following secondary antibodies conjugated to horseradish peroxidase: anti-rabbit IgG, anti-goat IgG, and anti-mouse IgG (Santa Cruz Biotechnology, CA, USA) and developed by enhanced chemiluminescence (ECL) (GE Healthcare, Buckinghamshire, UK). Anti-β-actin antibody (Abcam, Cambridge, UK) was used as an internal loading control. ImageJ (National Institutes of Health, Bethesda, MD, USA) software was used to quantitate the protein signals.

### 4.7. Immunocytochemistry

Cells (1 × 10^3^–3 × 10^3^/well) were plated on 18-mm glass coverslips placed in 12-well culture dishes (TPP, Trasadingen, Switzerland). After incubation for 24 h, the cells were treated with Mortaparib^plus^ for 24 h, then washed twice with PBS and fixed in methanol: acetone (1:1) at 4 °C for 5–10 min. After the removal of the fixation solution, cells were washed thrice with PBS then permeabilized by PBS with 0.1% Triton X-100 (PBST) for 10 min. The cells on coverslips were blocked with 2% bovine serum albumin containing primary antibodies (1–5 µg/mL) overnight (the detailed information of antibodies used are mentioned in the western-blot-analysis section). Immunostaining was visualized by secondary antibody staining with either Texas RED (Amersham Biosciences, Buckinghamshire, UK) or fluorescein isothiocyanate (FITC), and Alexa-488 or Alexa-594 conjugated antibodies (Molecular Probes, Eugene, OR, USA). After two hours of secondary antibody probing, cells were washed thrice with PBST (each 10 min) followed by counterstaining with Hoechst 33,342 (Invitrogen, Molecular Probes, Eugene, OR, USA) for nuclear staining. The cells were then washed for 10 min each, first with PBST, then PBS, and finally with double-distilled H_2_O. The coverslips were mounted on glass slides and examined under Zeiss Axiovert 200 M microscope. Analyses of cell images were done by AxioVision 4.6 software (Carl Zeiss, Tokyo, Japan). ImageJ (NIH, Bethesda, MD, USA) software was used to quantify the fluorescence signals.

### 4.8. Immunoprecipitation

Control and higher dose Mortaparib^Plus^-treated cells were harvested after 24 h and lysed using the non-ionic NP-40 buffer. The protein concentrations of whole-cell lysates were measured by BCA assay (Thermo Fisher Scientific, Rockford, IL). Cell lysates containing 300–500 μg of total protein were incubated overnight with control IgG (2729) (Cell Signaling Technology) and an antimortalin antibody (raised in our laboratory) in slow rotation at 4 °C. Lysates were centrifuged at 2500 rpm for 3 min, followed by the addition of A/G PLUS-Agarose beads (Santa Cruz Biotech Inc. sc-2003) and incubation of the mixture in slow rotation at 4 °C for 4 h. Immunoprecipitants were collected by cold (4 °C) centrifugation at 2500 rpm for 5 min in a microcentrifuge. Supernatants were removed from the beads and discarded. Pellets containing beads and protein(s) of interest were washed 3–4 times with NP-40 buffer, then centrifugated at 4 °C (2500 rpm) for 5 min. Pellets were mixed with SDS buffer and boiled at 96 °C for 10 min. The immunoprecipitants were resolved on SDS-PAGE, transferred to a PVDF membrane, and then detected with specific antibody for Western blotting.

### 4.9. Apoptosis Assay

DLD-1 and HCT116 cells were seeded in 6-well plates. After 24 h, control and Mortaparib^Plus^-treated cells were collected by centrifugation at 3000 rpm at 4 °C for 5 min. Floating cells were also pooled by centrifugation. The cell pellets were resuspended with 100 µL fresh media and stained with Guava Nexin Reagent (EMD Millipore Corporation, Berlington, MA, USA). Apoptosis analyses were done by Guava PCA-96 System (Luminex Corporation, Austin TX, USA). Apoptotic cells were detected and quantified by FlowJo software (Version 7.6, Flow Jo, LLC, Ashland, OR, USA).

### 4.10. Cell-Cycle Analysis

DLD-1 and HCT116 cells were seeded in the 6-well plates. After 24 h, control and Mortaparib^Plus^-treated cells were harvested, cold (4 °C)-centrifuged at 2000 rpm for 5 min, washed with cold PBS, fixed with 70% ethanol on slow vortex, and finally kept at −20 °C for up to 72 h. The fixed cells were cold (4 °C)-centrifuged at 3000 rpm for 10 min followed by two cycles of cold PBS washing. The cells were then stained with Guava^®^ Cell Cycle Reagent (4500-0220) (Luminex Corporation, Austin, TX, USA) in the dark for 30 min. RNA was degraded by treatment with RNase-A (1 mg/mL at 37 °C for 30 min). Cell-cycle progression was analysed using Guava^®^ PCA-96 System (Luminex Corporation, Austin, TX USA). FlowJo software (Version 7.6, Flow Jo, LLC, Ashland, OR, USA) was used to analyse the obtained flow cytometry data.

### 4.11. Trapping Assay

Control and Mortaparib^Plus^-treated cells were collected by centrifugation (2500 rpm) at 4 °C for 3 min. As previously reported [45], supernatants were removed, and pellets were mixed with TRAP assay buffers (constituted for different stringency as shown in Table 1).

Cell pellets were incubated with the indicated buffers, vortexed for 10 min followed by cold (4 °C) centrifugation at 16,000 rpm for 10 min. The supernatants were collected and labelled as P1, and thereafter, the pellets were resuspended with buffer A, followed by centrifugation at 16,000 rpm and 4 °C for 10 min. This step was repeated within the sequence of A–D buffers. Supernatants from each centrifugation step were labelled as A, B, C, and D and subjected to western-blotting analysis using anti-PARP1/2 and antihistone H3 antibodies.

### 4.12. Alignment Analysis

Structure alignment and visualization of Mortaparib^Plus^ with known anticolorectal cancer drugs was performed using PyMol (Version 2.2.3, Schrödinger, Inc.). The structure of Mortaparib^Plus^ was prepared using MarvinSketch chemical drawing tool (ChemAxon, Budapest, Hungary) and the structures of known drugs were collected from PubChem database (https://pubchem.ncbi.nlm.nih.gov/) accessed on 12 January 2019.

### 4.13. RNA Extraction

Total RNA from control (DMSO) and Mortaparib^Plus^-treated cells were collected by RNeasy mini kit (Qiagen, Stanford Valencia, CA, USA) following the manufacturer’s protocol.

### 4.14. Quantitative Real-Time Polymerase Chain Reaction

Equal amounts of RNA from samples were used for reverse transcription following the protocol from QuantiTect reverse transcription kit (Qiagen, Tokyo, Japan). Quantitative real-time polymerase chain reaction was performed using SYBR Select Master Mix’s method (Applied Biosystem, Life Technologies, Foster City, CA, USA). The conditions of RT-qPCR were 50 °C for 2 min, 95 °C for 10 min, followed by 40 cycles (denaturing at 95 °C for 15 s, annealing at 60 °C for 1 min, and extension at 72 °C for 15 s). A melting curve was then generated to assess the specification of the PCR amplification. The geometric mean of housekeeping gene *18S* was used as an internal control to normalize the variability in the expression levels. The sequences of primers used are given in Table 2.

### 4.15. Bioinformatics Analysis

The structural coordinates of mortalin, p53, and PARP1 were downloaded from RCSB protein data bank (PDB ID: 4KBO, 1OLG, and 5DS3, respectively). The structures were prepared for docking using protein preparation wizard of the Maestro Suite (Biologics Suite 2018-3, Schrödinger, LLC, NY, 2018). This mainly involved the addition of missing disulphide bonds, removal of water molecules, addition of missing hydrogen atoms, filling of missing amino acids side chains, and optimization of hydrogen bonds. OPLS3e forcefield was then used for restrained minimization until the average root mean square deviation (RMSD) of the non-hydrogen atoms converged to 0.30 Å. It also allowed sufficient movement of heavy atom to relax strained bonds, angles, and clashes. The 3D structure of Mortaparib^Plus^, prepared using Marvin Sketch (ChemAxon, Budapest, Hungary), was subjected to LigPrep module of the Schrodinger suite to prepare it for docking studies. Tautomers and stereoisomers were generated in all possible ionization states, and the energy of the generated ligand conformations was then minimized using OPLS3e force field.

The grid for docking Mortaparib^Plus^ to mortalin and p53 was generated using the residues involved in their interaction (p53 binding site in mortalin: 260-288 aa; mortalin binding site in p53: 323-337 aa). For PARP1, the grid was generated at the catalytic site, following the known PARP1 inhibitors. Docking was performed using the extra-precision flexible-docking protocol of Glide (Biologics Suite 2018-3, Schrödinger, LLC, NY, 2018). The binding stability of the docked complexes was further studied using molecular dynamics simulations. Desmond with OPLS3e force field from Schrodinger was used to stimulate all protein–ligand complexes in the presence of explicit water molecules. Each protein–ligand complex was solvated with TIP4P water model in an orthorhombic periodic boundary box. To prevent interaction of the protein complex with its own periodic image, the distance between the complex and the box wall was kept 10 Å. The system was then neutralized by addition of ions. Energy of the prepared systems was minimized for 5000 steps using steepest descent method or until a gradient threshold of 25 kcal/mol/Å was achieved. The equilibrated system was then subjected to 100 ns simulation in NPT ensemble with 300 K temperature, constant pressure of 1atm, and time step of 2 fs. An average structure generated using the molecular conformations spanned over the stable stimulation trajectory was then used for detailed interaction analyses.

### 4.16. Statistical Analysis

Data from three or more independent experiments were expressed as mean ± standard deviation. Unpaired *t*-test (GraphPad Prism GraphPad Software, San Diego, CA, USA) has been performed to determine the degree of significance between the control and the treated experimental samples. Statistical significance was defined as significant (* *p* value ≤ 0.05), very significant (** *p* value ≤ 0.01), and highly significant (*** *p* value ≤ 0.001).

## 5. Conclusions

Morataprib^Plus^ prevents the interaction of mortalin with p53 resulting in the activation of growth arrest and apoptosis mediated by activation of p21^WAF1^ or BAX and PUMA signalling, respectively. Mortaparib^Plus^-induced cytotoxicity to cancer cells is mediated by multiple mechanisms that included the inhibition of PARP1, up-regulation of p73, and down-regulation of mortalin and CARF proteins that play critical roles in carcinogenesis. Mortaparib^Plus^ is a novel multimodal candidate anticancer drug that warrants further experimental and clinical attention.

## Figures and Tables

**Table 1:** Constitution of trapping assay buffers.

No.	Buffer Name	Composition
I	Hypotonic buffer	100 mM MES-NaOH pH 6.4, 1 mM EDTA, 0.5 mM MgCl_2_, 30% sucrose in Mili-Q H_2_O
II	Buffer A	50 mM HEPES-NaOH pH 7.5, 100 mM KCl, 2.5 mM MgCl_2_, 0.05% Triton X-100
III	Buffer B	50 mM HEPES-NaOH pH 7.5, 250 mM KCl, 2.5 mM MgCl_2_, 0.05% Triton X-100
IV	Buffer C	50 mM HEPES-NaOH pH 7.5, 500 mM KCl, 2.5 mM MgCl_2_, 0.1% Triton X-100
V	Buffer D	Buffer A, 5 mM CaCl_2_, Micrococcal protease inhibitor three-unit (Roche Diagnostic GmbH, Mannheim, Germany)

**Table 2:** Sequences of primers used for RT-PCR.

Gene (Human)	Primer Sequence (5′-3′)
*p53* forward	GTTCCGAGAGCTGAATGAGG
*p53* reverse	TCTGAGTCAGGCCCTTCTGT
*Mortalin* forward	AGCTGGAATGGCCTTAGTCAT
*Mortalin* reverse	CAGGAGTTGGTAGTACCCAAATC
*CDKN1A* (*p21^WAF1/CIP1^*) forward	GAGGCCGGGATGAGTTGGGAGGAG
*CDKN1A* (*p21^WAFI/CIP1^*) reverse	CAGCCGGCGTTTGGAGTGGTAGAA
*18S* forward	CAGGGTTCGATTCCGTAGAG
*18S* reverse	CCTCCAGTGGATCCTCGTTA

## Data Availability

All datasets used and/or analysed during the current study are available in the manuscript and supplementary information files (Supplementary Figures S5–S21).

## Supplementary Materials

The following are available online at https://www.mdpi.com/2072-6694/13/4/835/s1, Figure S1: Identification of Mortaparib^Plus^ as a novel small molecule. Structural homology of Mortaparib^Plus^ with several pre-clinically and clinically known molecules used for colorectal cancer therapy is shown. Figure S2: Site of interaction of Mortaparib^Plus^ with p53-binding site of mortalin and PARP1 at different time instances during the Molecular Dynamic Simulations. Mortaparib^Plus^-Mortalin complex was found to be quite stable as observed in the structures equally spanned over the simulation trajectory (A). Mortaparib^Plus^ did not deviate much from its docked orientation and stably stayed inside the catalytic pocket of PARP1 throughout the simulation trajectory (B). Figure S3: Interaction of Mortaparib^Plus^ with different p53 variants. (A) Mortaparib^Plus^ was found to interact at the same site in the wild type and mutant forms of p53 (R273H, S241F and R248W). The point of mutation in shown in sphere representation and the mortalin binding site has been highlighted in purple. As shown, the mutated site in each of the p53 variant lies far from the mortalin binding interface and hence does not significantly effects the binding energy as represented by docking score and MM/GBSA ΔG values (B). Figure S4: Bar charts showing the quantification of PARP1, cleaved PARP1 and PAR levels in control and Mortaparib^Plus^-treated colorectal cancer cells as analyzed by Immunocytochemistry (Figure 8C,D). The quantified data represents mean ± SD obtained from independent biological replicates; *p*-values were calculated using unpaired Student’s *t*-test. * < 0.05, ** < 0.01 and *** < 0.001 represent significant, very significant and very very significant, respectively.

## Abbreviations

ATM	Ataxia telangiectasia mutated
ATR	Ataxia telangiectasia and Rad3 related
BAX	Bcl-2-associated X protein
Bcl-xL	B-cell lymphoma-extra large
BRCA1/2	Breast cancer gene 1/gene 2
CDK4	Cyclin-dependent kinase 4
CDKN1A	Cyclin-dependent kinase inhibitor 1A
CIP1	Cyclin-dependent kinase inhibitor 1
CO_2_	Carbon dioxide
ddH2O	Double-distilled H2O
DNA	Deoxyribonucleic acid
E2F1	E2F transcription factor 1
EDTA	Ethylenediaminetetraacetic acid
EMT	Epithelial to mesenchymal transition
FDA	Food and Drug Administration
GRP75	Glucose-regulated protein 75
HEPES	4-(2-hydroxyethyl)-1-piperazineethanesulfonic acid
HSPA9	Heat-shock protein family A (Hsp70) member 9
HT-29	Human colorectal adenocarcinoma cell line
IgG	Immunoglobulin G
KCL	Potassium chloride
kDa	Kilodaltons
LoVo	Human colon adenocarcinoma cell line
LS123	Human colon adenocarcinoma cell line
MES	2-(N-morpholino) ethanesulfonic acid
MgCl_2_	Magnesium chloride
MRC5	Medical Research Council cell strain 5 lung fibroblast
mM	Millimeter
mRNA	Messenger RNA
Mthsp70	Mitochondrial 70-kDa heat-shock protein
NaOH	Sodium hydroxide
NP-40	Nonidet P-40
PBS	Phosphate-buffered saline
PCR	Polymerase chain reaction
PUMA	p53 up-regulated modulator of apoptosis
RCSB	Research Collaboratory for Structural Bioinformatics.
RIPA	Radioimmunoprecipitation assay
RNA	Ribonucleic acid
RT-qPCR	Quantitative reverse transcription PCR
shRNA	Short hairpin ribonucleic acid
TIG3	Tokyo Institute of Gerontology-3 lung fibroblast
μM	Micromolar
