# Supplementary material for: Identification and Characterization of MortaparibPlus—A Novel Triazole Derivative That Targets Mortalin-p53 Interaction and Inhibits Cancer-Cell Proliferation by Wild-Type p53-Dependent and -Independent Mechanisms
Source: Cancers (Basel). 2021 Feb 17;13(4):835. doi: 10.3390/cancers13040835 (PMC7921971; doi:10.3390/cancers13040835)
Supplement: Supplementary file 1 [file cancers-13-00835-s001.pdf]

SUPPLEMENTARY INFORMATION

Identification and characterization of Mortaparib^{Plus} - a novel triazole derivative that targets mortalin-p53 interaction and inhibits cancer cell proliferation by wild type p53-dependent and -independent mechanisms

Anissa Nofita Sari[1,2$], Ahmed Elwakeel[1,2$], Jaspreet Kaur Dhanjal[1], Vipul Kumar[3],
Durai Sundar[3], Sunil C. Kaul[1] and Renu Wadhwa[1,2*]

[1]AIST-INDIA DAILAB, National Institute of Advanced Industrial Science & Technology (AIST), Central 5-41, Tsukuba 305-8565, Japan

[2]School of Integrative & Global Majors (SIGMA), University of Tsukuba, Tsukuba 305-8577, Japan

[3]DAILAB, Department of Biochemical Engineering & Biotechnology, Indian Institute of Technology (IIT) Delhi, Hauz Khas, New Delhi 110-016, India

[$]Anissa Nofita Sari and Ahmed Elwakeel contributed equally to this work.

*Correspondence: renu-wadhwa@aist.go.jp (R.W.). s-kaul@aist.go.jp (S.C.K.)
National Institute of Advance Industrial Science & Technology (AIST), Central 5-41, Higashi 1-1-1, Tsukuba, Ibaraki 305-8565, Japan
Tel.: +81-29-861-9464 (R.W.); (+81-29-861-6713) (S.C.K.)

Additional files

Figure S1: Identification of Mortaparib^{Plus} as a novel small molecule. Structural homology of Mortaparib^{Plus} with several pre-clinically and clinically known molecules used for colorectal cancer therapy is shown.

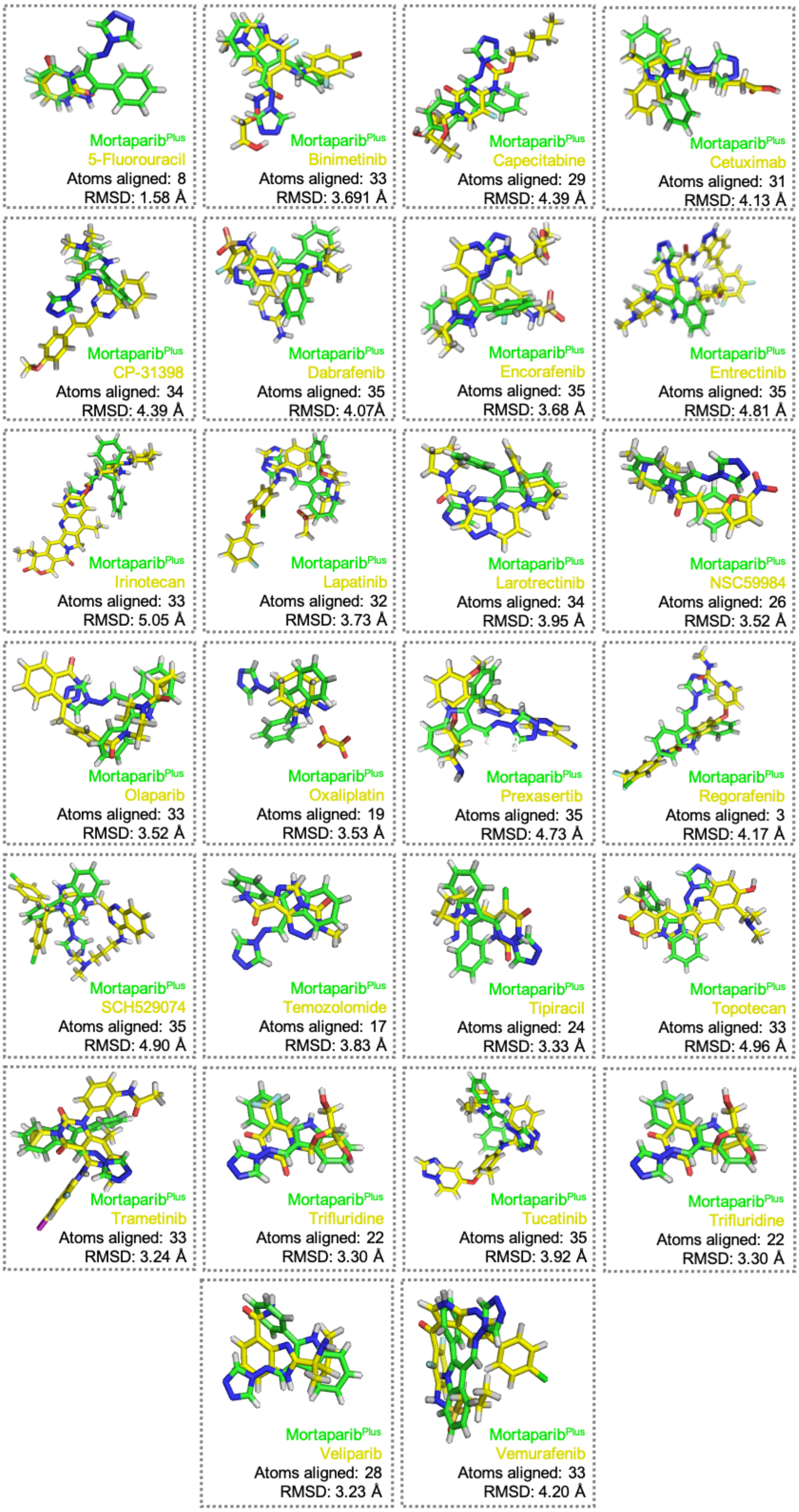

Figure S2: Site of interaction of Mortaparib^{Plus} with p53-binding site of mortalin and PARP1 at different time instances during the Molecular Dynamic Simulations. Mortaparib^{Plus}-Mortalin complex was found to be quite stable as observed in the structures equally spanned over the simulation trajectory (**A**). Mortaparib^{Plus} did not deviate much from its docked orientation and stably stayed inside the catalytic pocket of PARP1 throughout the simulation trajectory (**B**).

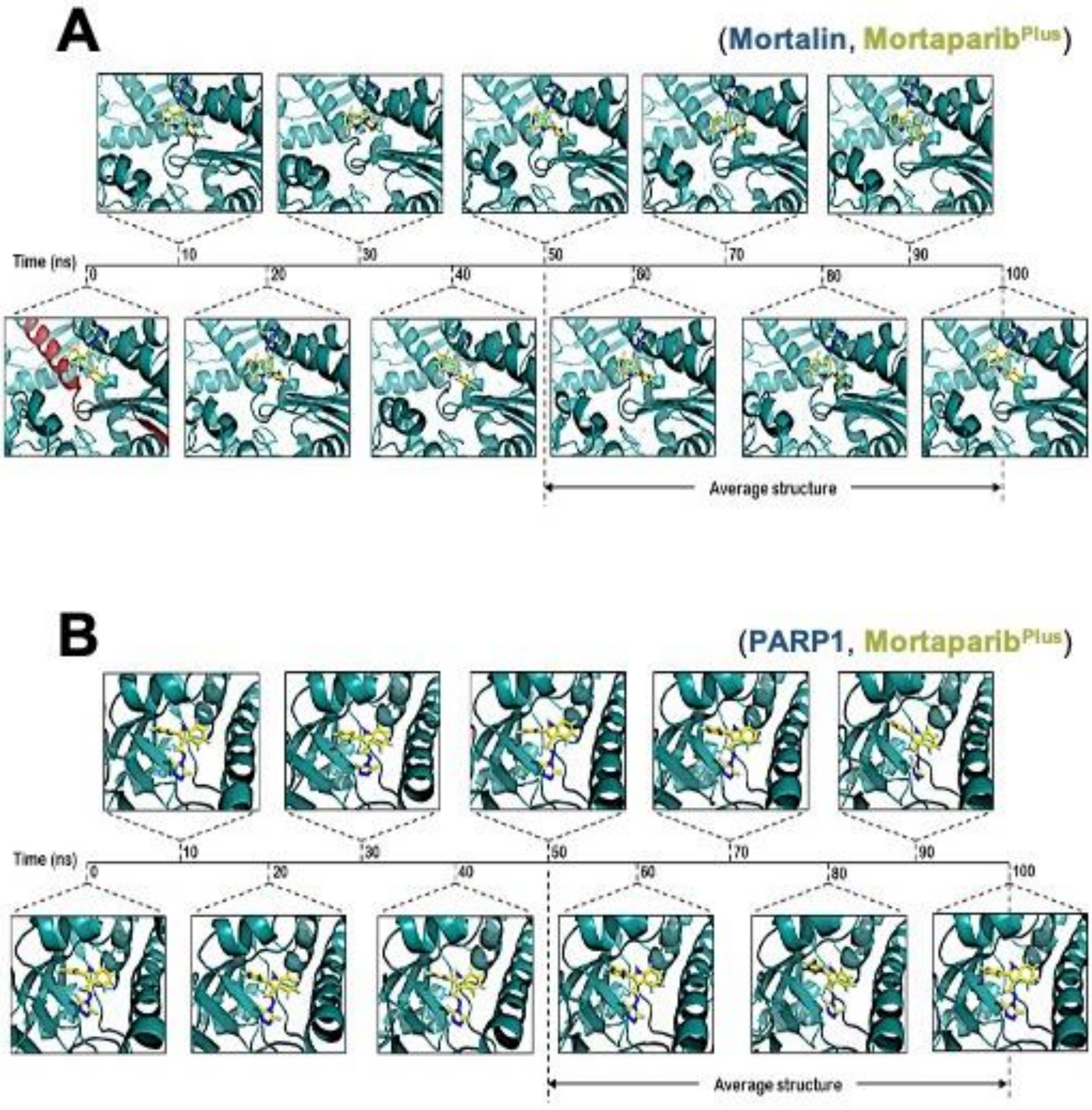

Figure S3. Interaction of Mortaparib^{Plus} with different p53 variants. **(A)** Mortaparib^{Plus} was found to interact at the same site in the wild type and mutant forms of p53 (R273H, S241F and R248W). The point of mutation in shown in sphere representation and the mortalin binding site has been highlighted in purple. As shown, the mutated site in each of the p53 variant lies far from the mortalin binding interface and hence does not significantly effects the binding energy as represented by docking score and MM/GBSA ΔG values **(B)**.

A

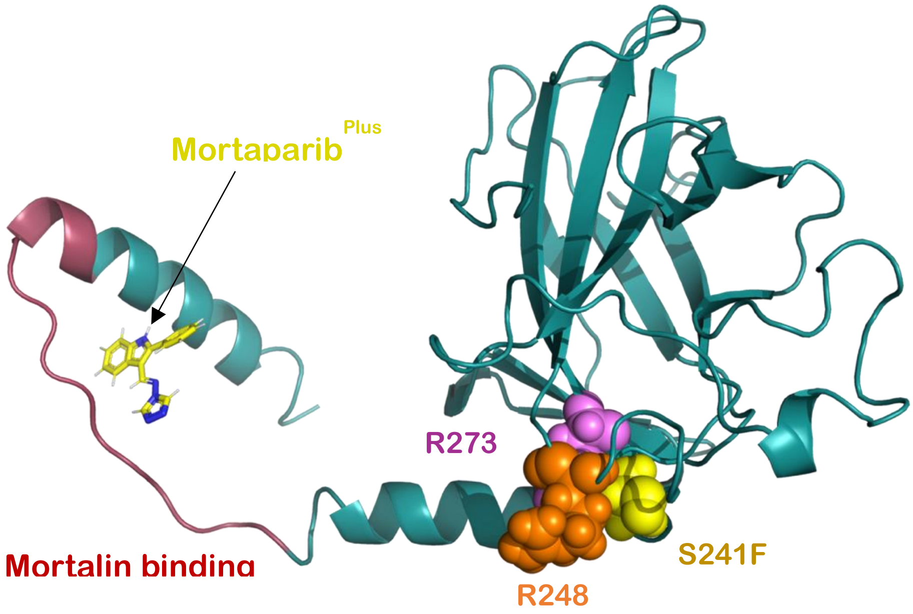

B

p53-mortaparib^{Plus} complex	Docking score (kcal/mol)	MM/GBSA ΔG (kcal/mol)
**Wild type p53**	**-3.60**	**-36.14**
**S241F**	**-2.07**	**-31.20**
**R248W**	**-2.76**	**-29.74**
**S273H**	**-1.56**	**-28.61**

Figure S4. Bar charts showing the quantification of PARP1, cleaved PARP1 and PAR levels in control and Mortaparib^{Plus}-treated colorectal cancer cells as analyzed by Immunocytochemistry (Figures 8C and 8D). The quantified data represents mean ± SD obtained from independent biological replicates; p-values were calculated using unpaired Student's t-test. *<0.05, **<0.01 and ***<0.001 represent significant, very significant and very very significant, respectively.

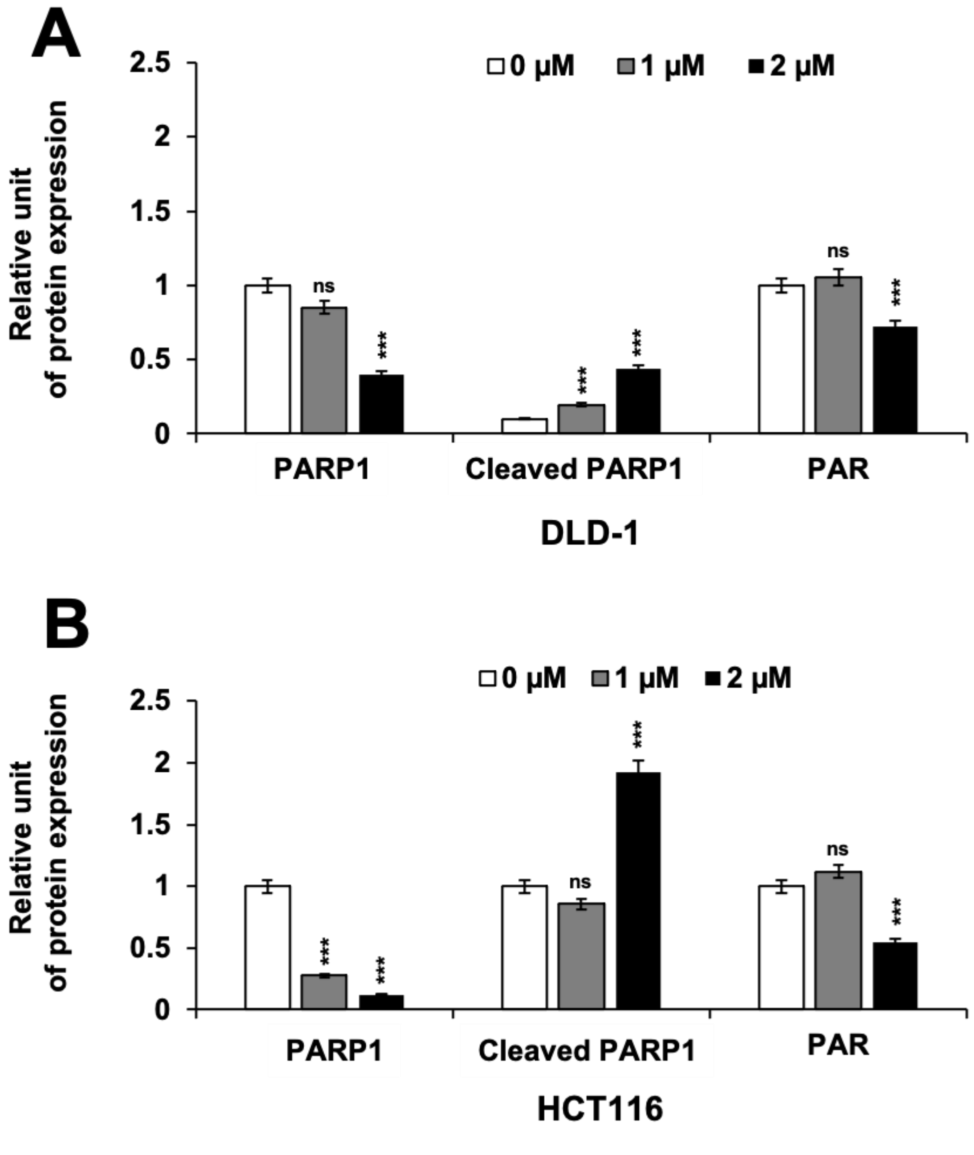

Full uncropped Western blots presented in Figs 2-8

Figure S5. Full uncropped Western blots for the protein of interest (p53) detected from both mortalin immunocomplexes and input of Mortaparib^{Plus}-treated and control DLD-1 cell lysates (**Figure 2B**). For the immunoprecipitated samples, mortalin bands were used to normalize an equal immunocomplexes. For the input samples, β-actin was used as an internal loading control.

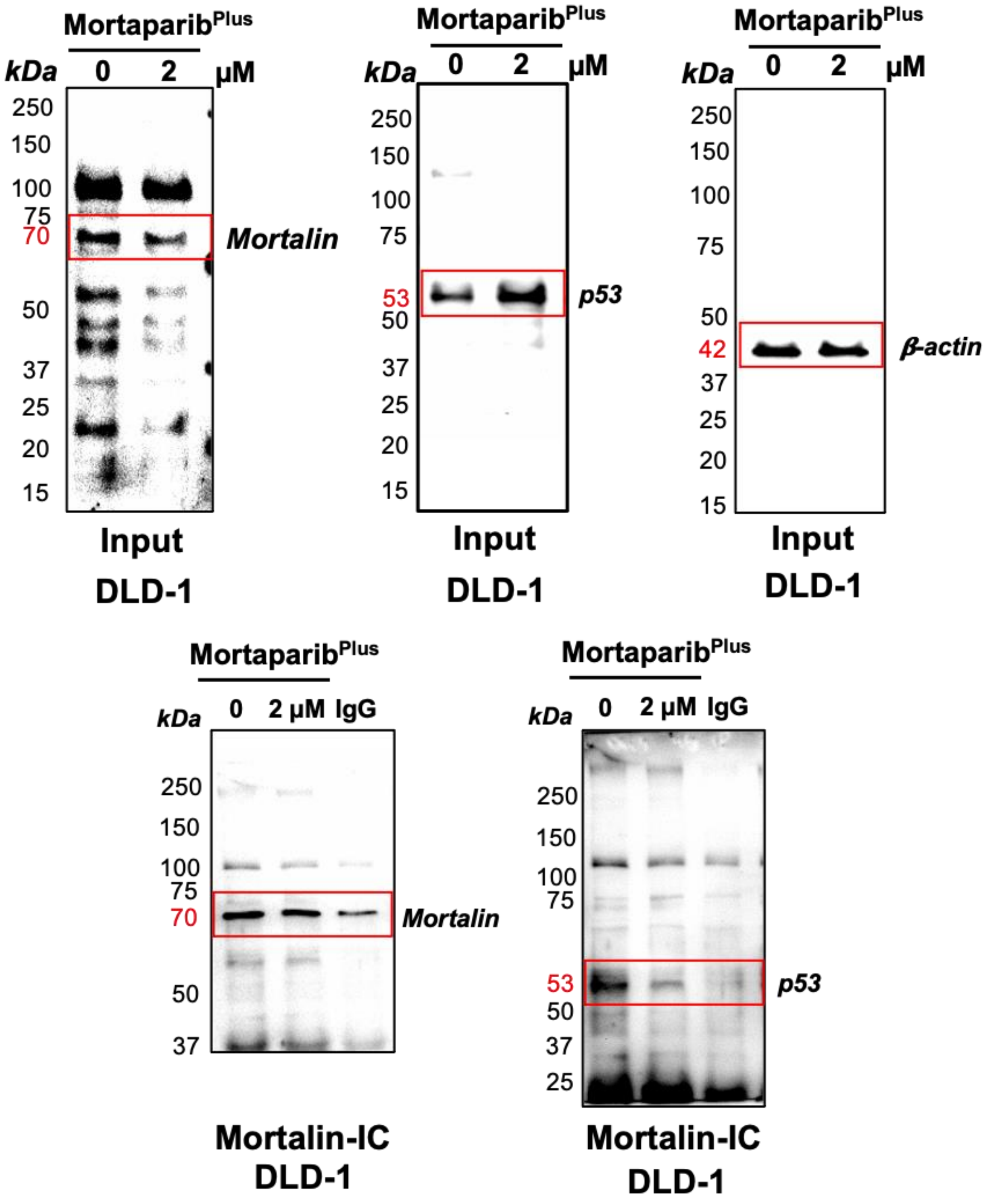

Figure S6. Full uncropped Western blots for the protein of interest (p53) detected from both mortalin immunocomplexes and input of Mortaparib[Plus]-treated and control HCT116 cell lysates (**Figure 2C**). For the immunoprecipitated samples, mortalin bands were used to normalize an equal immunocomplexes. For the input samples, β-actin was used as an internal loading control.

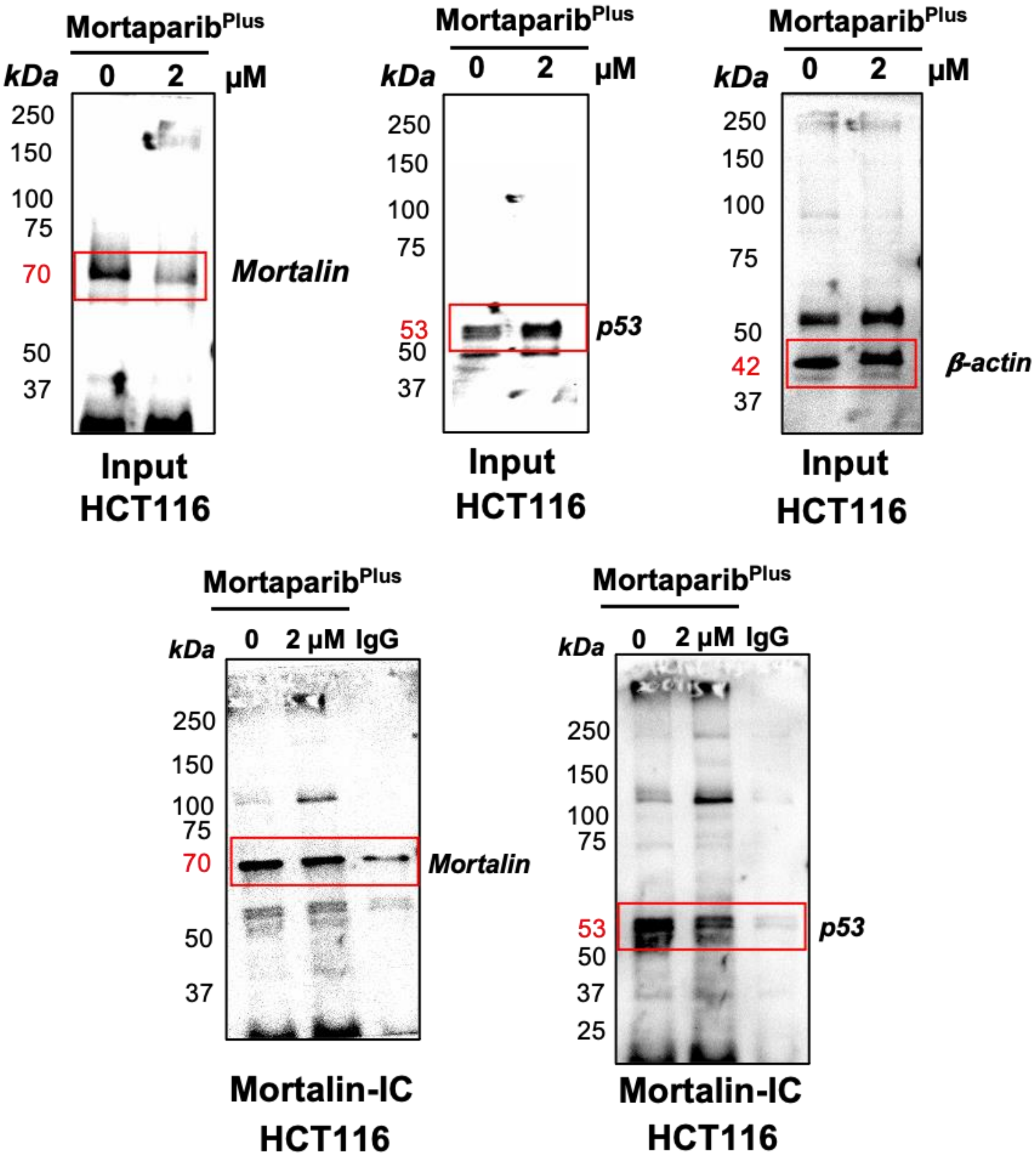

Figure S7. Full uncropped Western blots for the proteins of interest (p53 and mortalin) detected from Mortaparib^Plus-treated and control DLD-1 and HCT116 cell lysates (**Figure 3A and Figure 3B**). β-actin was used as an internal loading control.

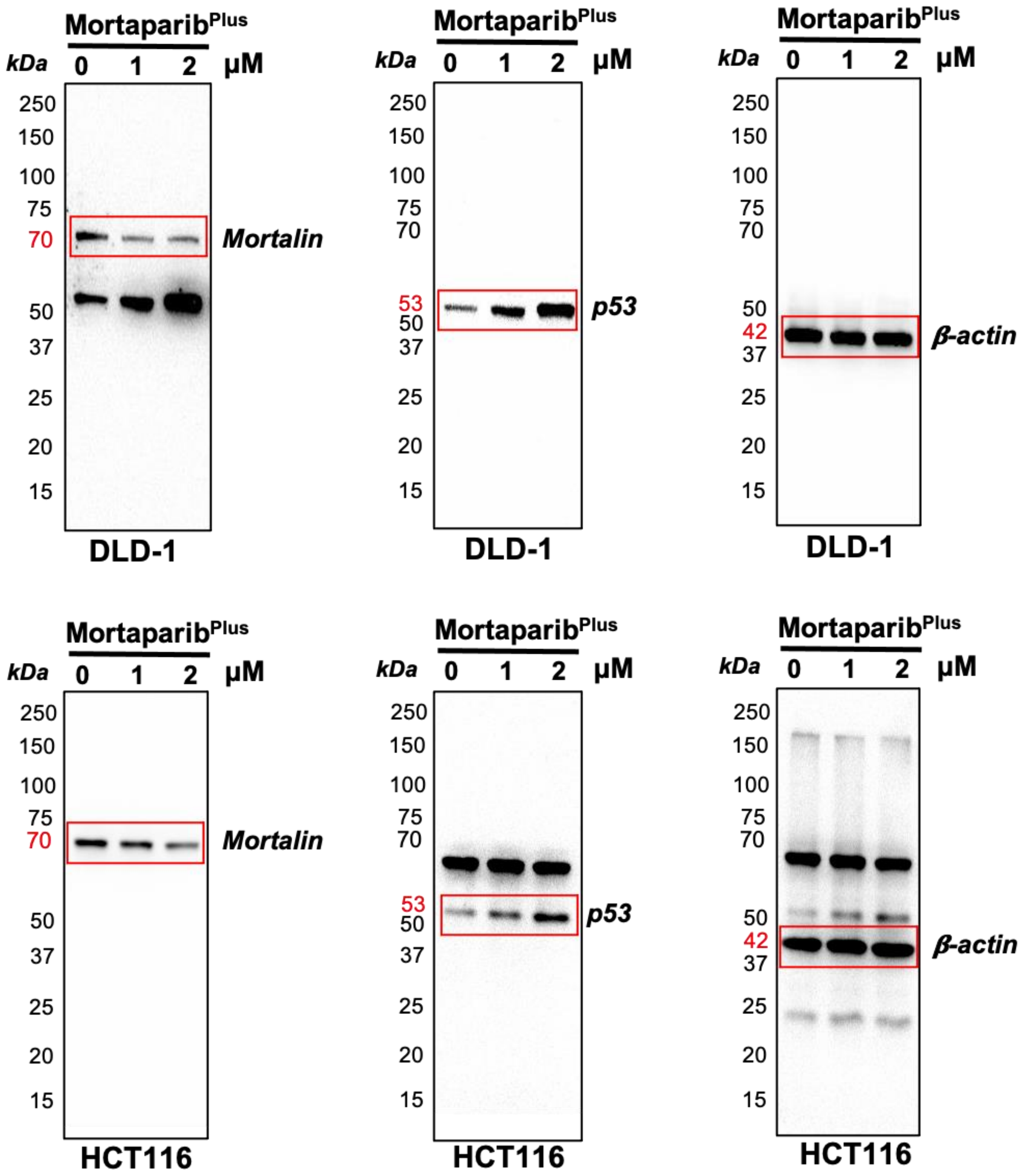

Figure S8. Full uncropped Western blots for the proteins of interest (PUMA, BAX, Bcl-xL and Caspase-3) detected from Mortaparib^{Plus}-treated and control DLD-1 cell lysates (**Figure 4C**). β-actin was used as an internal loading control.

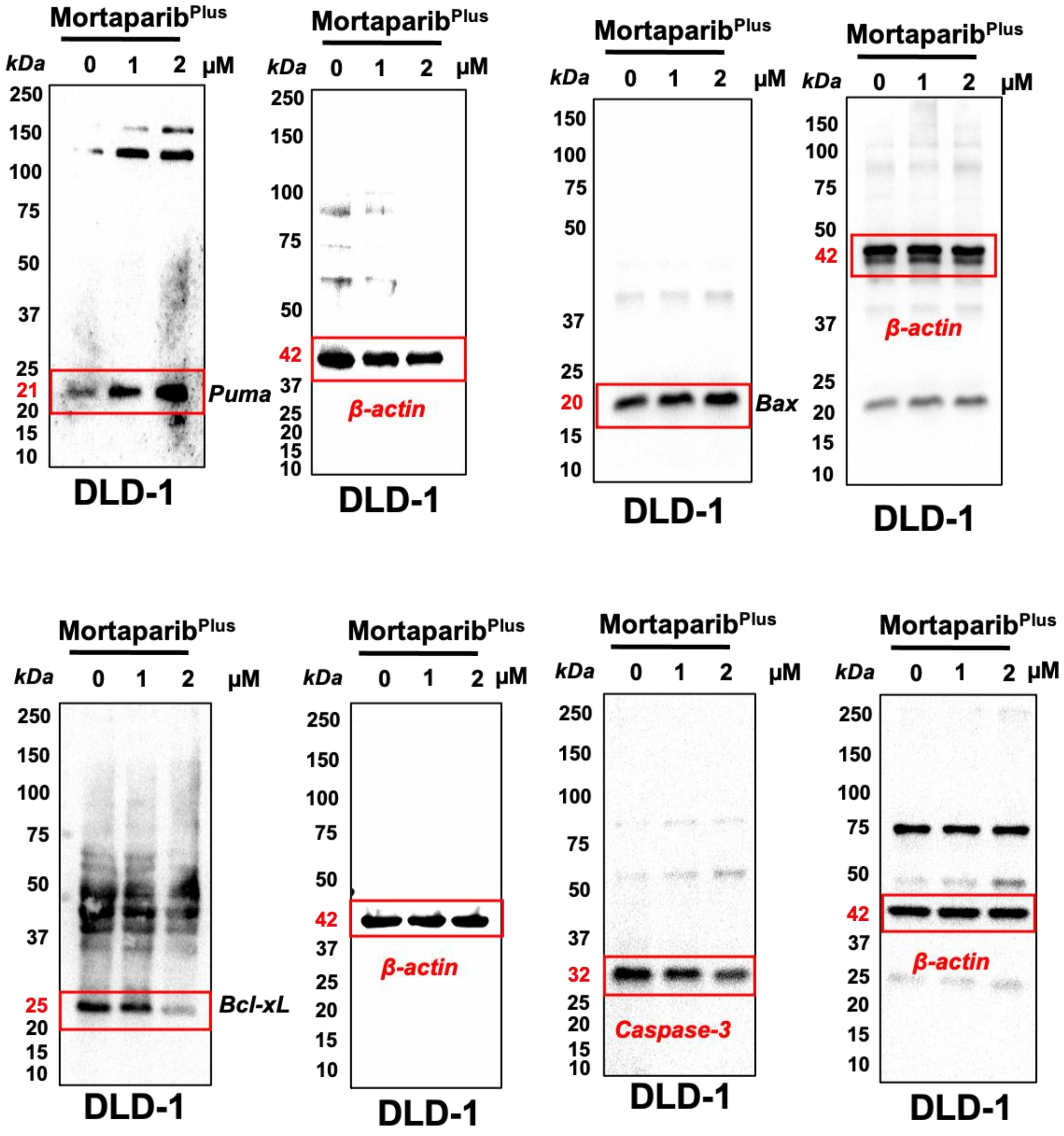

Figure S9. Full uncropped Western blots for the proteins of interest (Cleaved caspase-3, Caspase-9 and Caspase 7) detected from Mortaparib^{Plus}-treated and control DLD-1 cell lysates (**Figure 4C**). β-actin was used as an internal loading control.

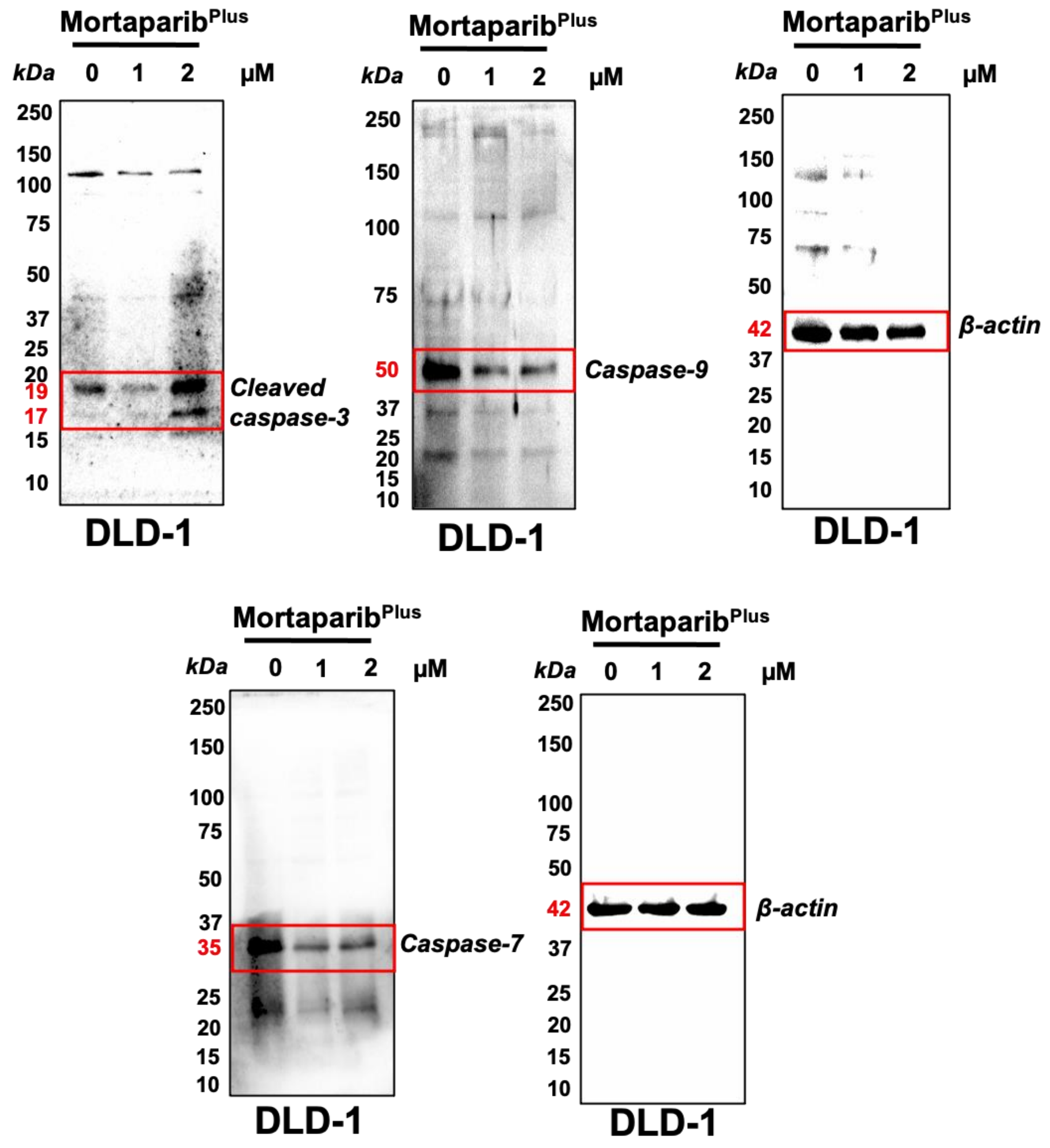

Figure S10. Full uncropped Western blots for the proteins of interest (PUMA, BAX, BcL-xL and Caspase 3) detected from Mortaparib^{Plus}-treated and control HCT116 cell lysates (**Figure 4D**). β-actin was used as an internal loading control.

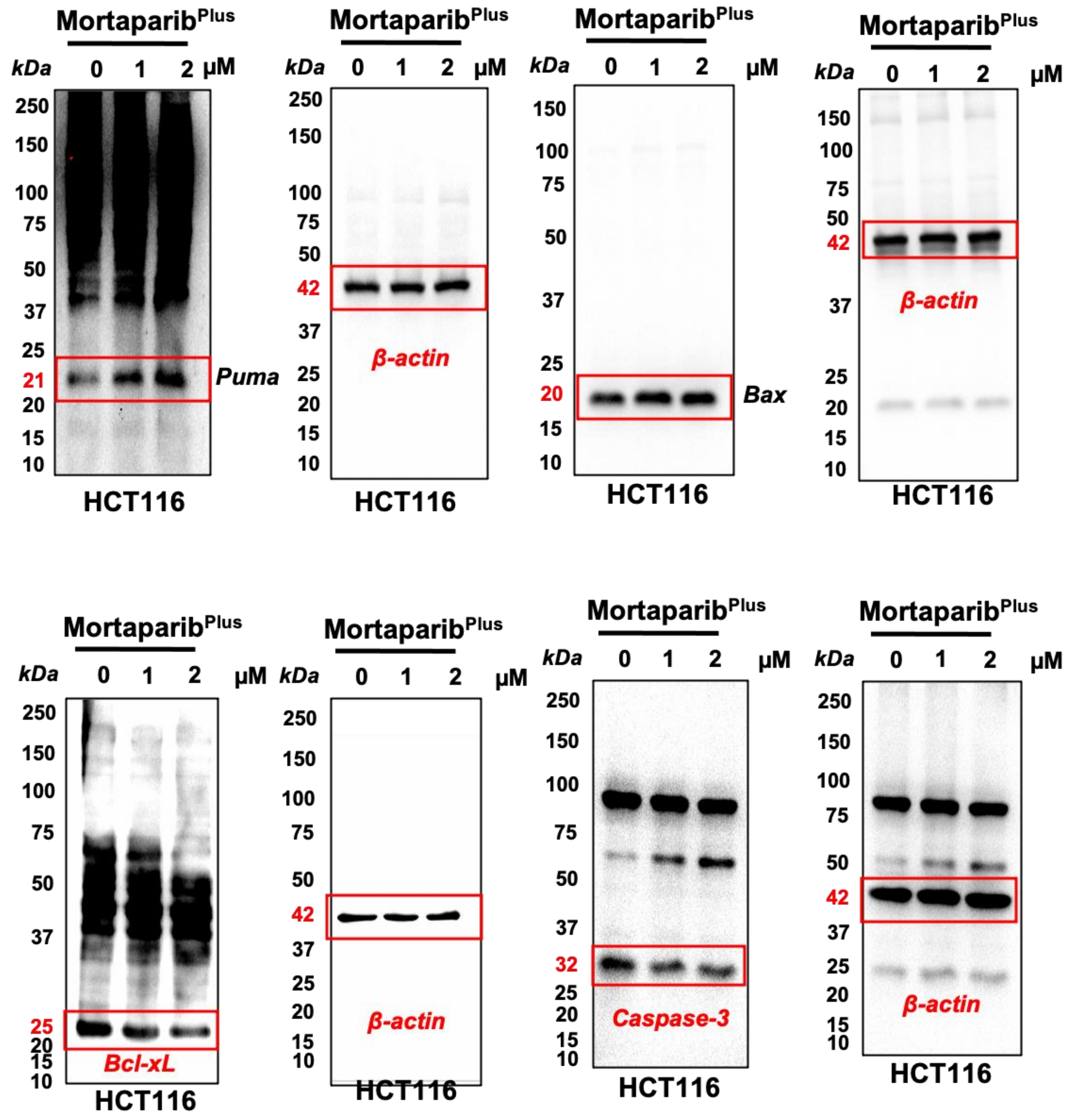

Figure S11. Full uncropped Western blots for the proteins of interest (Cleaved caspase-3, Caspase-9 and Caspase 7) detected from Mortaparib^{Plus}-treated and control HCT116 cell lysates (**Figure 4D**). β-actin was used as an internal loading control.

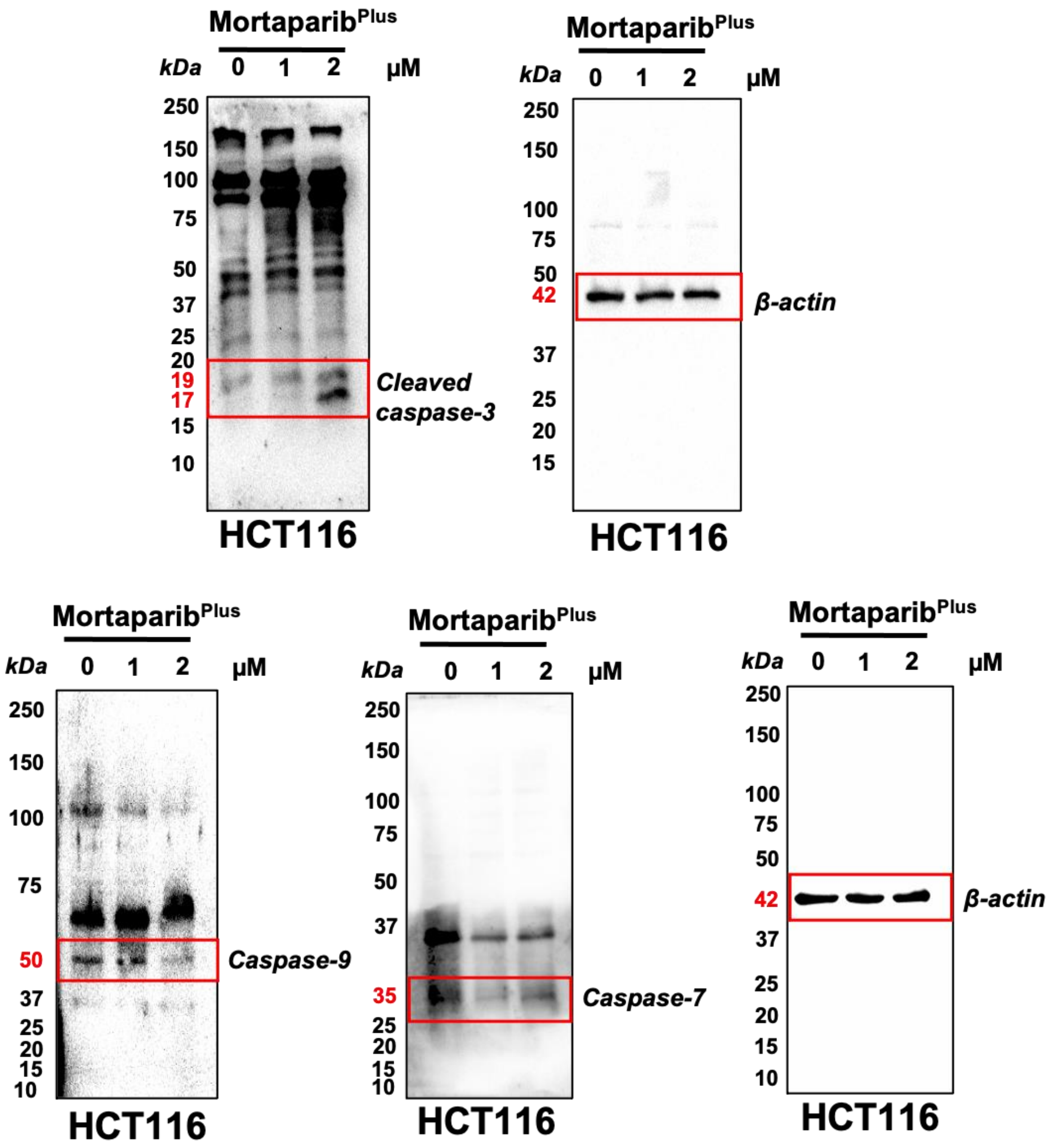

Figure S12. Full uncropped western blots for the proteins of interest (p21, CDK4, and Cyclin D1) detected from Mortaparib^Plus^-treated and control DLD-1 cell lysates (**Figure 5C**). β-actin was used as an internal loading control.

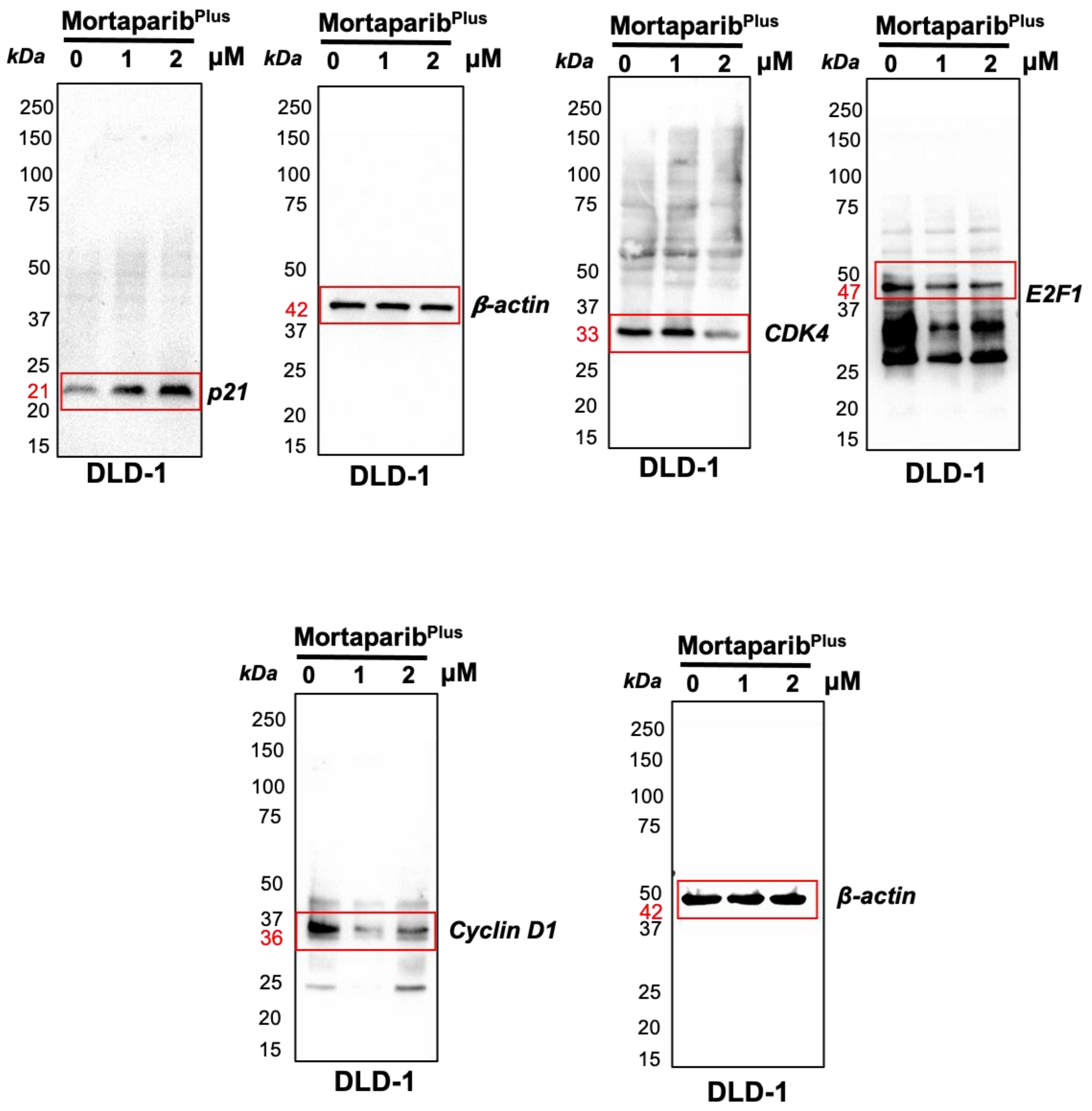

Figure S13. Full uncropped Western blots for the proteins of interest (p21, CDK4, and Cyclin D1) detected from Mortaparib^{Plus}-treated and control HCT116 cell lysates (**Figure 5D**). β-actin was used as an internal loading control.

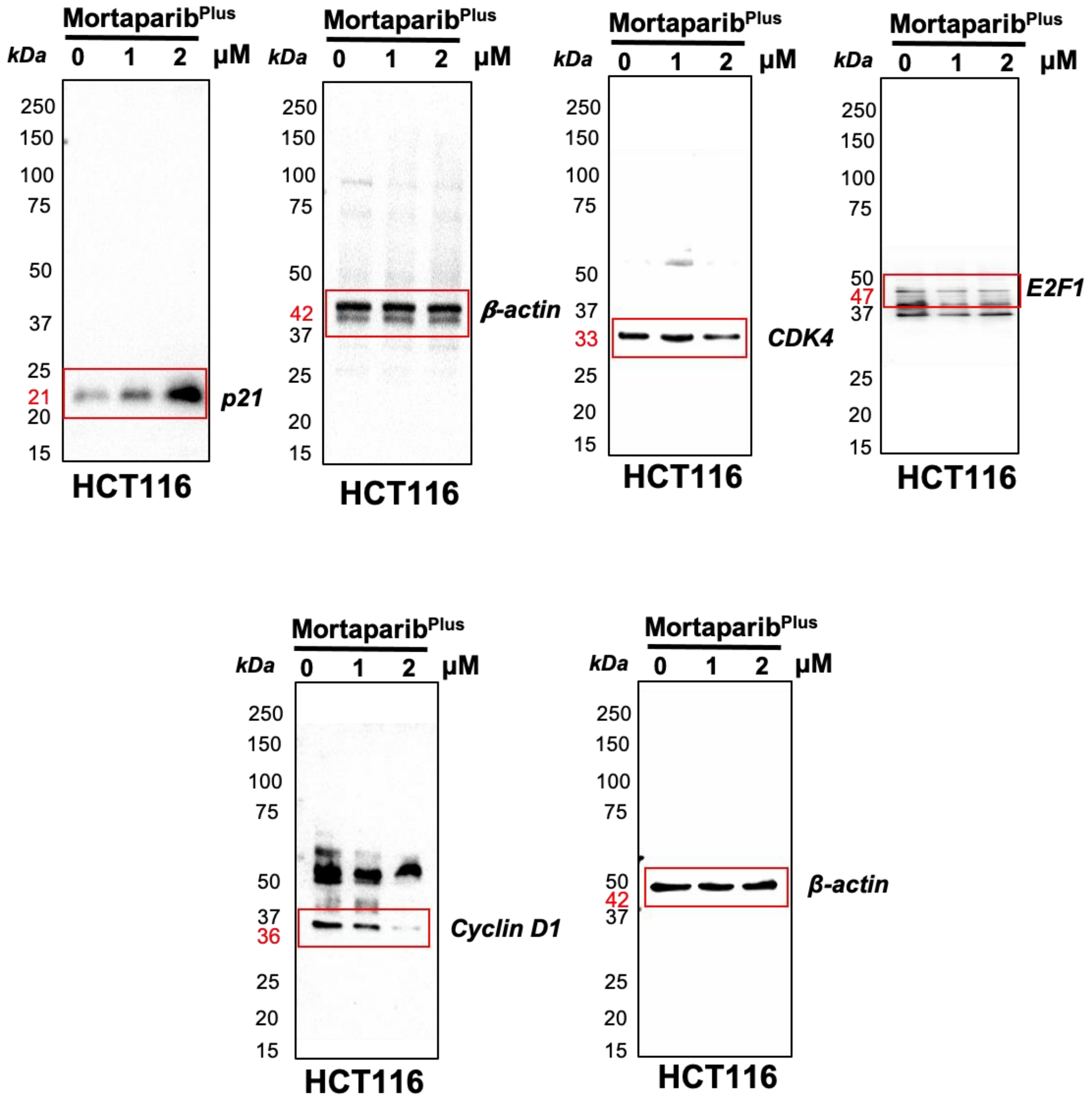

Figure S14. Full uncropped western blots for the proteins of interest (p73 and p63) detected from Mortaparib^{Plus}-treated and control DLD-1 cell lysates (**Figure 6A**). β-actin was used as an internal loading control**.**

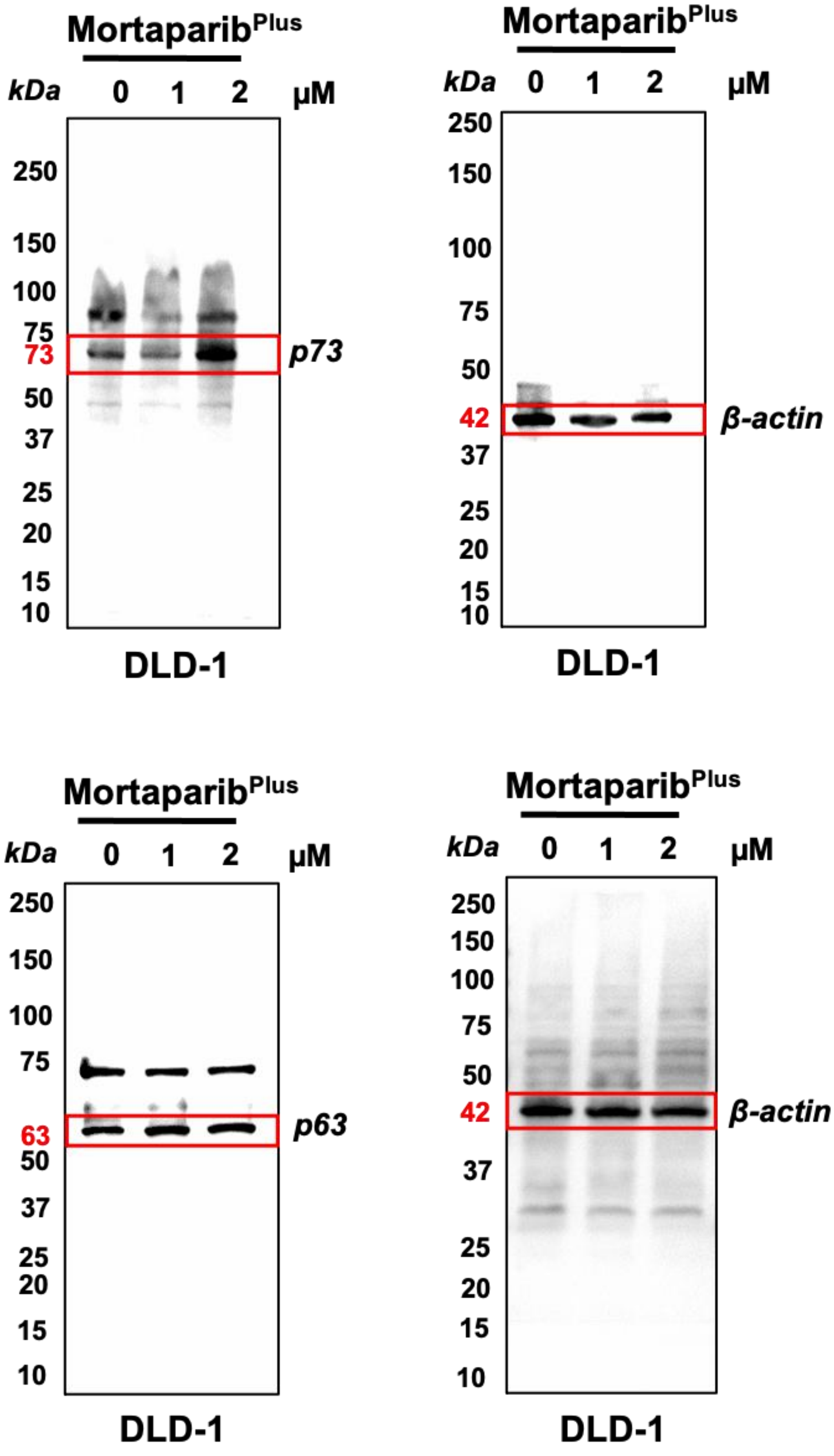

Figure S15. Full uncropped Western blots for the proteins of interest (p73 and p63) detected from Mortaparib^{Plus}-treated and control HCT116 cell lysates (**Figure 6B**). β-actin was used as an internal loading control.

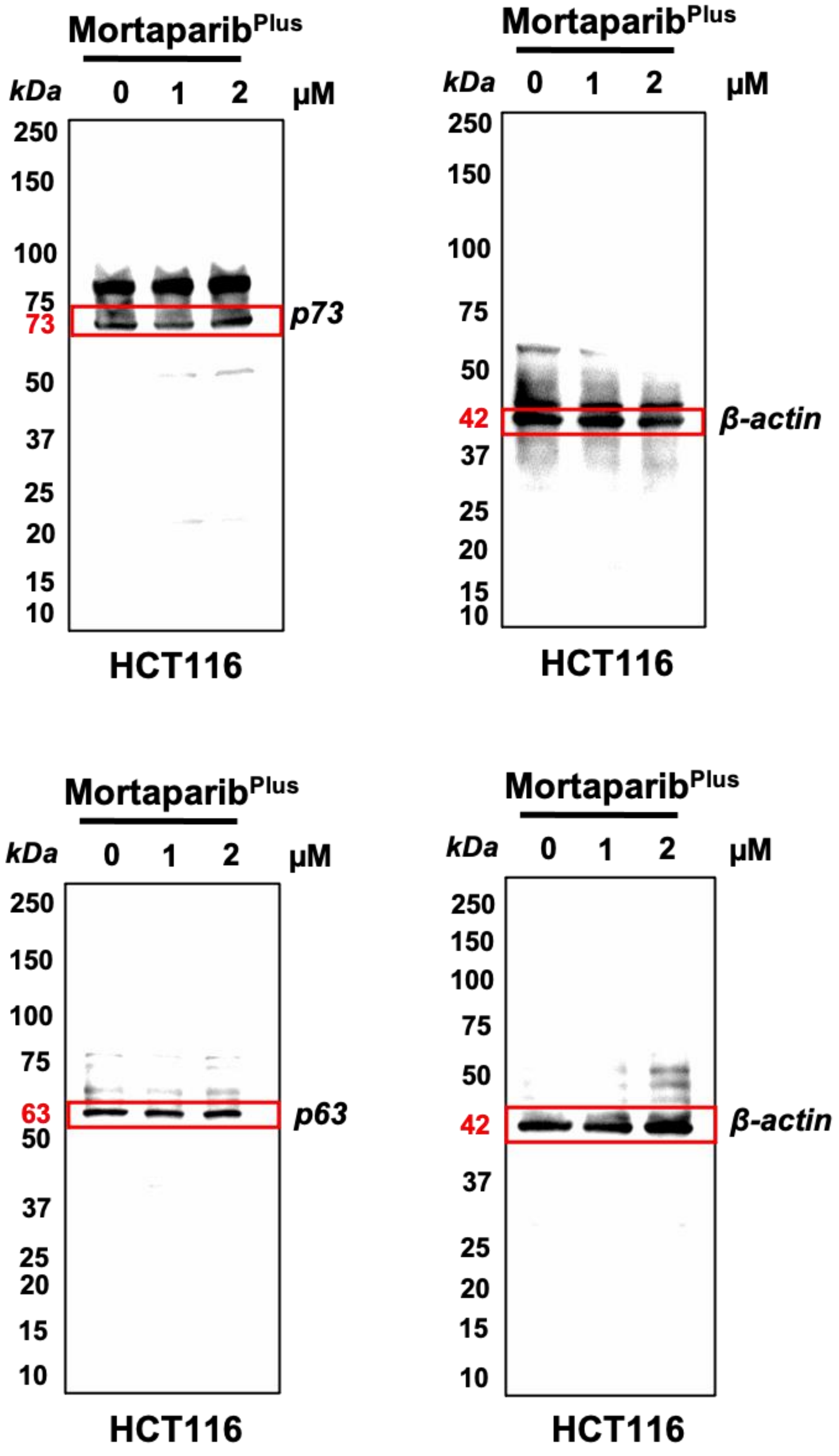

Figure S16. Full uncropped Western blots for the proteins of interest (CARF) detected from Mortaparib^{Plus}-treated and control DLD-1 and HCT116 cell lysates (**Figures 6E and 6F**). β-actin was used as an internal loading control.

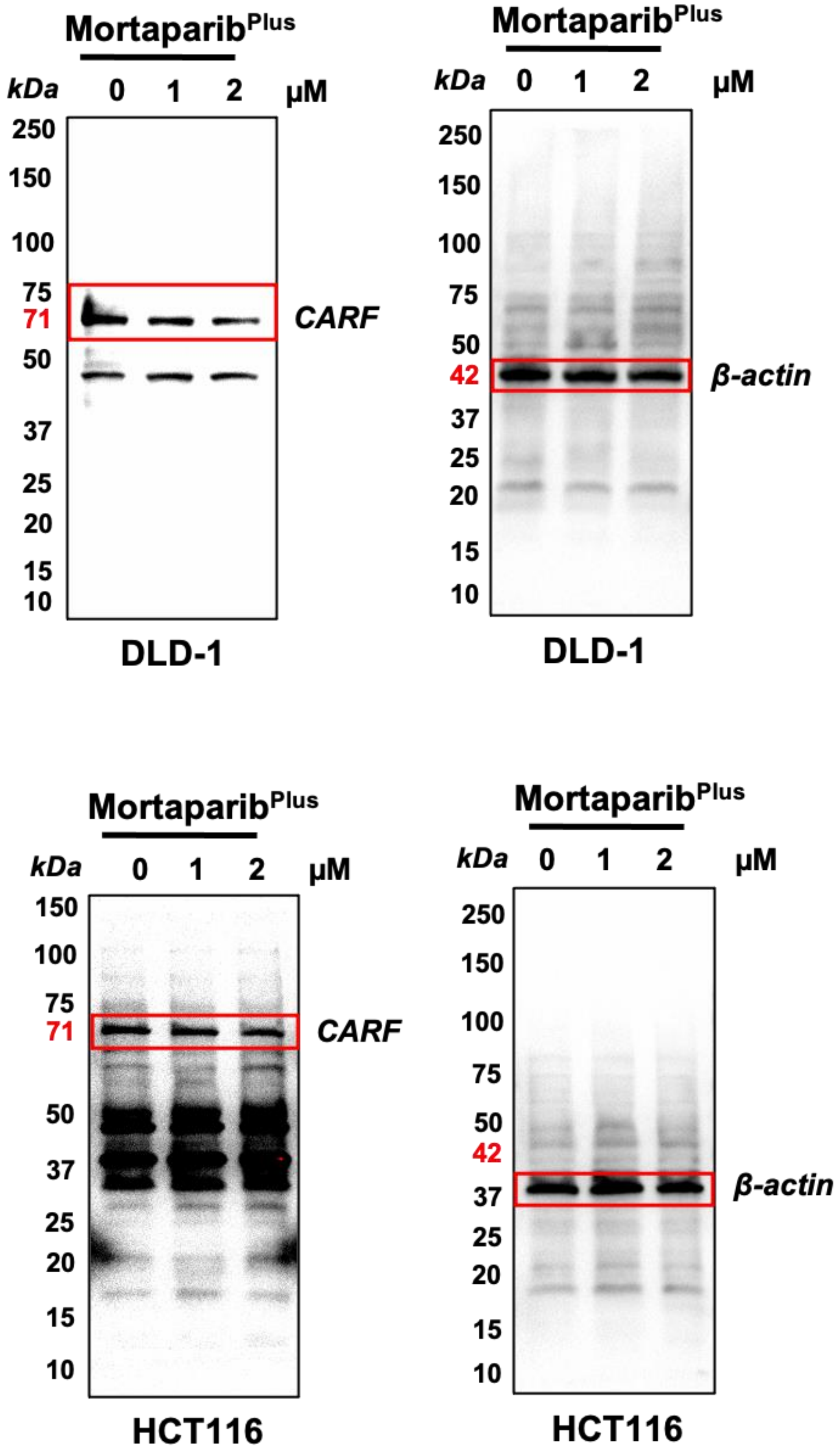

Figure S17. Full uncropped western blots for the PARP1-DNA complex detected from Mortaparib^{Plus}-treated and control DLD-1 cell lysates (**Figure 7B**). Histone-3 (H3) bands were used as a control.

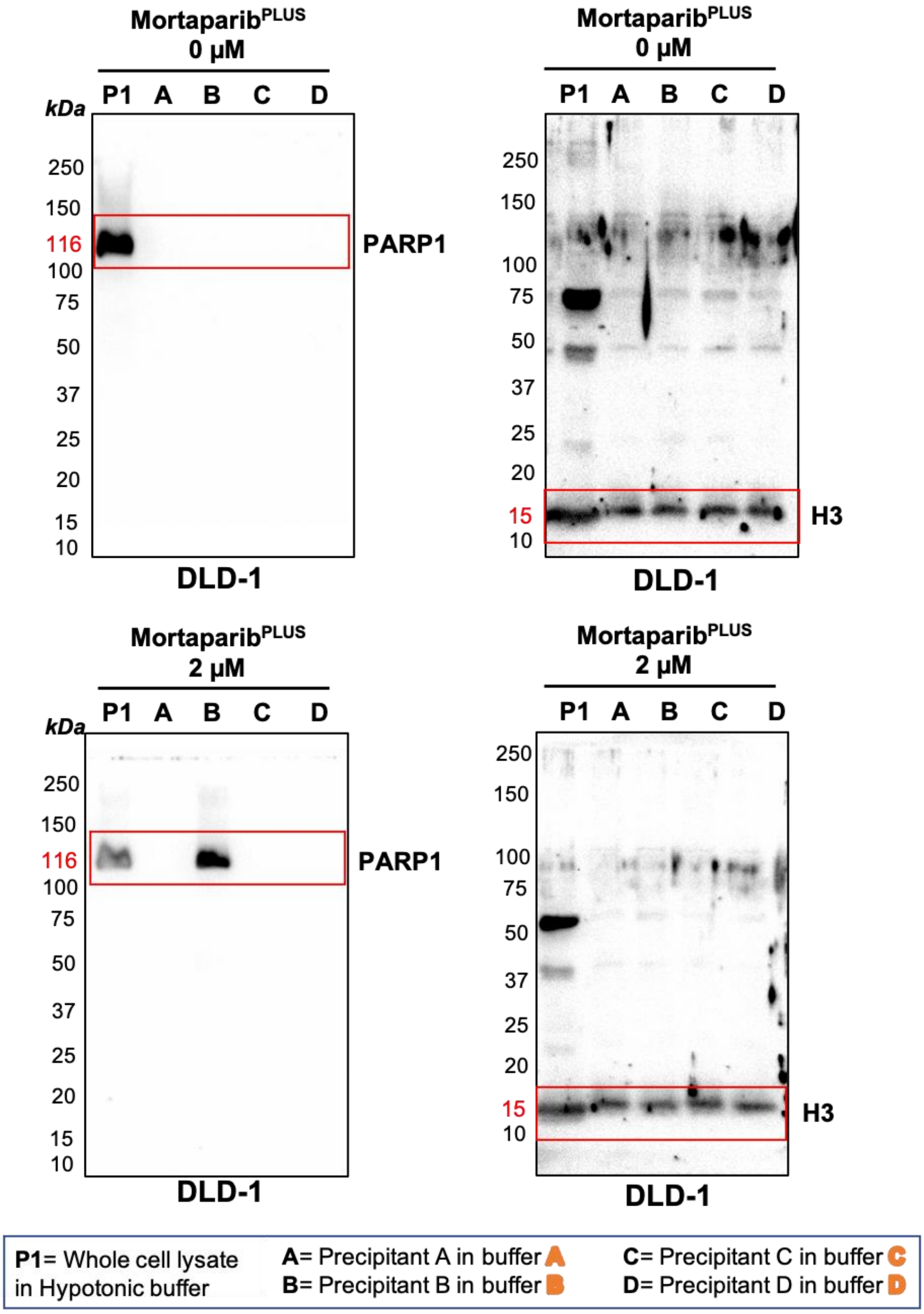

Figure S18. Full uncropped Western blots for the PARP1-DNA complex detected from Mortaparib^{Plus}-treated and control HCT116 cell lysates (**Figure 7C**). Histone-3 (H3) bands were used as a control.

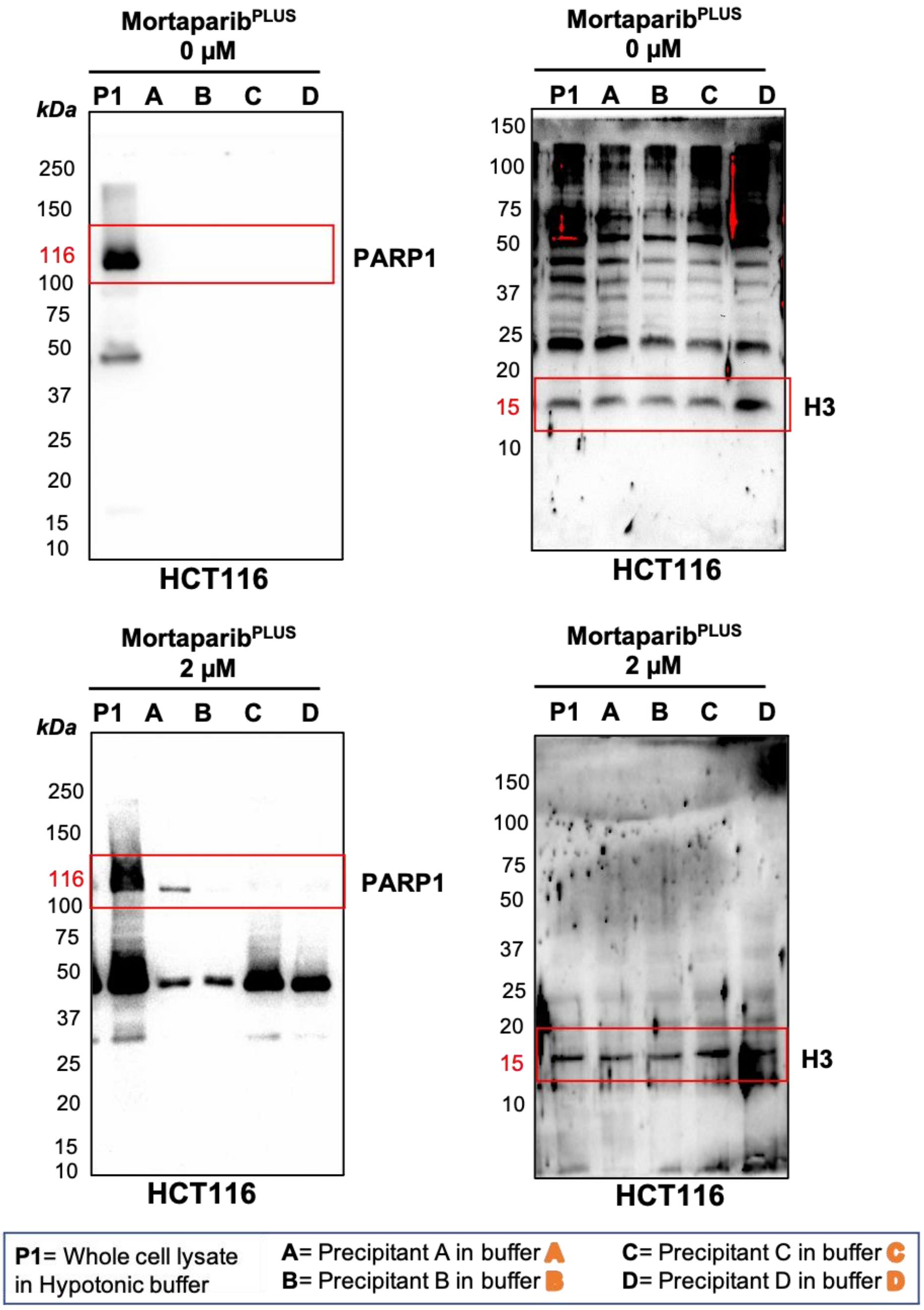

Figure S19. Full uncropped Western blots for the proteins of interest (**γ***H2Ax*) detected from Mortaparib[Plus]-treated and control DLD-1 and HCT116 cell lysates (**Figures 7D and 7E**). β-actin was used as an internal loading control**.**

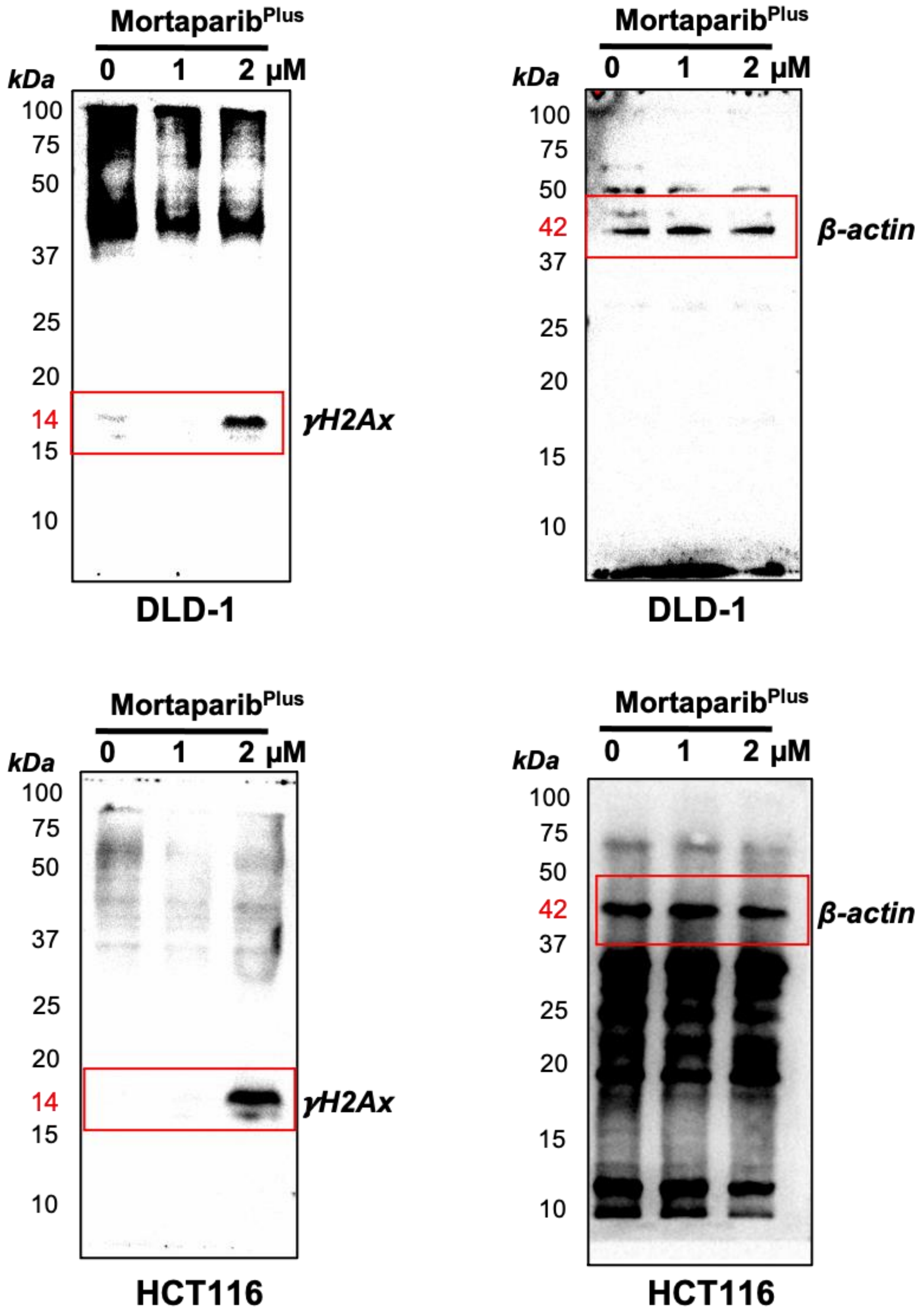

Figure S20. Full uncropped western blots for PARP1, Cleaved-PARP1 and PAR detected from Mortaparib^{Plus}-treated and control DLD-1 cell lysates (**Figure 8A**). β-actin was used as an internal loading control.

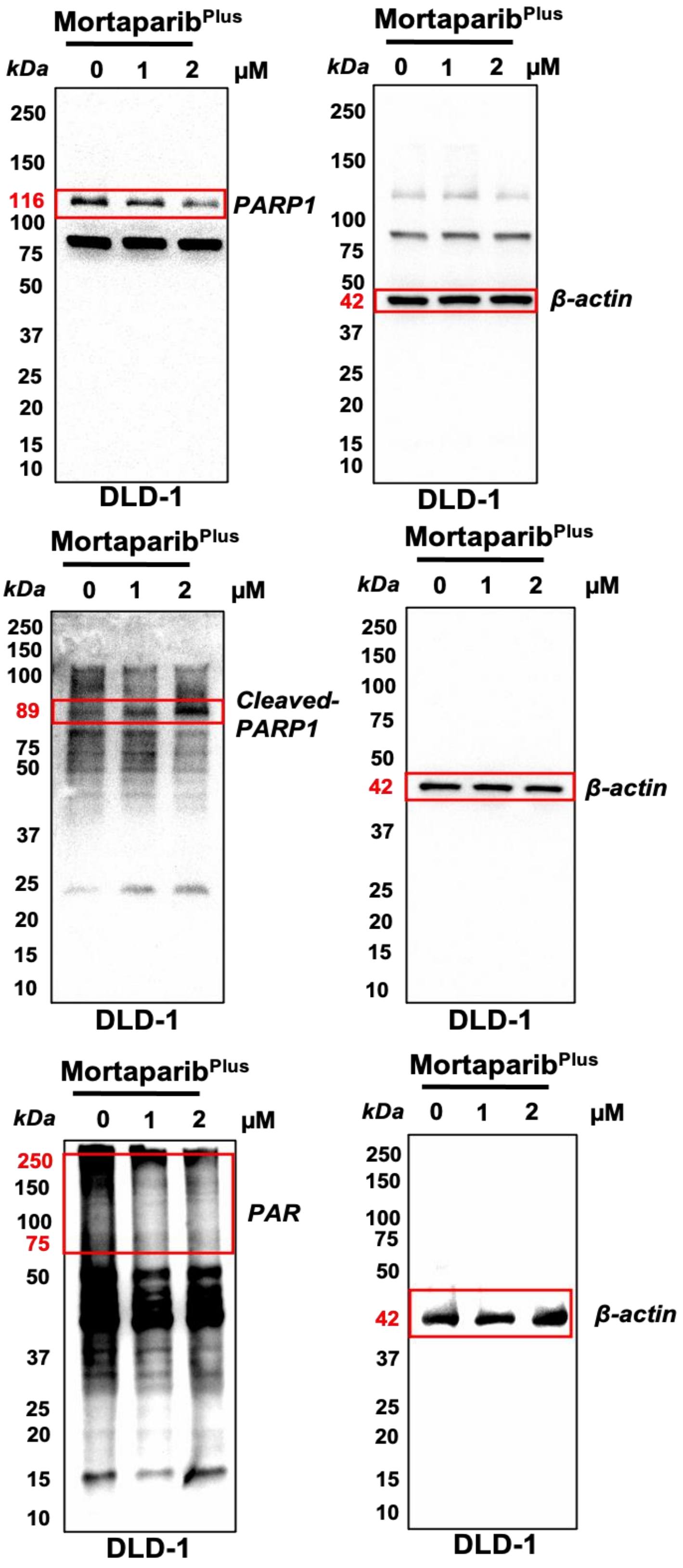

Figure S21. Full uncropped Western blots for PARP1, Cleaved-PARP1 and PAR detected from Mortaparib^{Plus}-treated and control HCT116 cell lysates (**Figure 8B**). β-actin was used as an internal loading control.

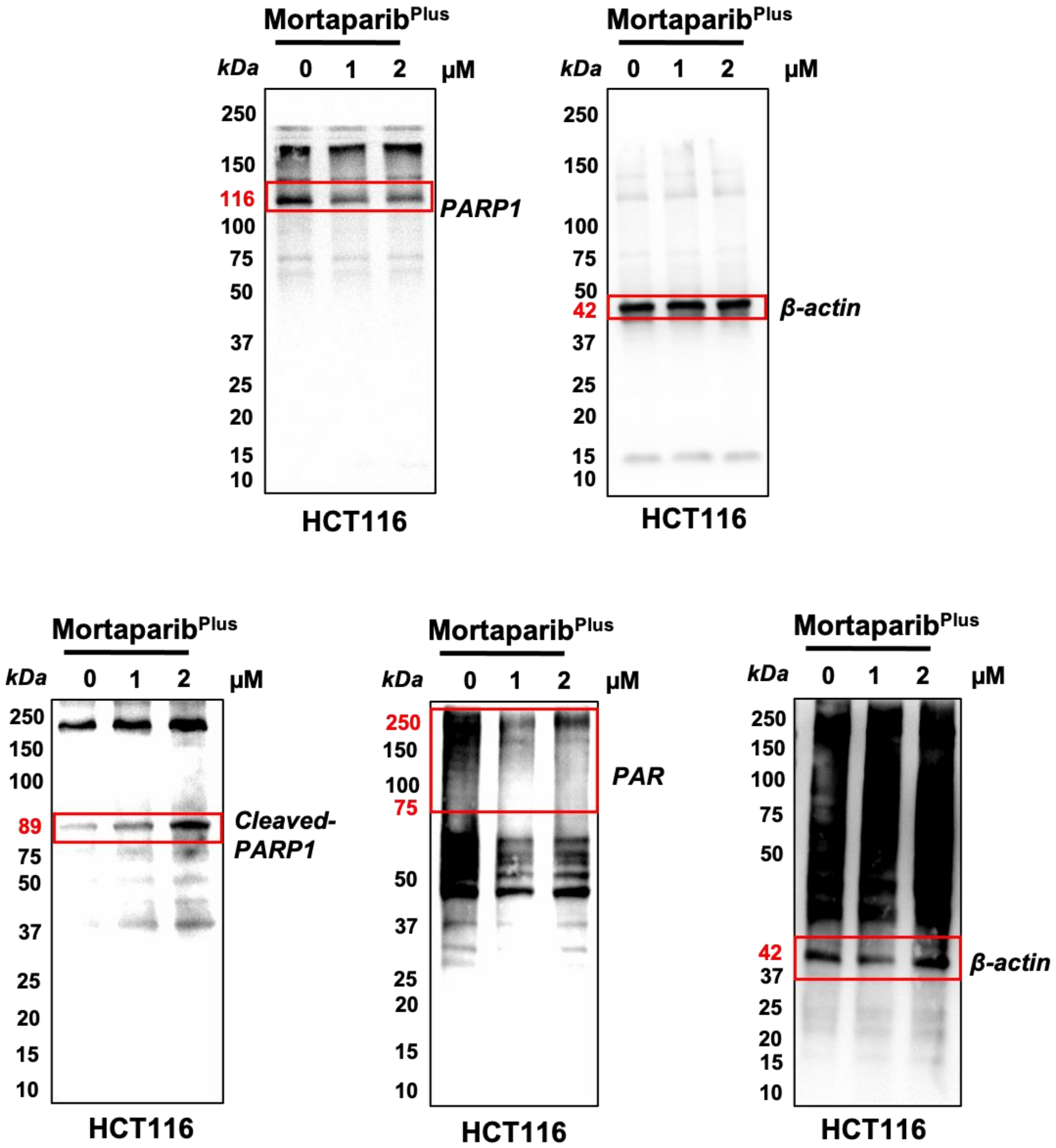

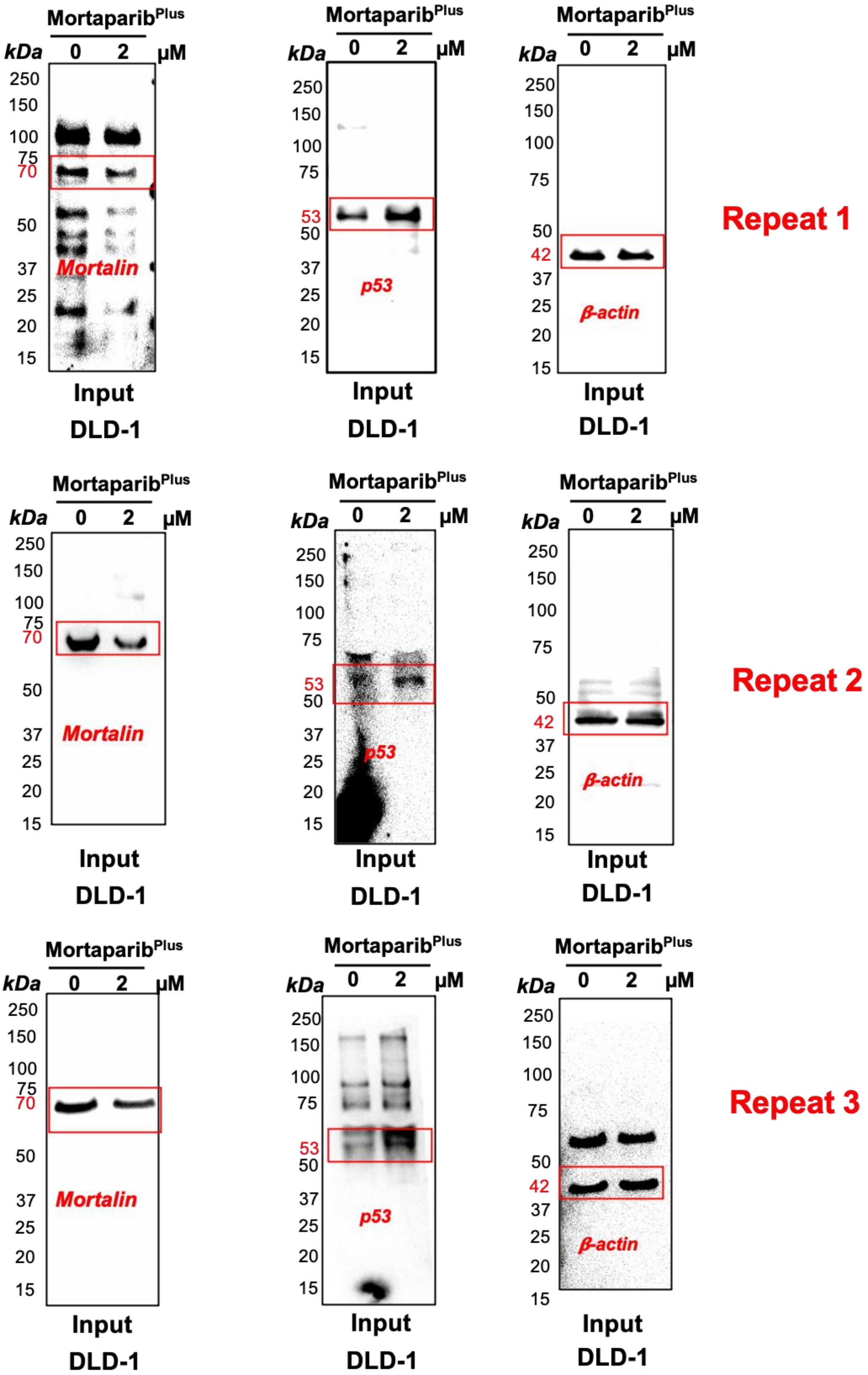
MortaparibPlus
kDa 0 2 µM
250
150
100
75
70
53
50
42
37
25
20
15
Mortalin
p53
β-actin
Input
DLD-1
Repeat 1
Repeat 2
Repeat 3

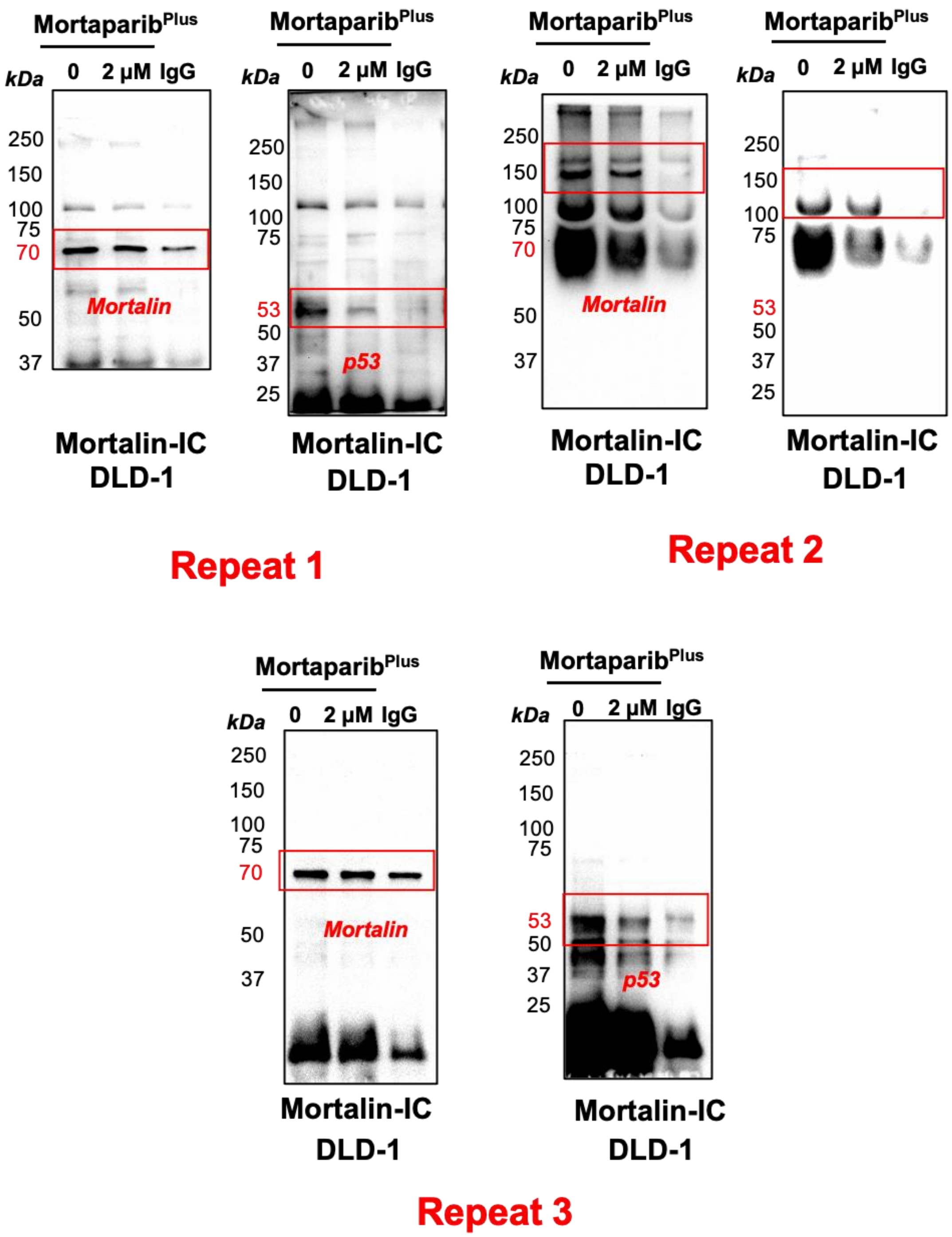

MortaparibPlus
kDa 0 2 µM IgG
250
150
100
75
70
Mortalin
50
37
Mortalin-IC
DLD-1
MortaparibPlus
kDa 0 2 µM IgG
250
150
100
75
53
50
37
25
p53
Mortalin-IC
DLD-1
Repeat 1
MortaparibPlus
kDa 0 2 µM IgG
250
150
100
75
70
Mortalin
50
37
Mortalin-IC
DLD-1
MortaparibPlus
kDa 0 2 µM IgG
250
150
100
75
53
50
37
25
Mortalin-IC
DLD-1
Repeat 2
MortaparibPlus
kDa 0 2 µM IgG
250
150
100
75
70
Mortalin
50
37
Mortalin-IC
DLD-1
MortaparibPlus
kDa 0 2 µM IgG
250
150
100
75
53
50
37
25
p53
Mortalin-IC
DLD-1
Repeat 3

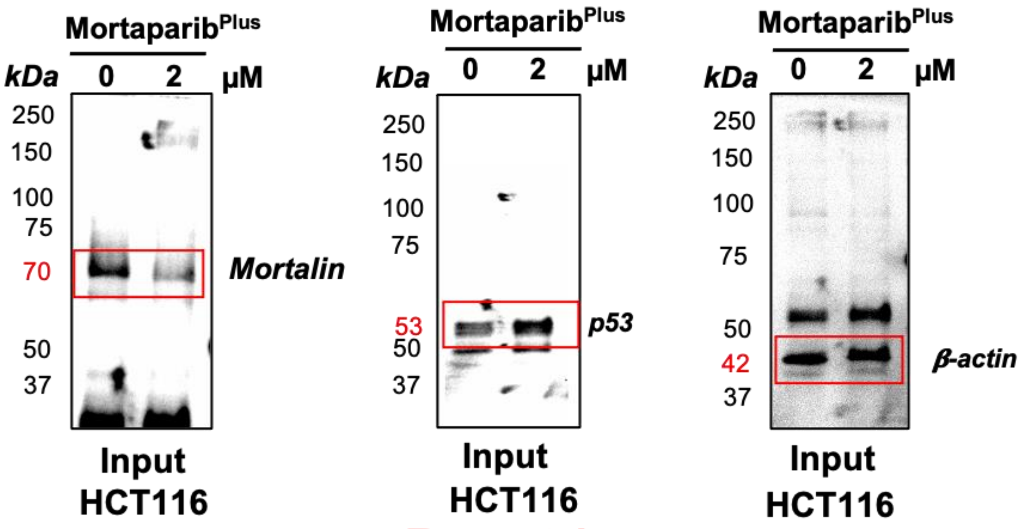

Repeat 1

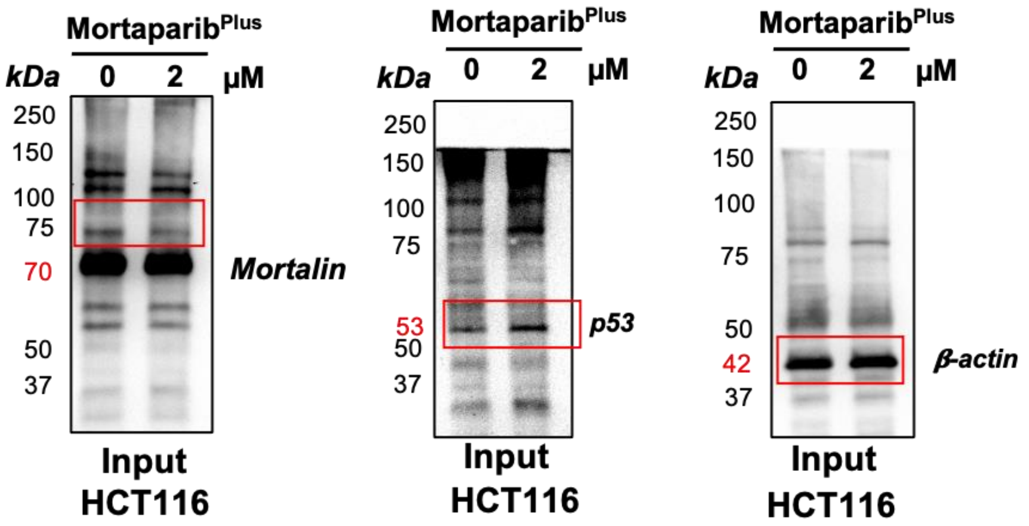

Repeat 2

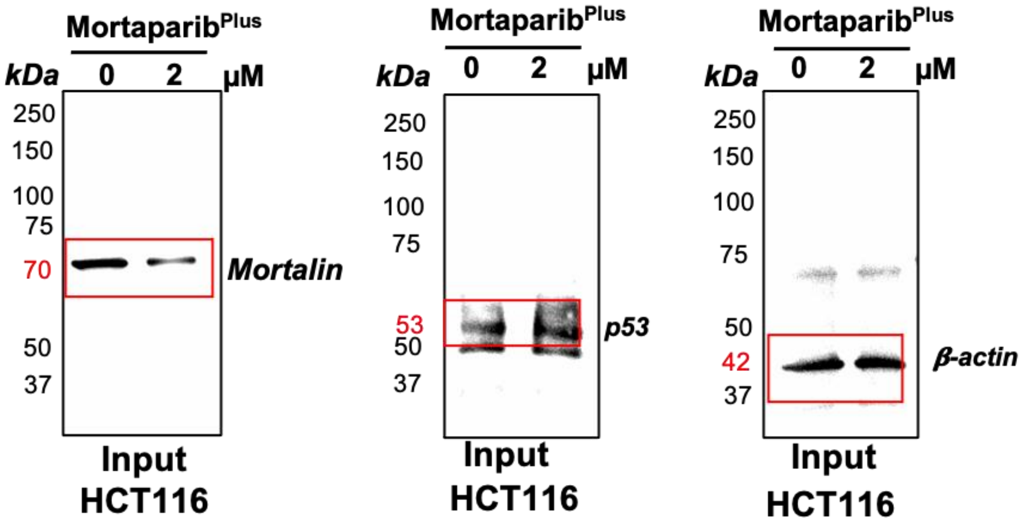

Repeat 3

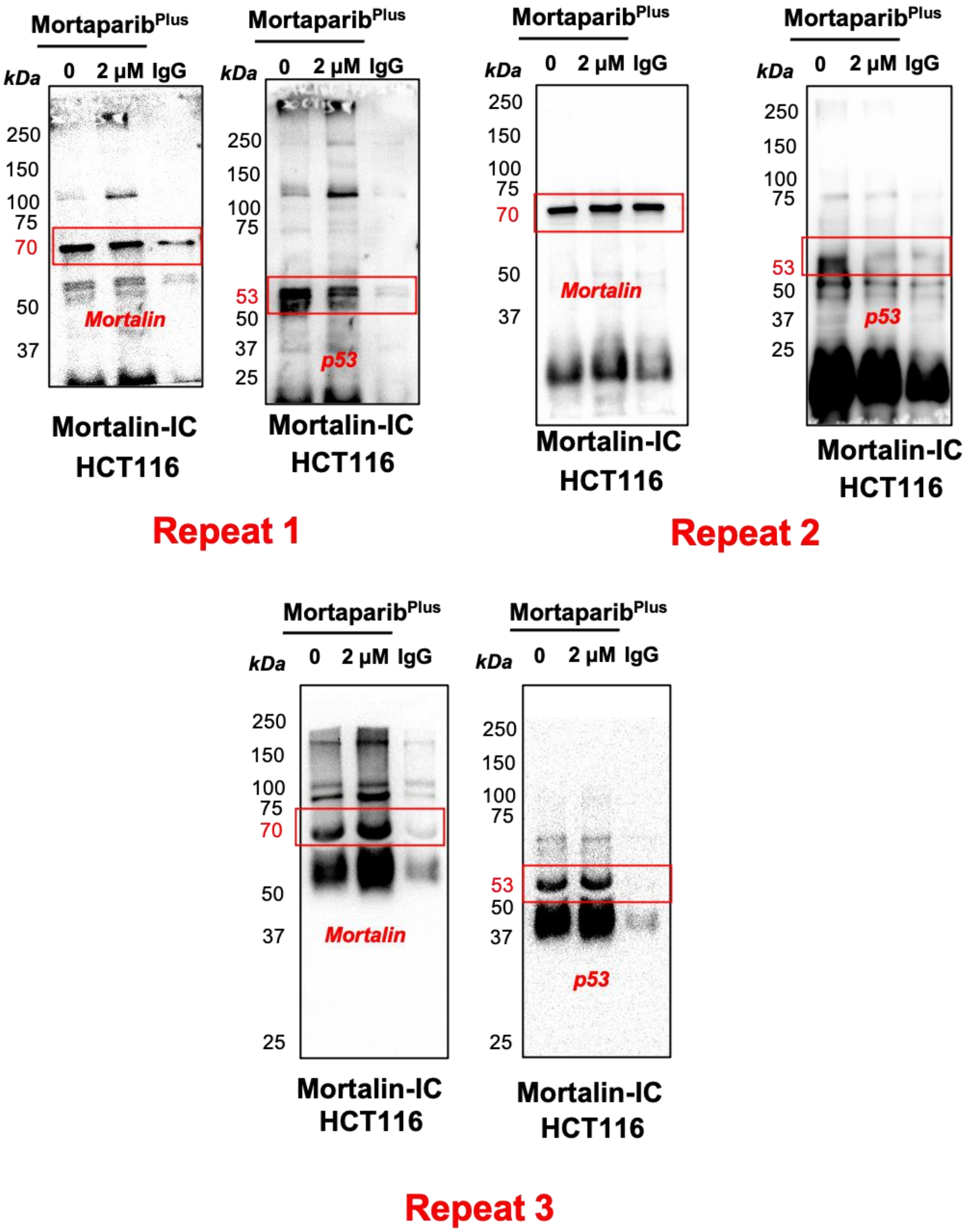

MortaparibPlus
kDa
0
2 μM
IgG
250
150
100
75
70
53
50
37
25
Mortalin
p53
Mortalin-IC
HCT116
Repeat 1
Repeat 2
Repeat 3

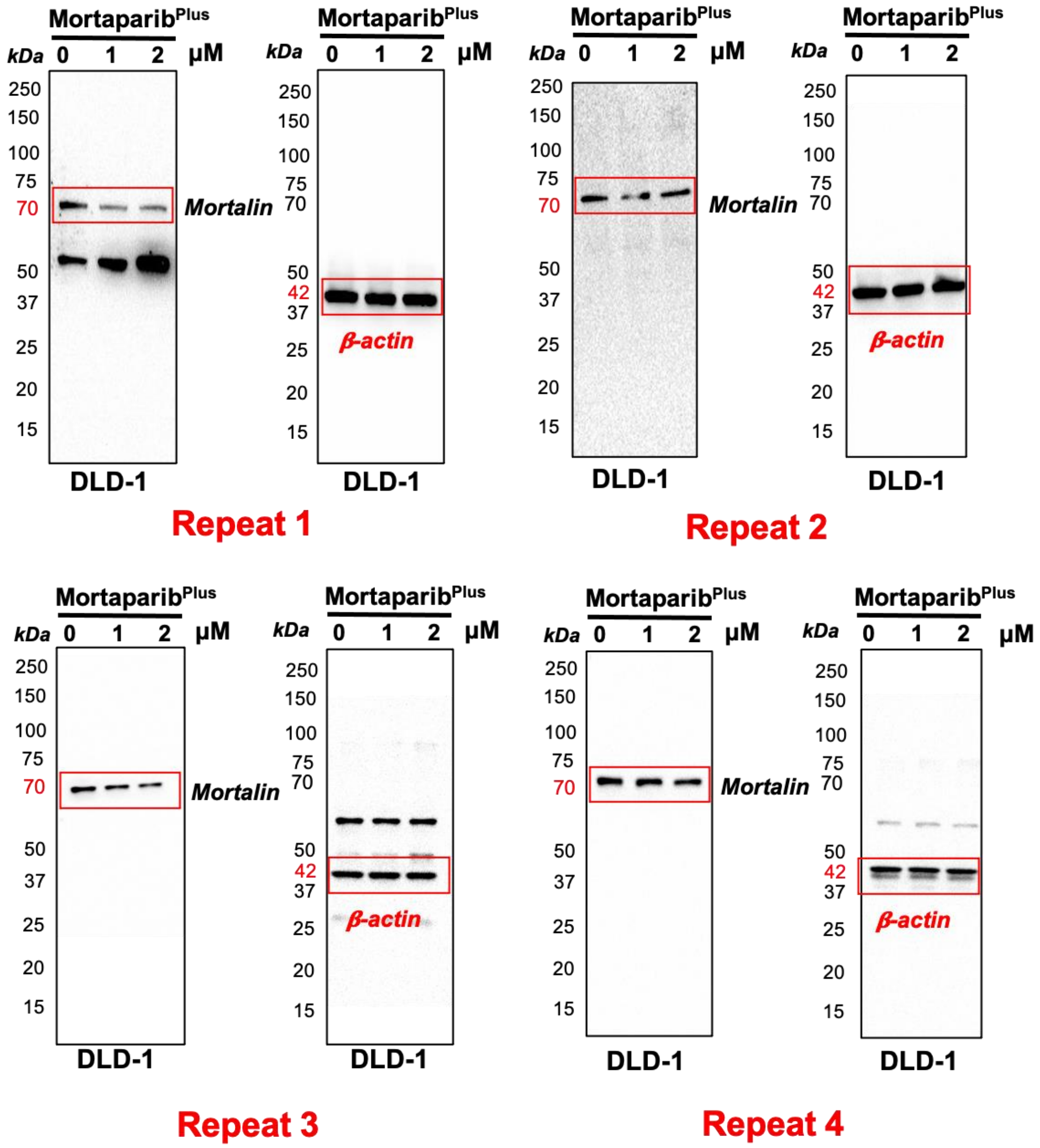
MortaparibPlus
kDa 0 1 2 µM
250 150 100 75 70 50 37 25 20 15
Mortalin
β-actin
42
DLD-1
Repeat 1
Repeat 2
Repeat 3
Repeat 4

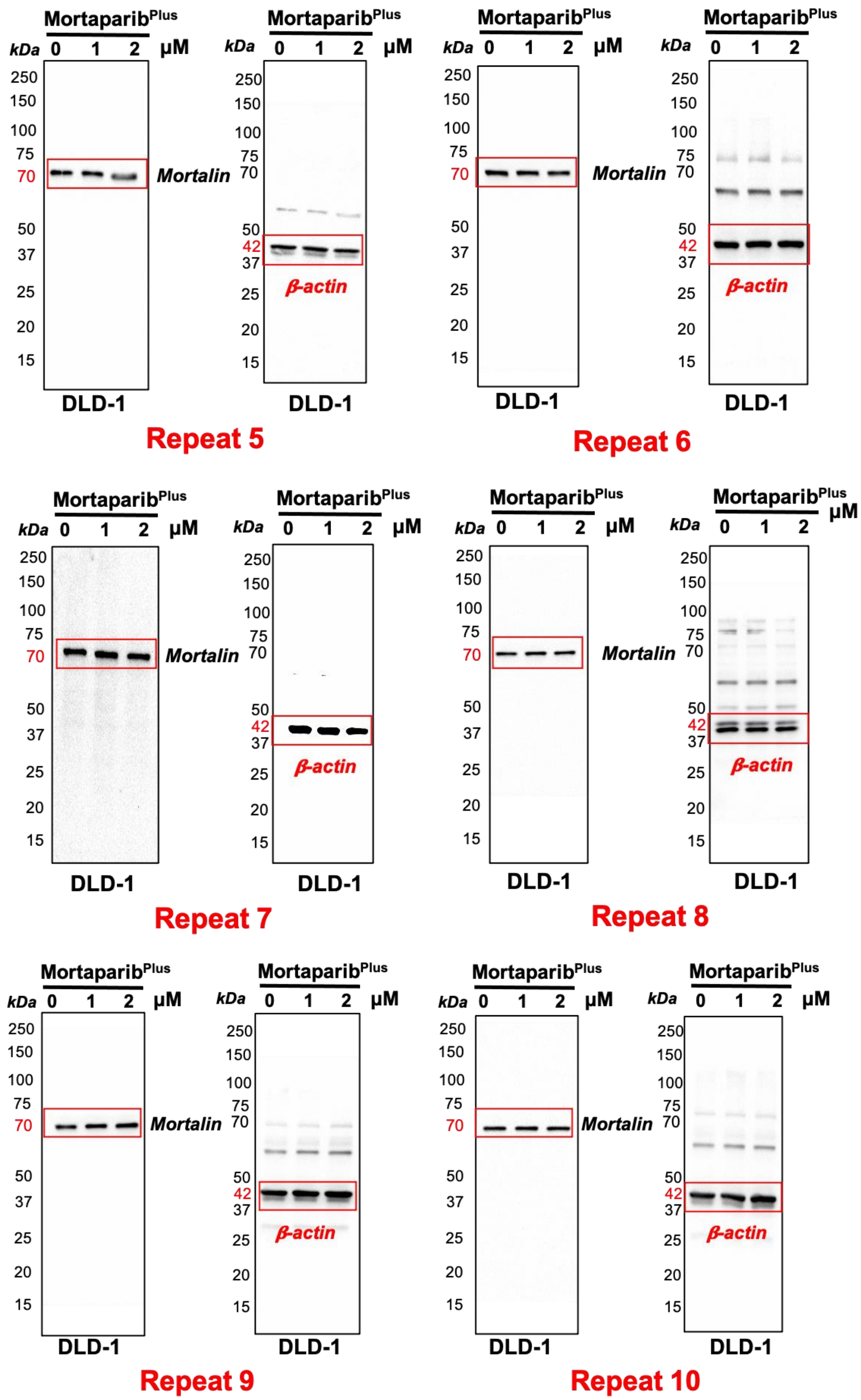
Mortaparib^Plus
kDa 0 1 2 μM
250
150
100
75
70
50
42
37
25
20
15
Mortalin
β-actin
DLD-1
Repeat 5
Repeat 6
Repeat 7
Repeat 8
Repeat 9
Repeat 10

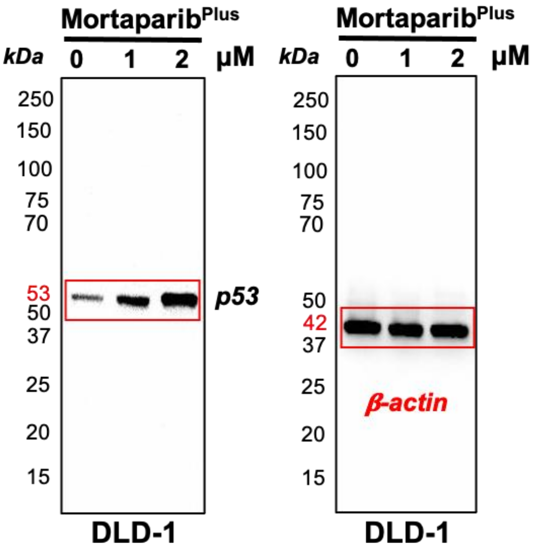

Repeat 1

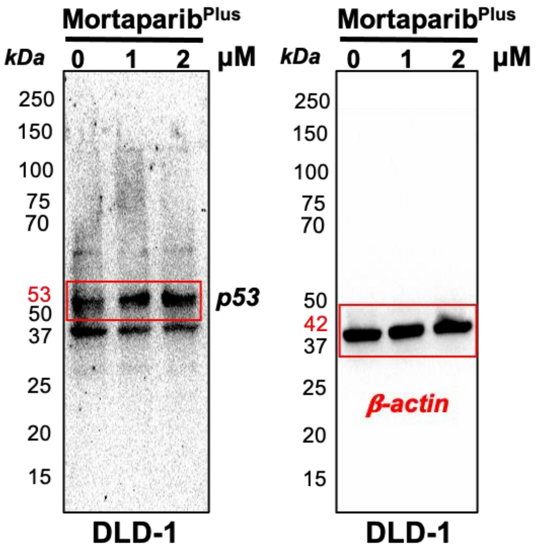

Repeat 2

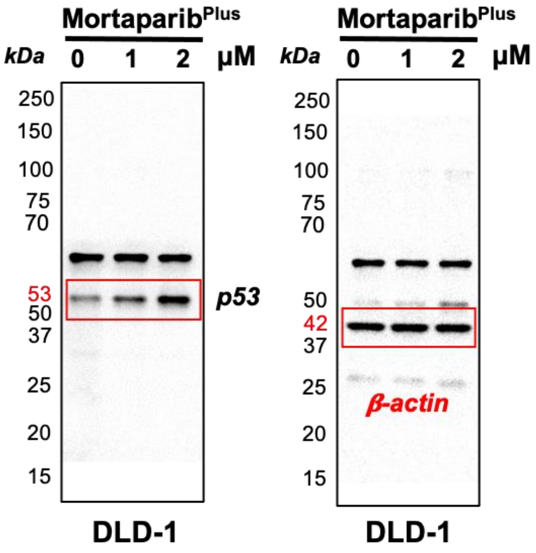

Repeat 3

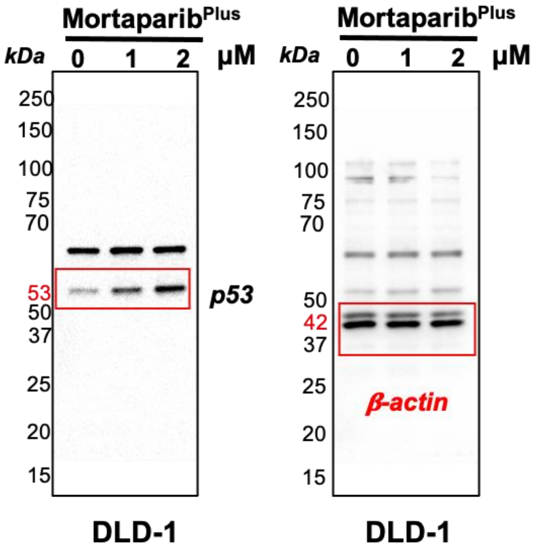

Repeat 4

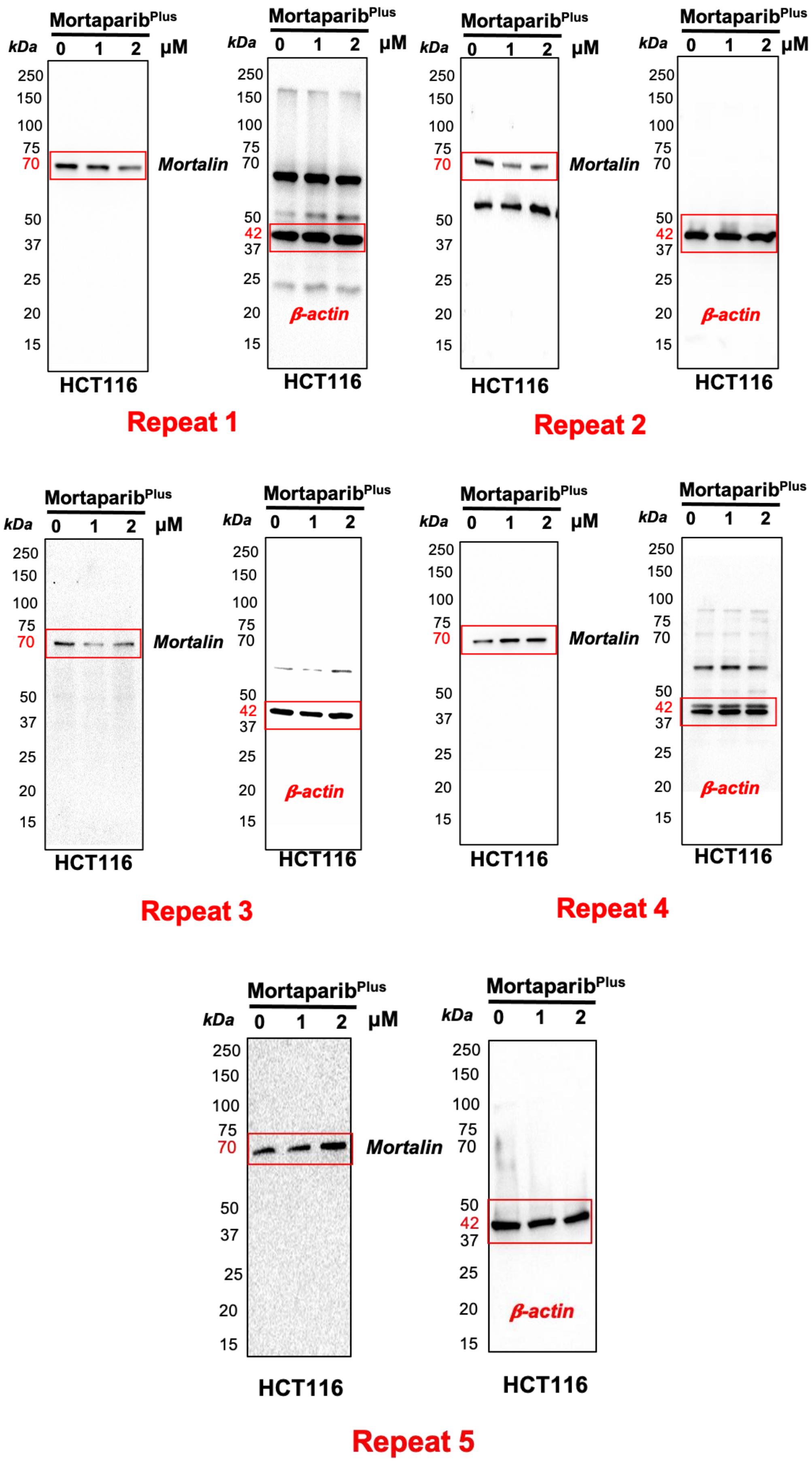
MortaparibPlus
kDa
0 1 2 μM
250
150
100
75
70
50
42
37
25
20
15
Mortalin
β-actin
HCT116
Repeat 1
Repeat 2
Repeat 3
Repeat 4
Repeat 5

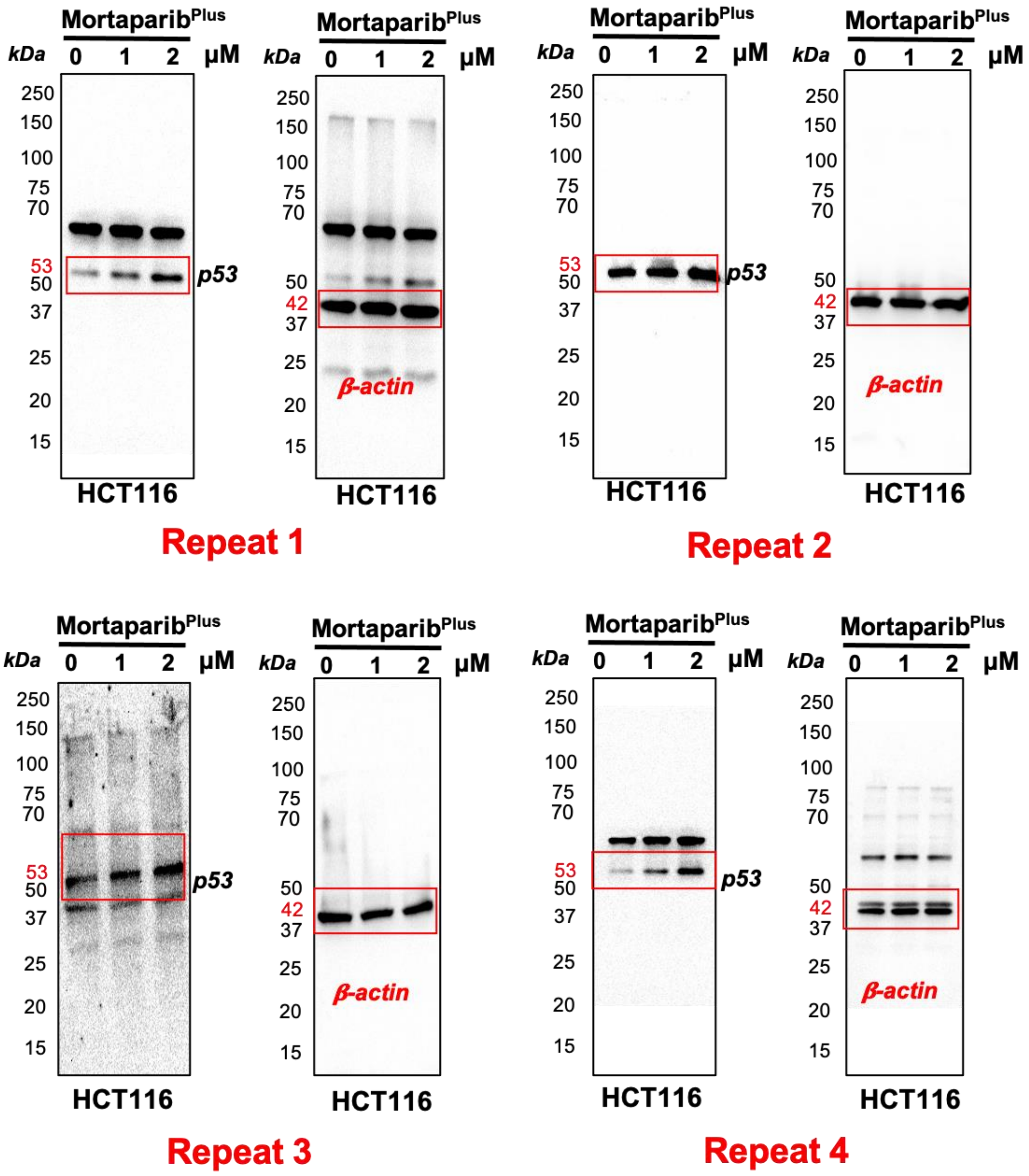
Mortaparib^Plus
kDa 0 1 2 μM
250
150
100
75
70
53
50
37
25
20
15
p53
β-actin
HCT116
Repeat 1
Repeat 2
Repeat 3
Repeat 4

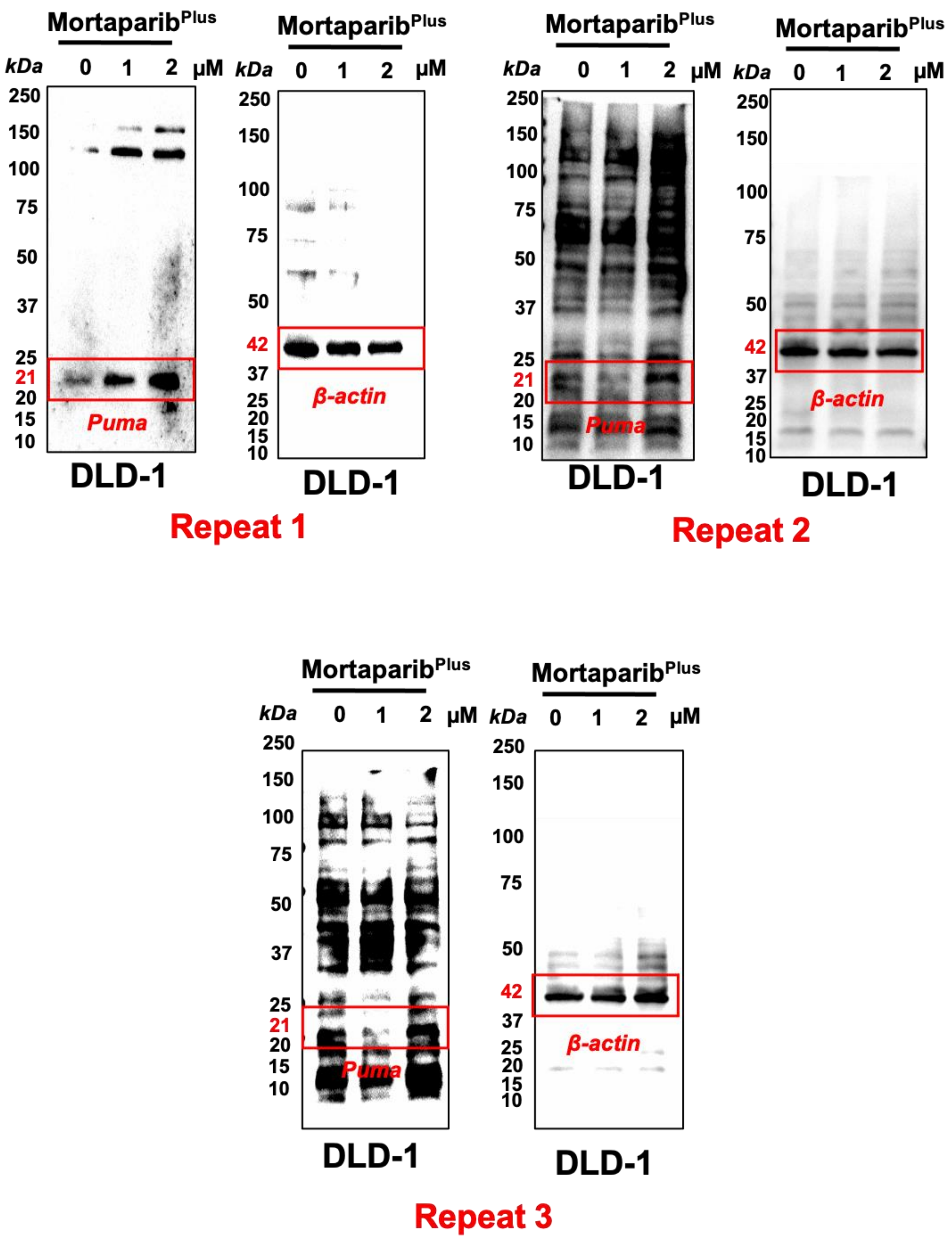

MortaparibPlus
kDa 0 1 2 μM
250
150
100
75
50
37
25
21
20
15
10
Puma
β-actin
42
DLD-1
Repeat 1
Repeat 2
Repeat 3

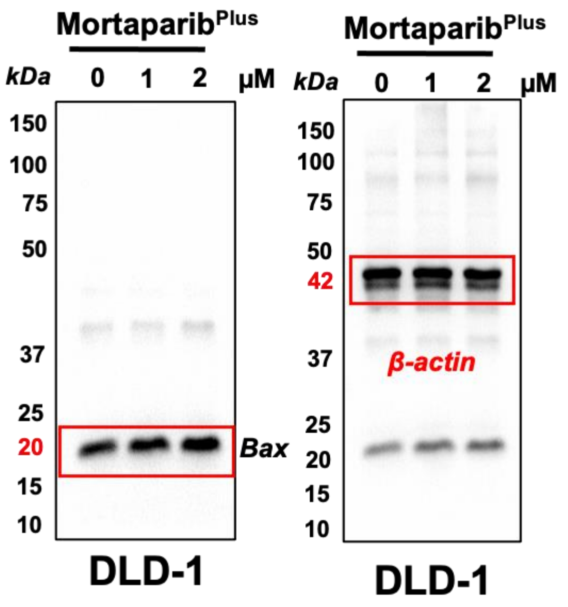

Repeat 1

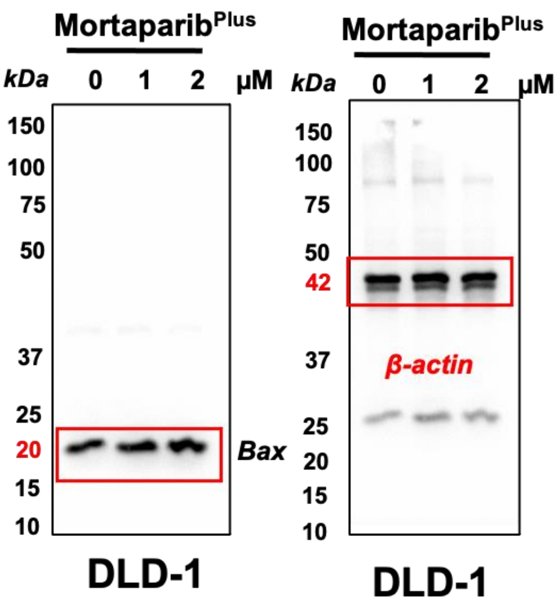

Repeat 2

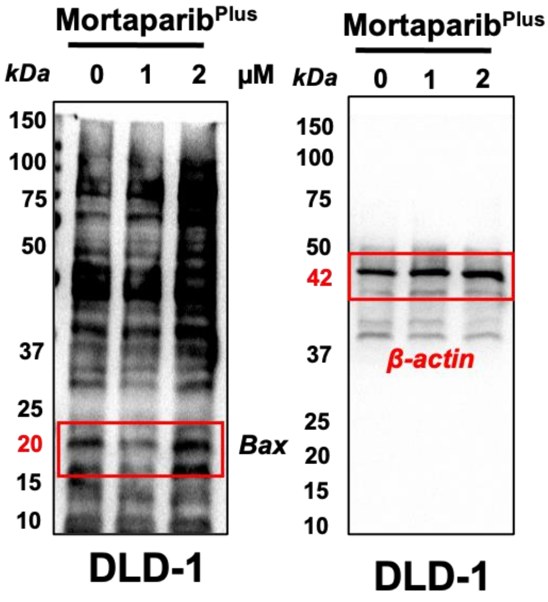

Repeat 3

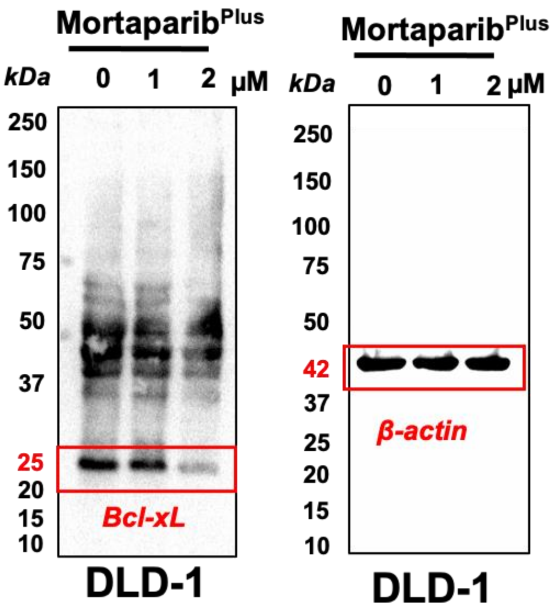

Repeat 1

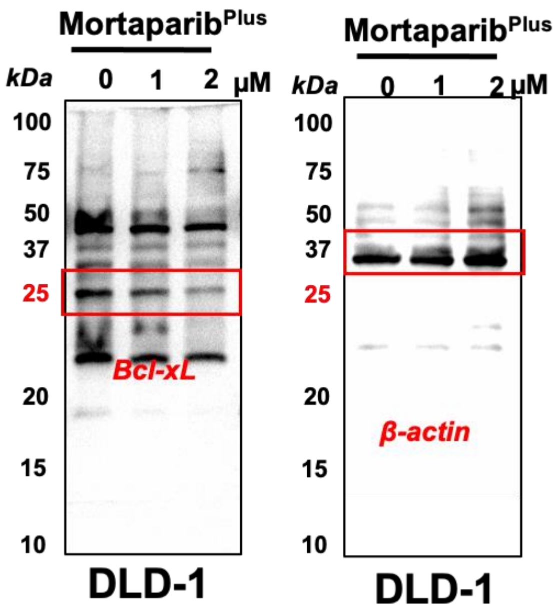

Repeat 2

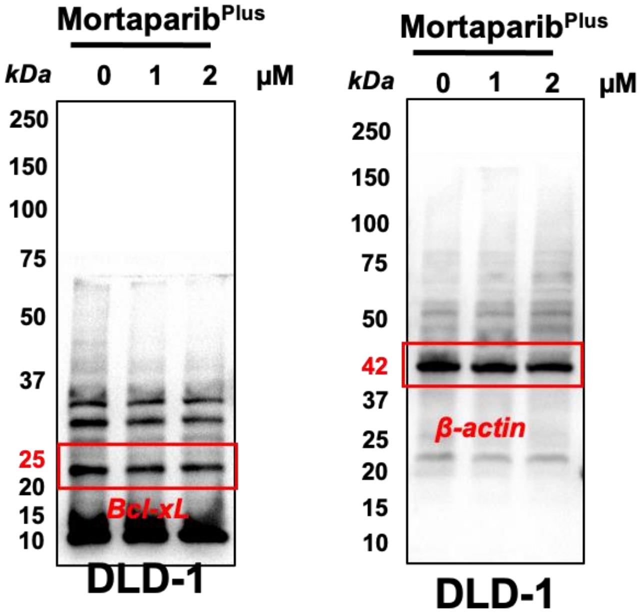

Repeat 3

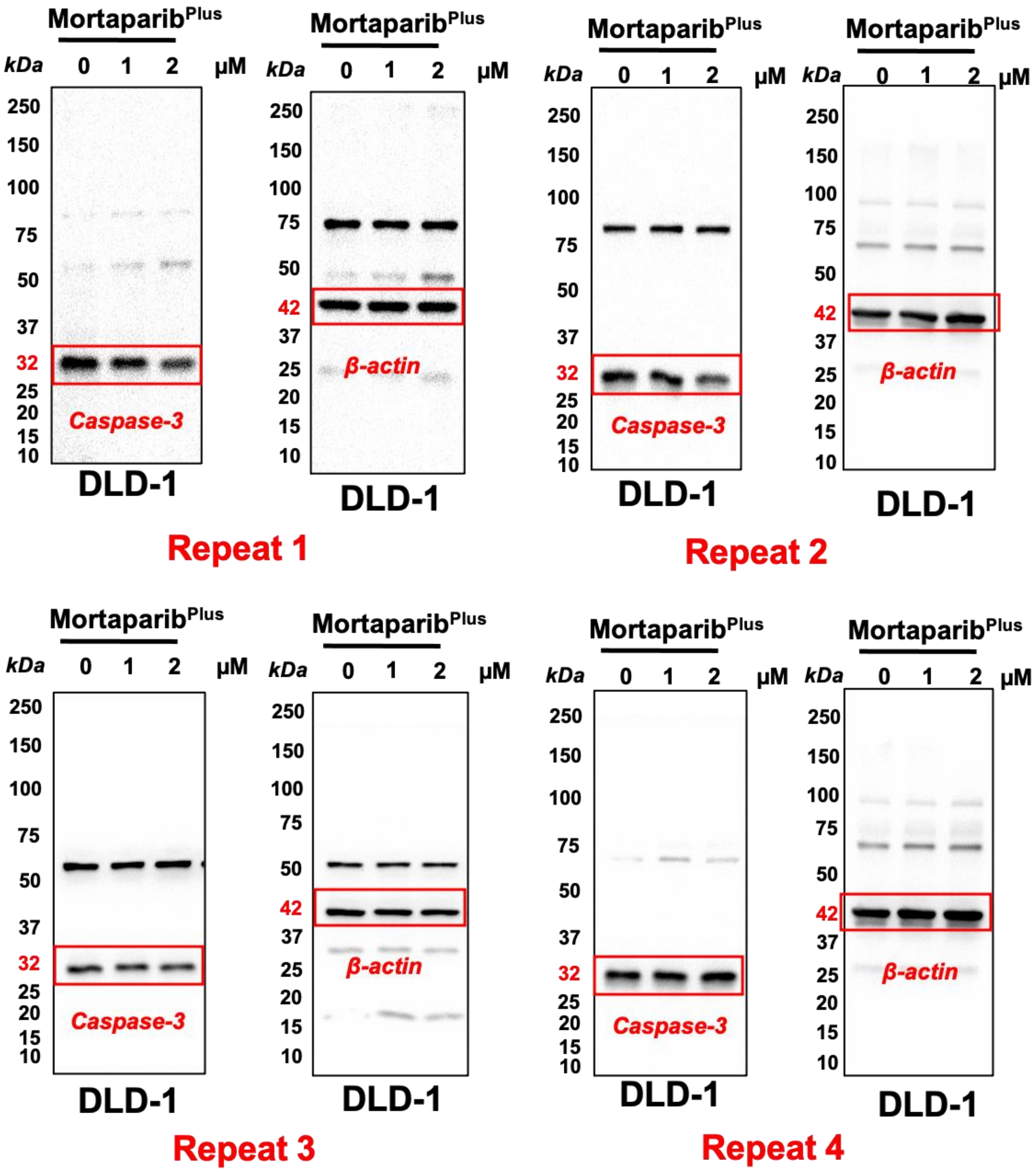
Mortaparib^Plus
kDa 0 1 2 μM
250
150
100
75
50
37
32
25
20
15
10
Caspase-3
DLD-1
42
β-actin
Repeat 1
Repeat 2
Repeat 3
Repeat 4

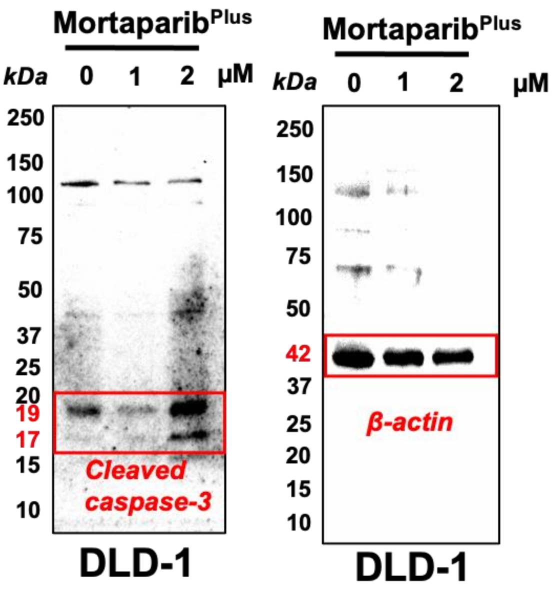

Repeat 1

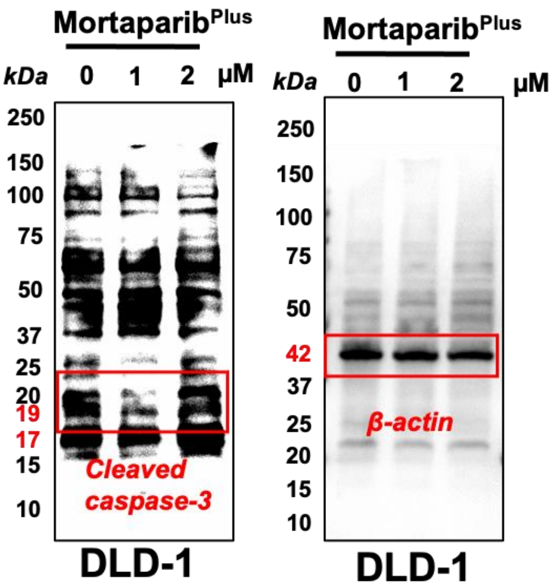

Repeat 2

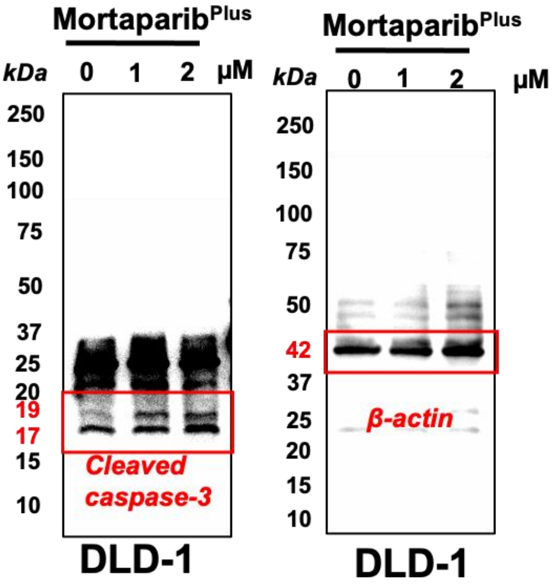

Repeat 3

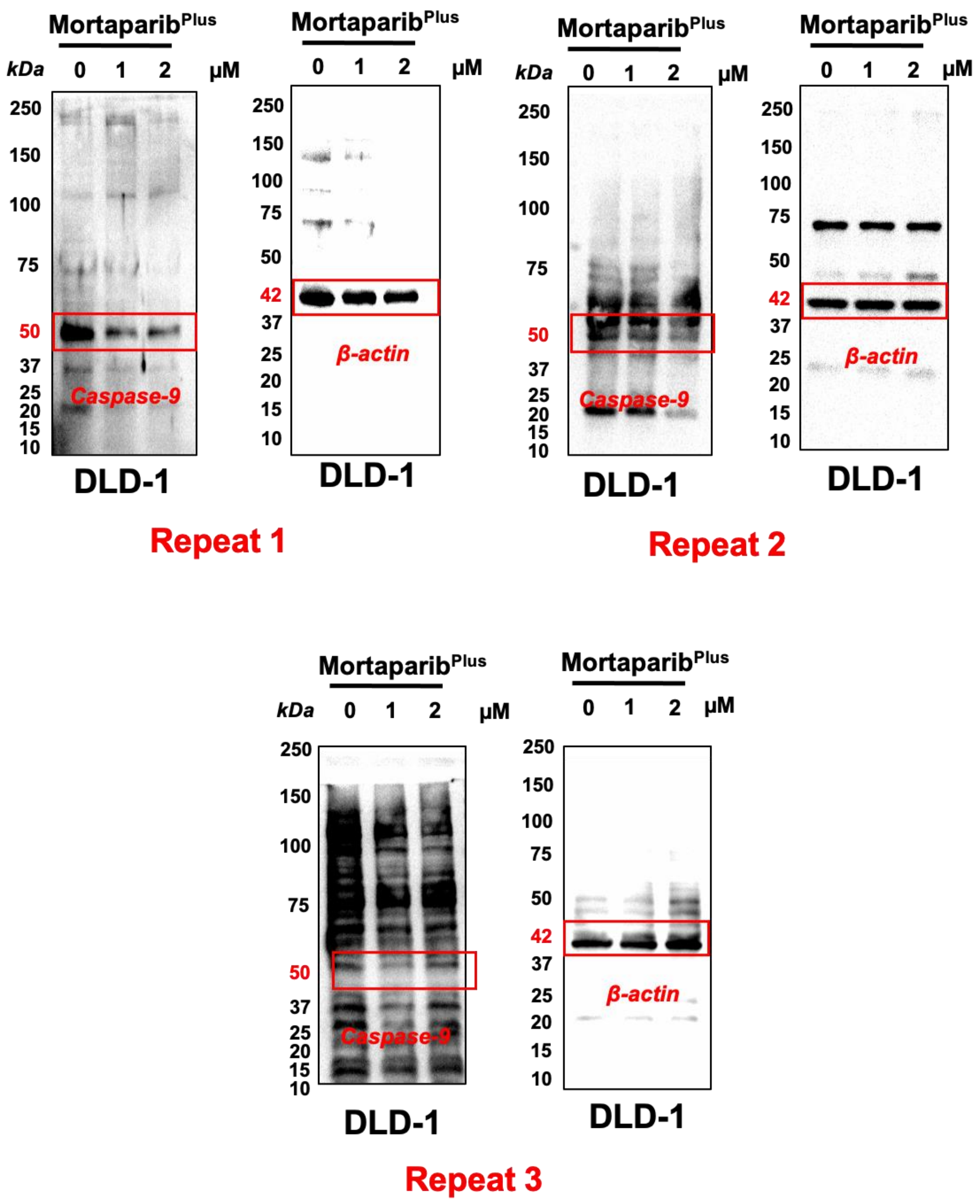

Mortaparib^Plus
kDa 0 1 2 μM
250
150
100
75
50
37
25
20
15
10
Caspase-9
DLD-1
Mortaparib^Plus
0 1 2 μM
250
150
100
75
50
42
37
25
20
15
10
β-actin
DLD-1
Repeat 1
Mortaparib^Plus
kDa 0 1 2 μM
250
150
100
75
50
37
25
20
15
10
Caspase-9
DLD-1
Mortaparib^Plus
0 1 2 μM
250
150
100
75
50
42
37
25
20
15
10
β-actin
DLD-1
Repeat 2
Mortaparib^Plus
kDa 0 1 2 μM
250
150
100
75
50
37
25
20
15
10
Caspase-9
DLD-1
Mortaparib^Plus
0 1 2 μM
250
150
100
75
50
42
37
25
20
15
10
β-actin
DLD-1
Repeat 3

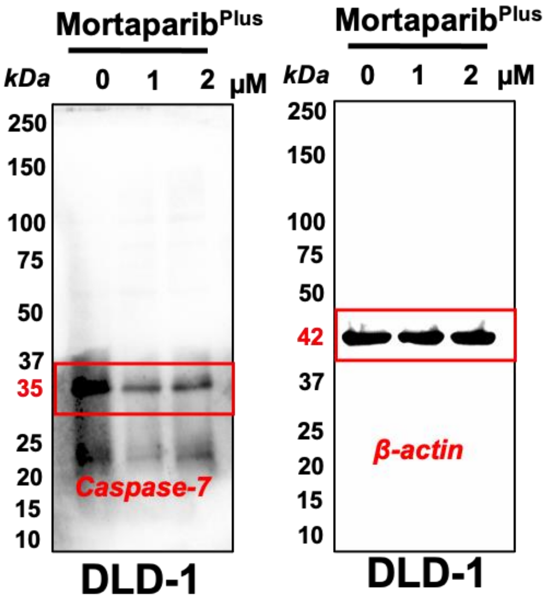

Repeat 1

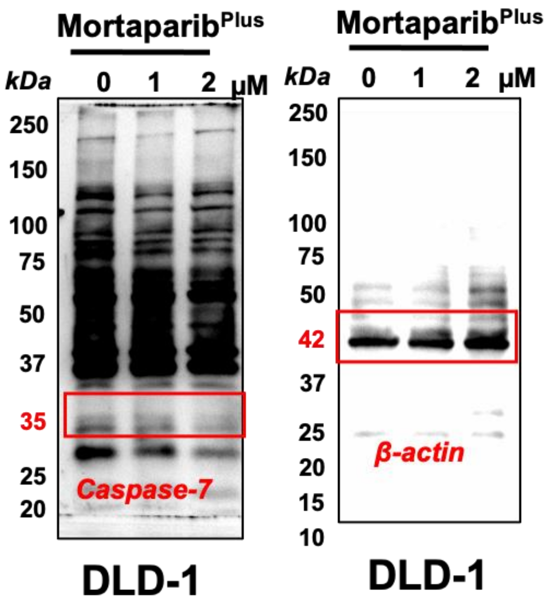

Repeat 2

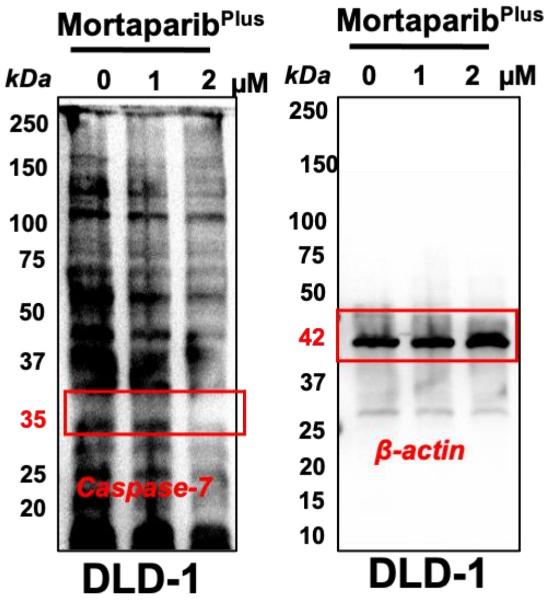

Repeat 3

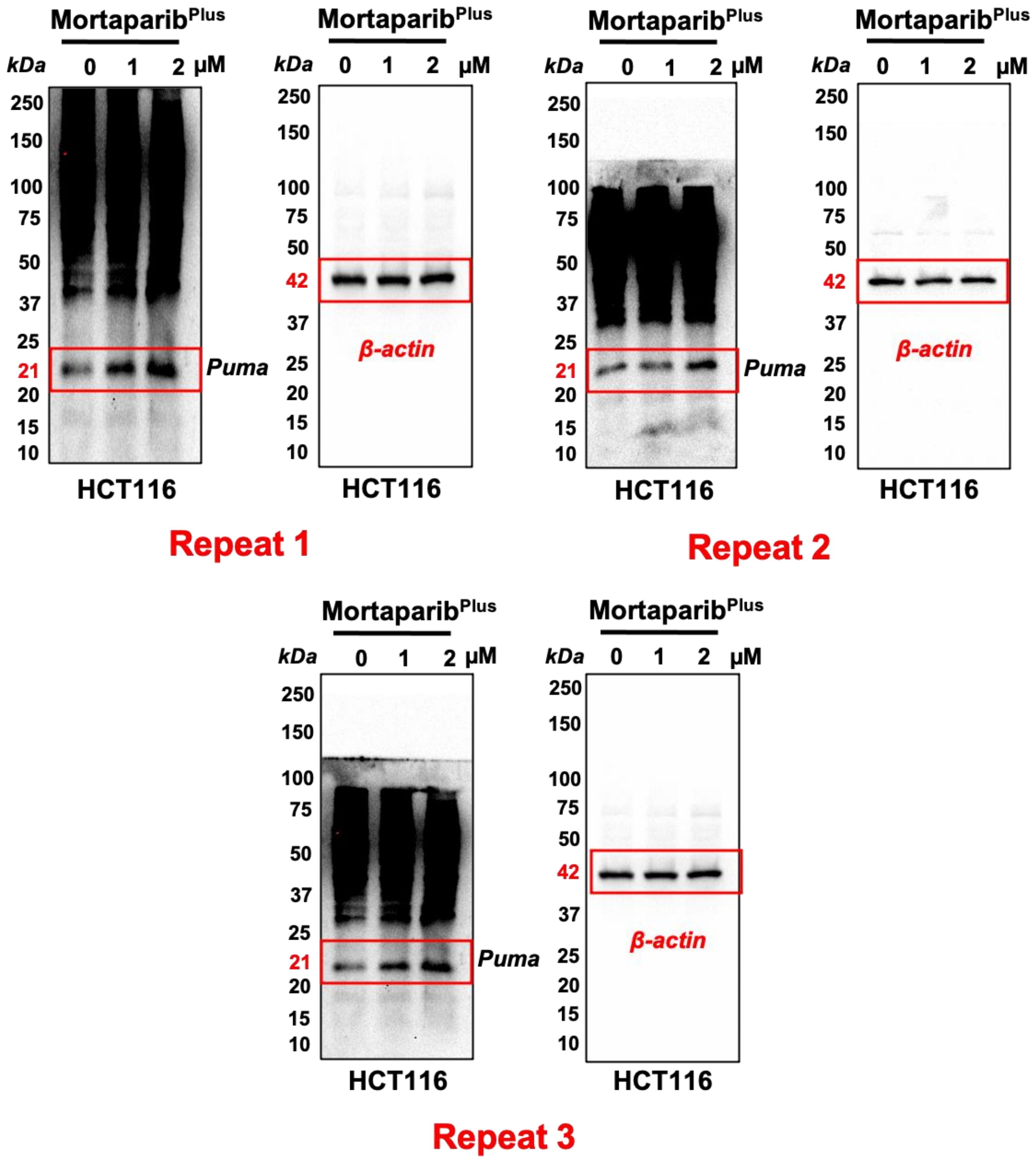
MortaparibPlus
kDa 0 1 2 μM
250 150 100 75 50 37 25 21 20 15 10
Puma
HCT116
MortaparibPlus
kDa 0 1 2 μM
250 150 100 75 50 42 37 25 20 15 10
β-actin
HCT116
Repeat 1
MortaparibPlus
kDa 0 1 2 μM
250 150 100 75 50 37 25 21 20 15 10
Puma
HCT116
MortaparibPlus
kDa 0 1 2 μM
250 150 100 75 50 42 37 25 20 15 10
β-actin
HCT116
Repeat 2
MortaparibPlus
kDa 0 1 2 μM
250 150 100 75 50 37 25 21 20 15 10
Puma
HCT116
MortaparibPlus
kDa 0 1 2 μM
250 150 100 75 50 42 37 25 20 15 10
β-actin
HCT116
Repeat 3

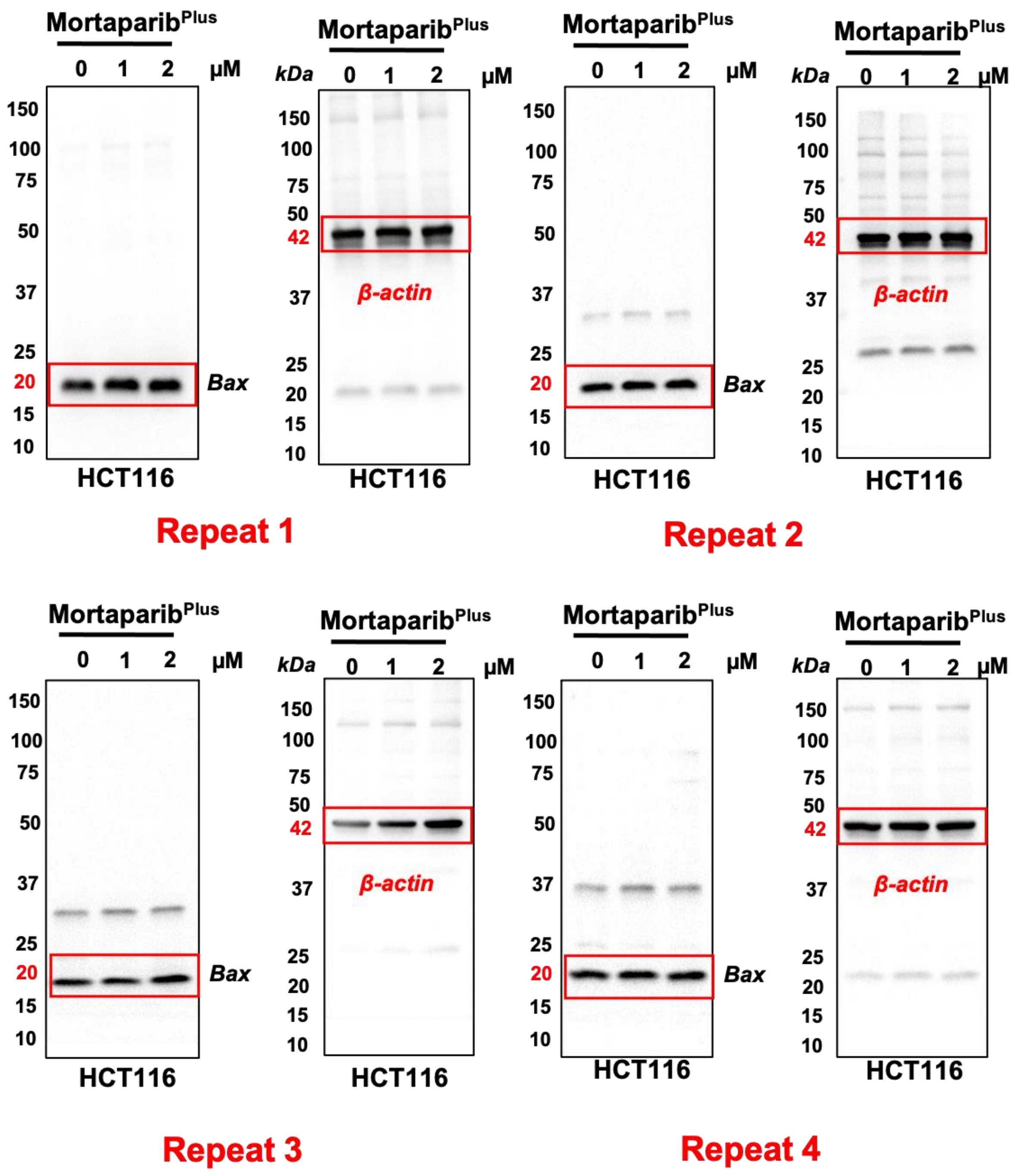
MortaparibPlus
0 1 2 μM
kDa
150
100
75
50
42
37
25
20
15
10
Bax
β-actin
HCT116
Repeat 1
Repeat 2
Repeat 3
Repeat 4

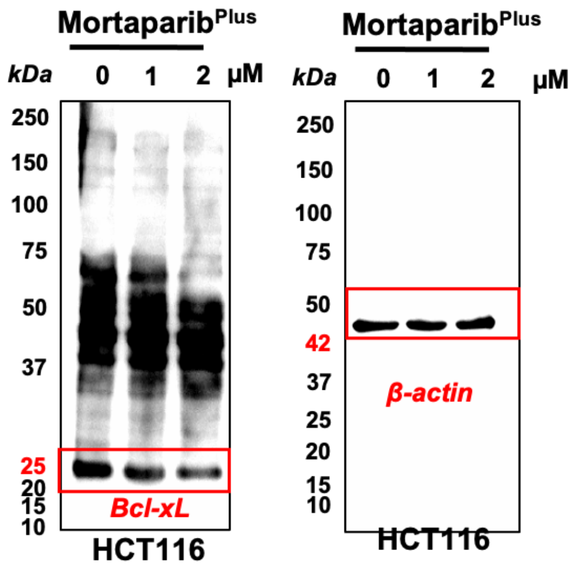

Repeat 1

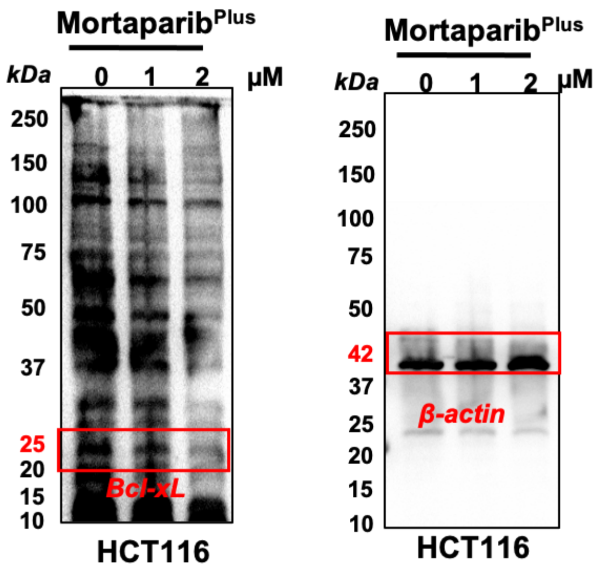

Repeat 2

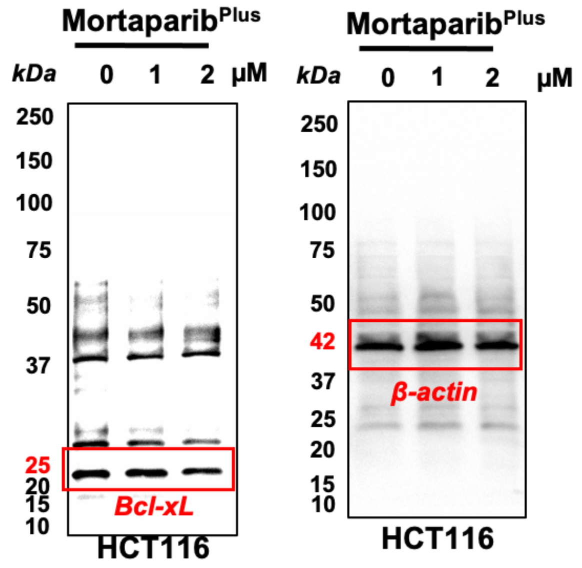

Repeat 3

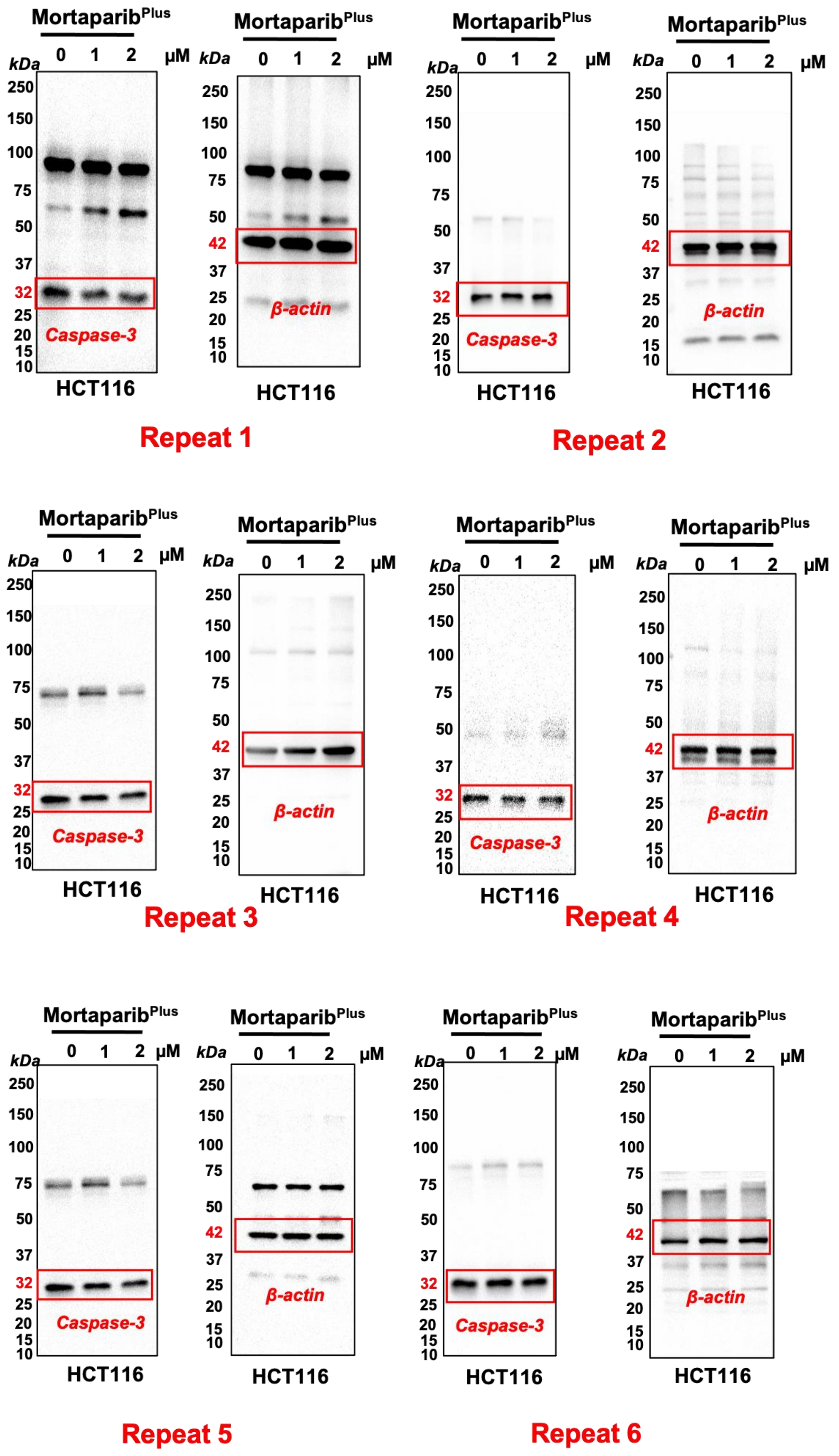

Mortaparib^Plus
kDa 0 1 2 μM
250 150 100 75 50 37 32 25 20 15 10
Caspase-3
HCT116
Mortaparib^Plus
kDa 0 1 2 μM
250 150 100 75 50 42 37 25 20 15 10
β-actin
HCT116
Repeat 1
Mortaparib^Plus
kDa 0 1 2 μM
250 150 100 75 50 37 32 25 20 15 10
Caspase-3
HCT116
Mortaparib^Plus
kDa 0 1 2 μM
250 150 100 75 50 42 37 25 20 15 10
β-actin
HCT116
Repeat 2
Mortaparib^Plus
kDa 0 1 2 μM
250 150 100 75 50 37 32 25 20 15 10
Caspase-3
HCT116
Mortaparib^Plus
kDa 0 1 2 μM
250 150 100 75 50 42 37 25 20 15 10
β-actin
HCT116
Repeat 3
Mortaparib^Plus
kDa 0 1 2 μM
250 150 100 75 50 37 32 25 20 15 10
Caspase-3
HCT116
Mortaparib^Plus
kDa 0 1 2 μM
250 150 100 75 50 42 37 25 20 15 10
β-actin
HCT116
Repeat 4
Mortaparib^Plus
kDa 0 1 2 μM
250 150 100 75 50 37 32 25 20 15 10
Caspase-3
HCT116
Mortaparib^Plus
kDa 0 1 2 μM
250 150 100 75 50 42 37 25 20 15 10
β-actin
HCT116
Repeat 5
Mortaparib^Plus
kDa 0 1 2 μM
250 150 100 75 50 37 32 25 20 15 10
Caspase-3
HCT116
Mortaparib^Plus
kDa 0 1 2 μM
250 150 100 75 50 42 37 25 20 15 10
β-actin
HCT116
Repeat 6

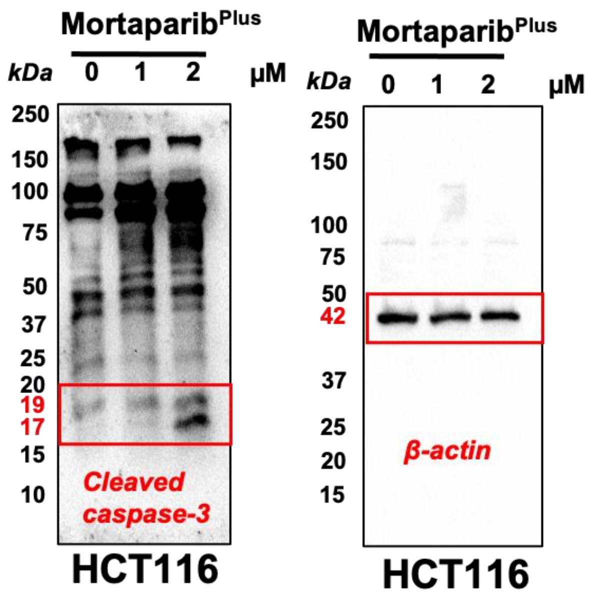

Repeat 1

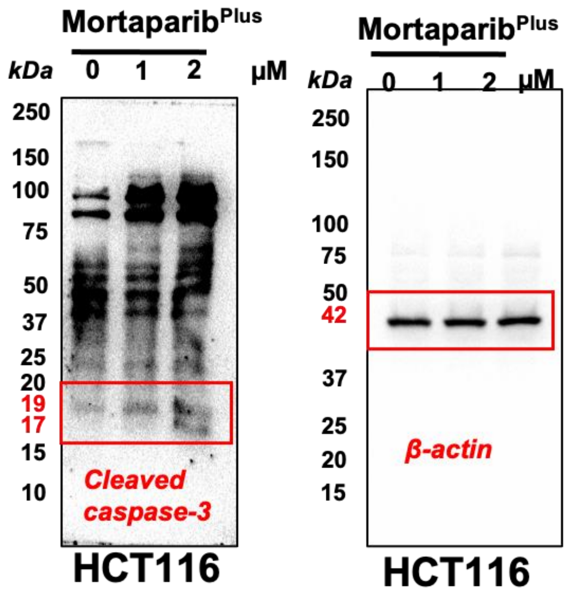

Repeat 2

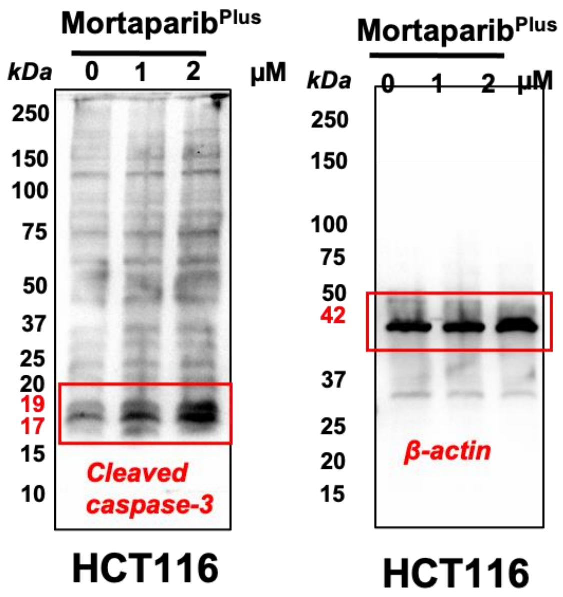

Repeat 3

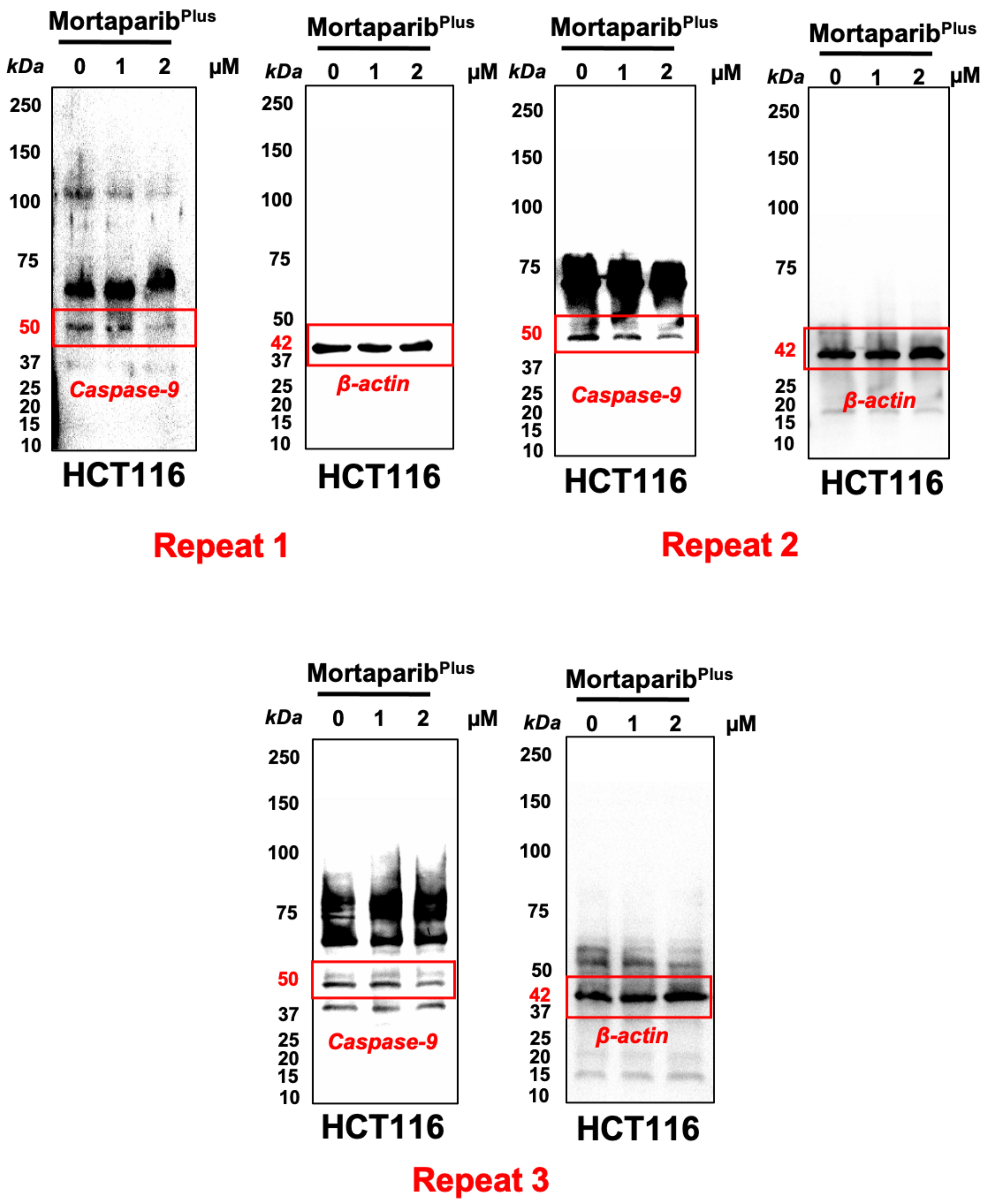

Mortaparib^Plus
kDa 0 1 2 μM
250
150
100
75
50
37
25
20
15
10
Caspase-9
HCT116
Mortaparib^Plus
kDa 0 1 2 μM
250
150
100
75
50
42
37
25
20
15
10
β-actin
HCT116
Repeat 1
Mortaparib^Plus
kDa 0 1 2 μM
250
150
100
75
50
37
25
20
15
10
Caspase-9
HCT116
Mortaparib^Plus
kDa 0 1 2 μM
250
150
100
75
42
25
20
15
10
β-actin
HCT116
Repeat 2
Mortaparib^Plus
kDa 0 1 2 μM
250
150
100
75
50
37
25
20
15
10
Caspase-9
HCT116
Mortaparib^Plus
kDa 0 1 2 μM
250
150
100
75
50
42
37
25
20
15
10
β-actin
HCT116
Repeat 3

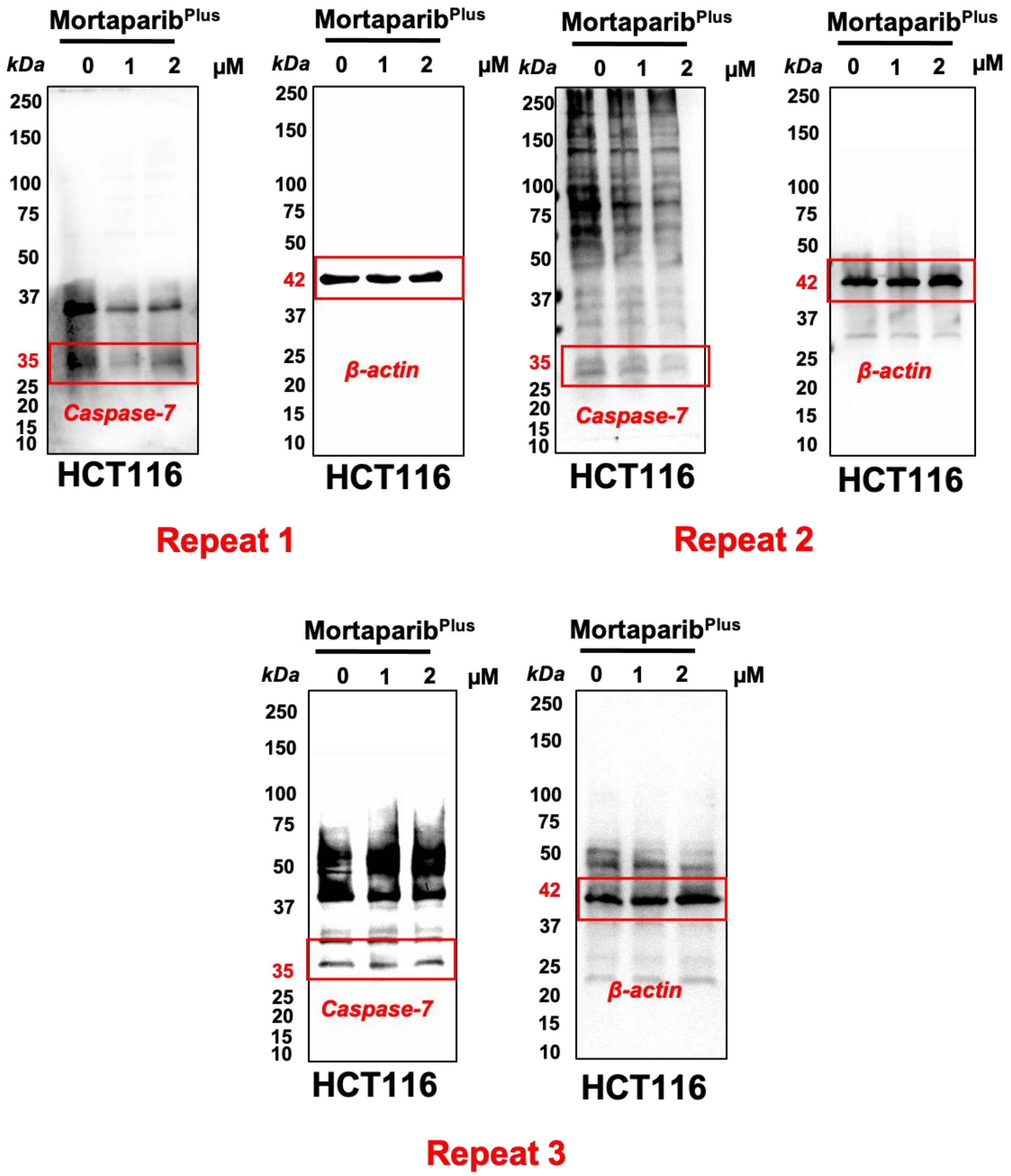

MortaparibPlus
kDa 0 1 2 μM
250
150
100
75
50
37
35
25
20
15
10
Caspase-7
42
β-actin
HCT116
Repeat 1
Repeat 2
Repeat 3

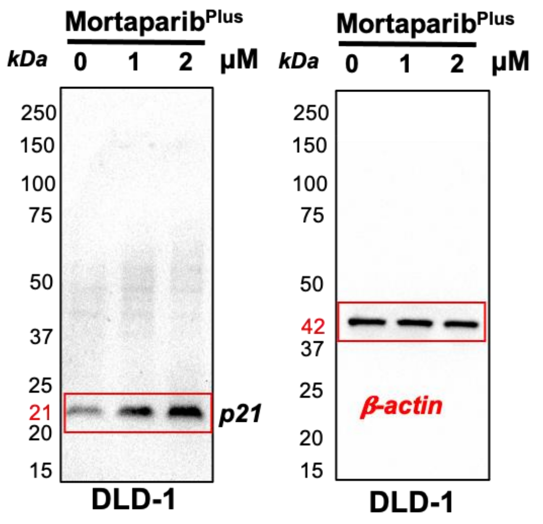

Repeat 1

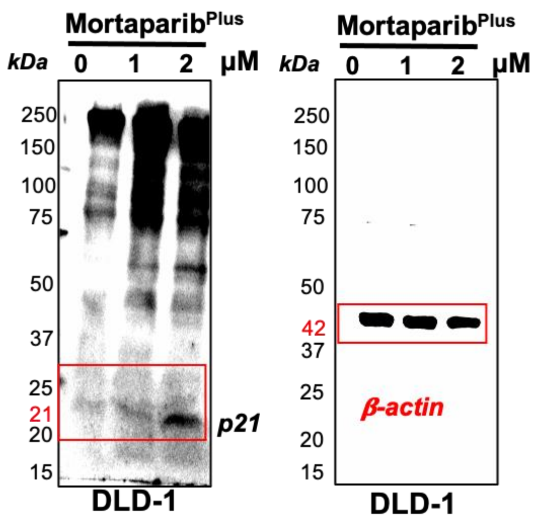

Repeat 2

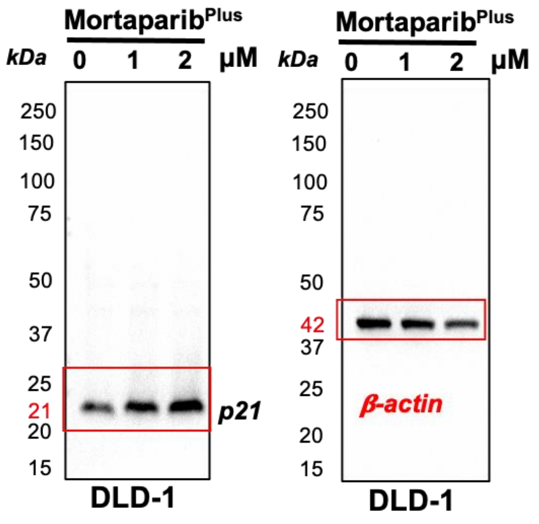

Repeat 3

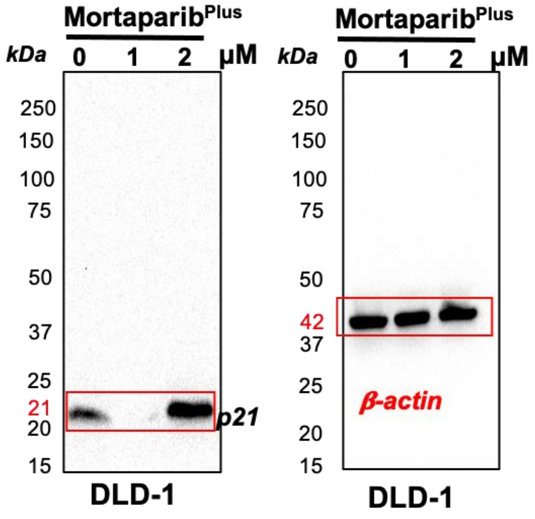

Repeat 4

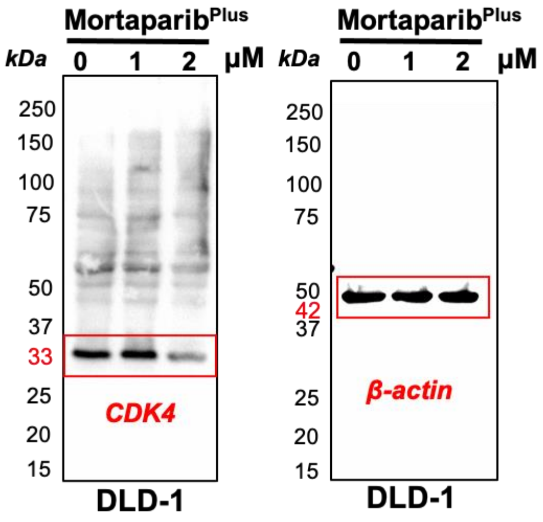

Repeat 1

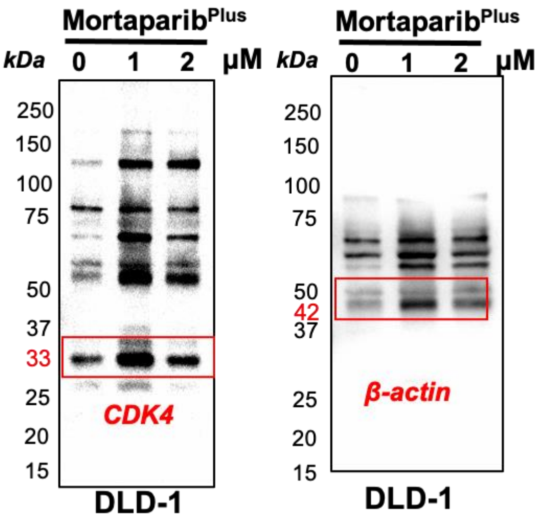

Repeat 2

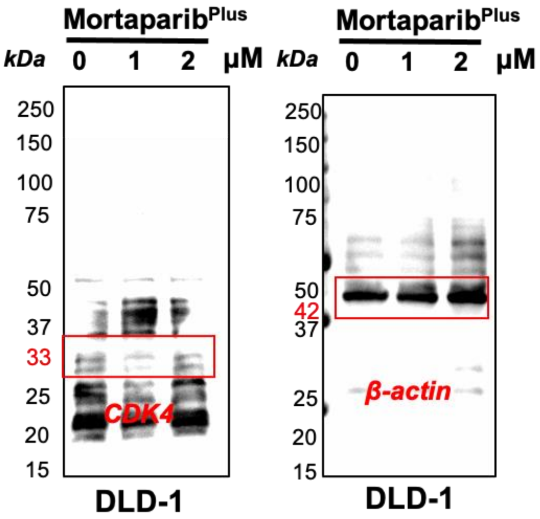

Repeat 3

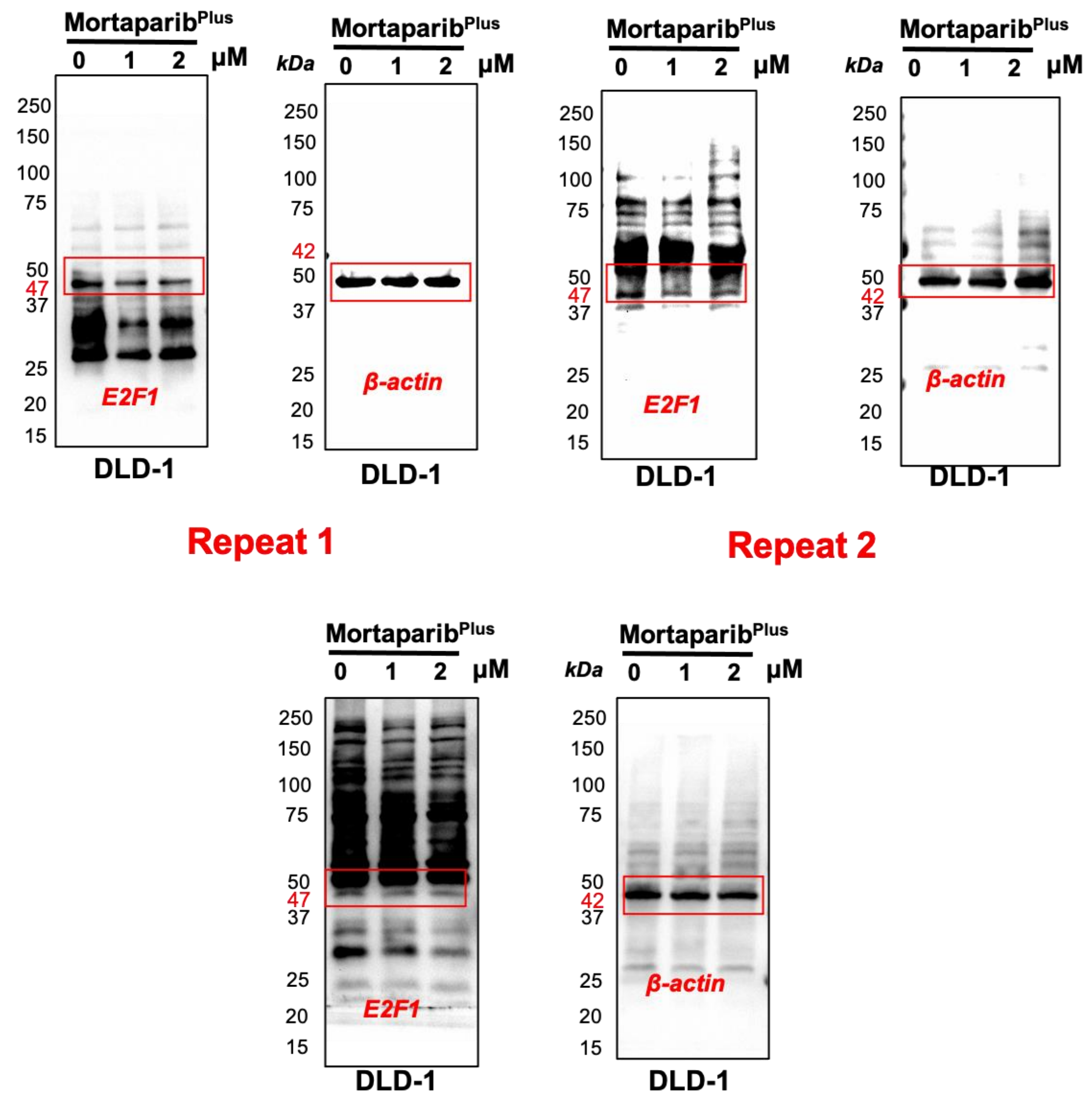
Mortaparib^Plus
0 1 2 μM
kDa
250
150
100
75
50
47
42
37
25
20
15
E2F1
β-actin
DLD-1
Repeat 1
Repeat 2

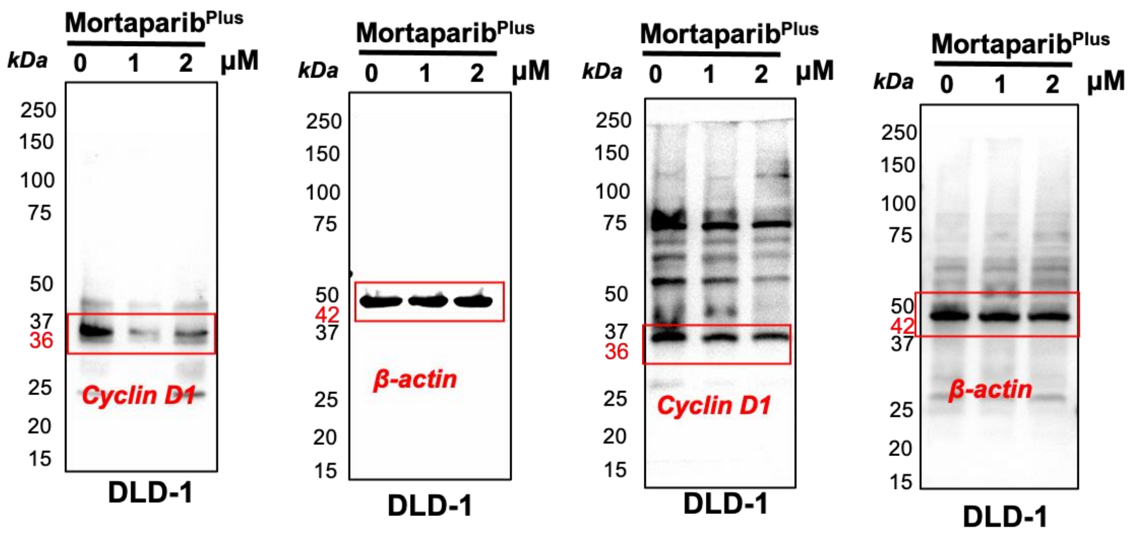

Repeat 1

Repeat 2

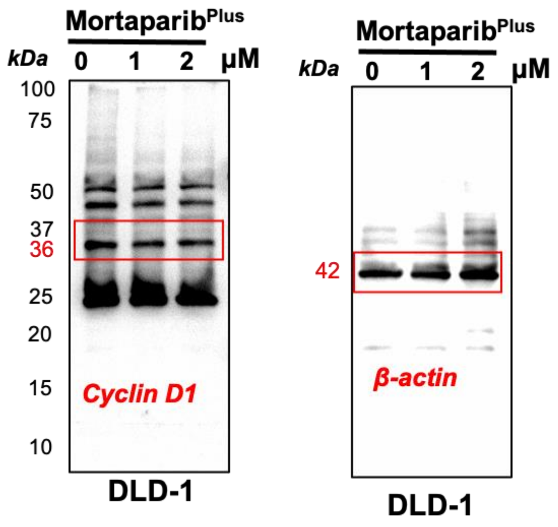

Repeat 3

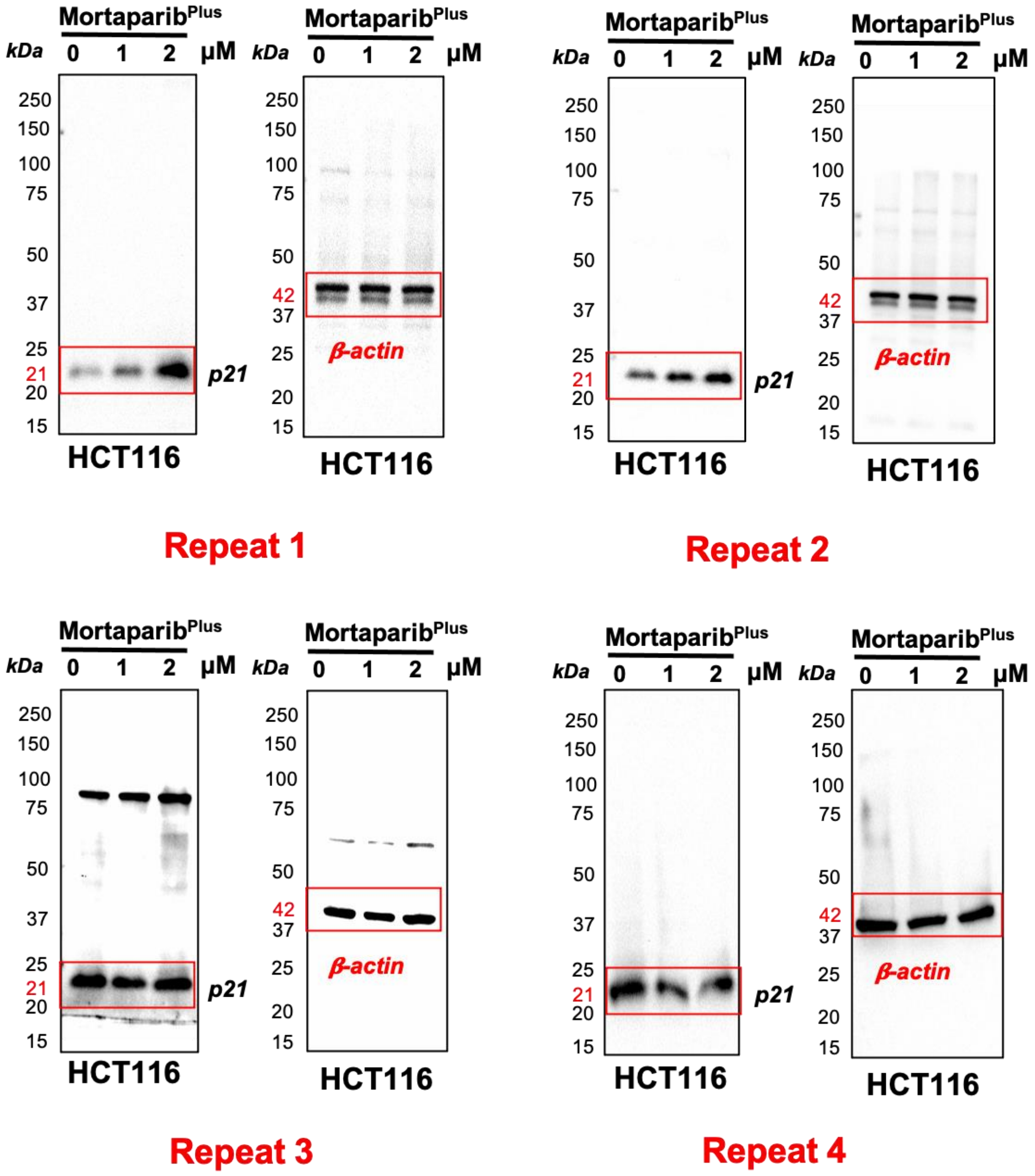
Mortaparib^Plus
kDa 0 1 2 μM
250
150
100
75
50
37
25
21
20
15
p21
HCT116
42
β-actin
Repeat 1
Repeat 2
Repeat 3
Repeat 4

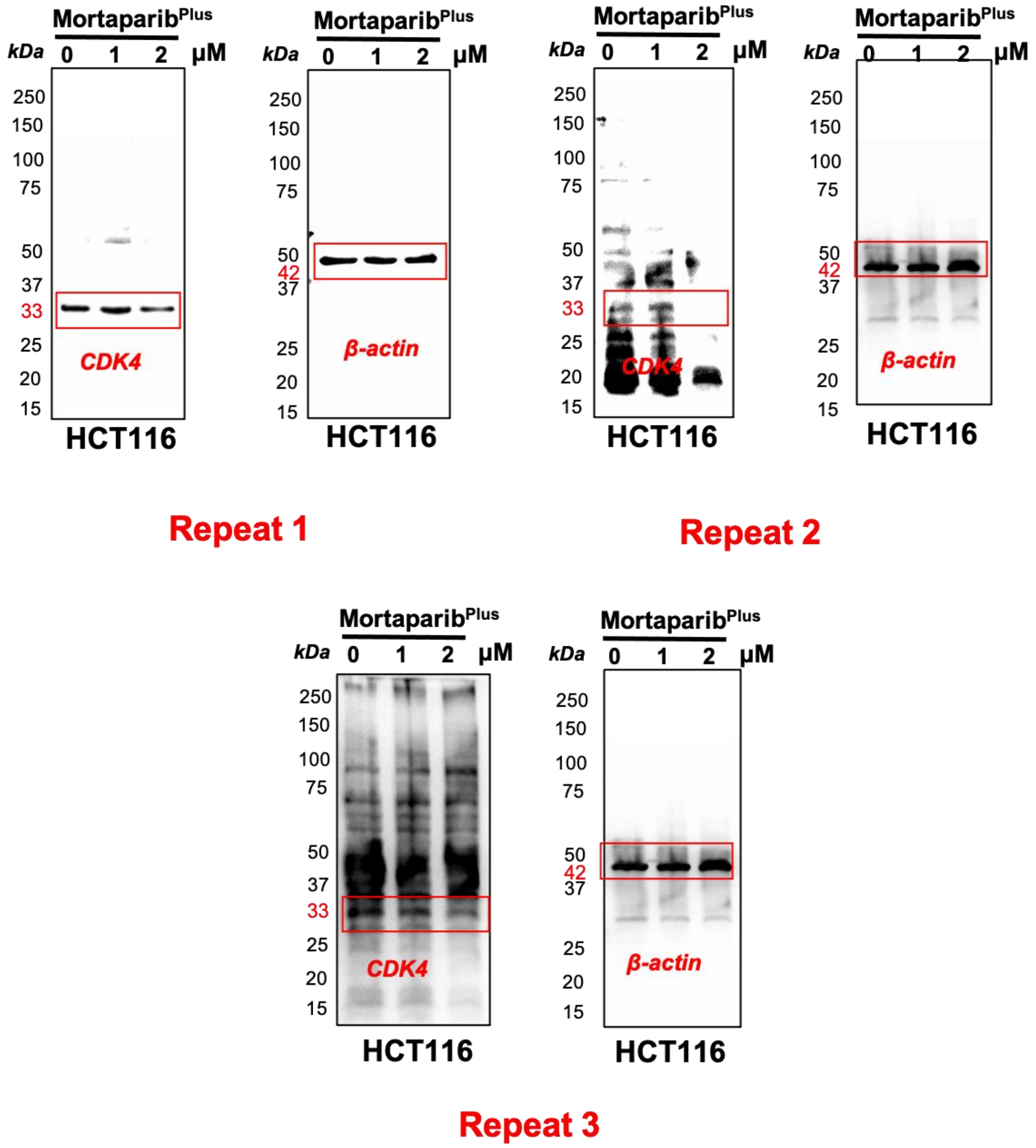

MortaparibPlus
kDa
0 1 2 μM
250
150
100
75
50
42
37
33
25
20
15
CDK4
β-actin
HCT116
Repeat 1
Repeat 2
Repeat 3

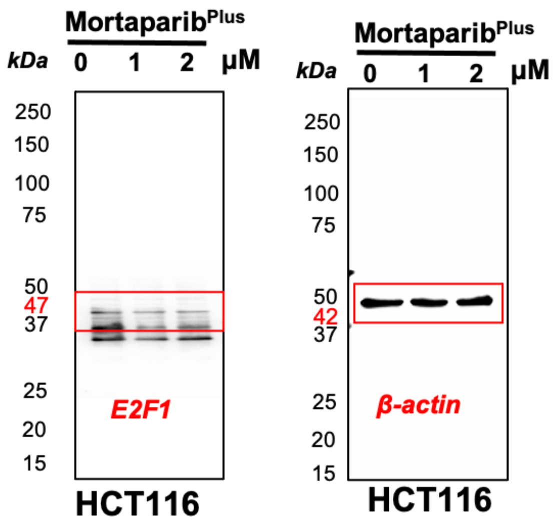

Repeat 1

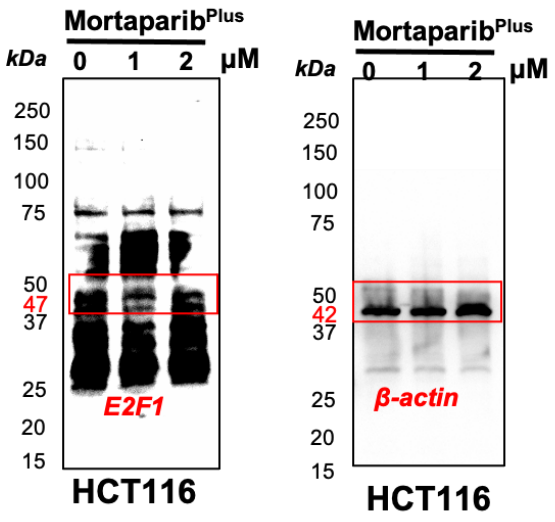

Repeat 2

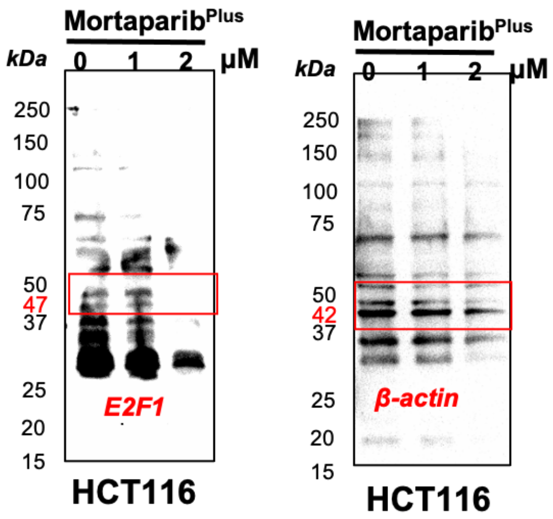

Repeat 3

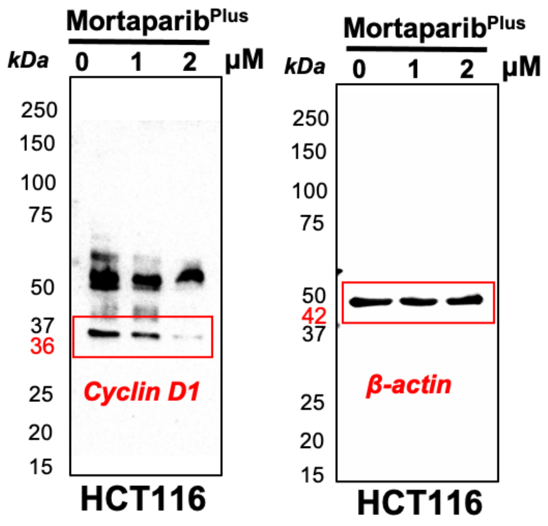

Repeate 1

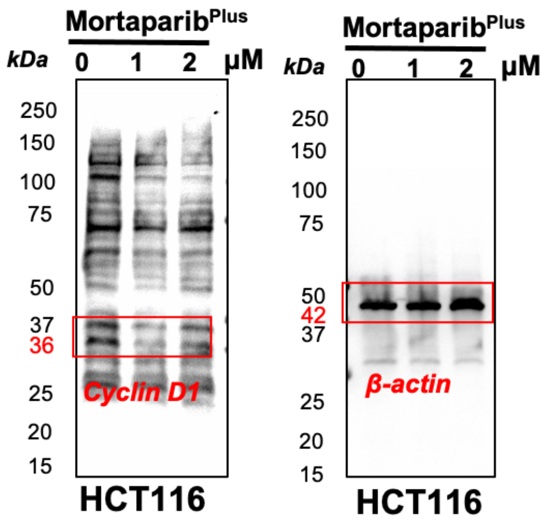

Repeate 2

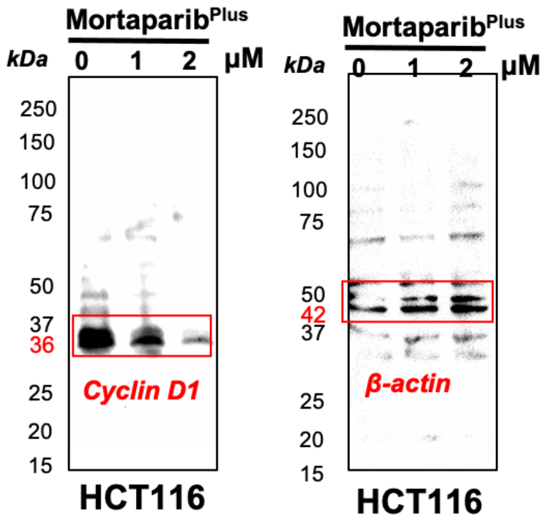

Repeate 3

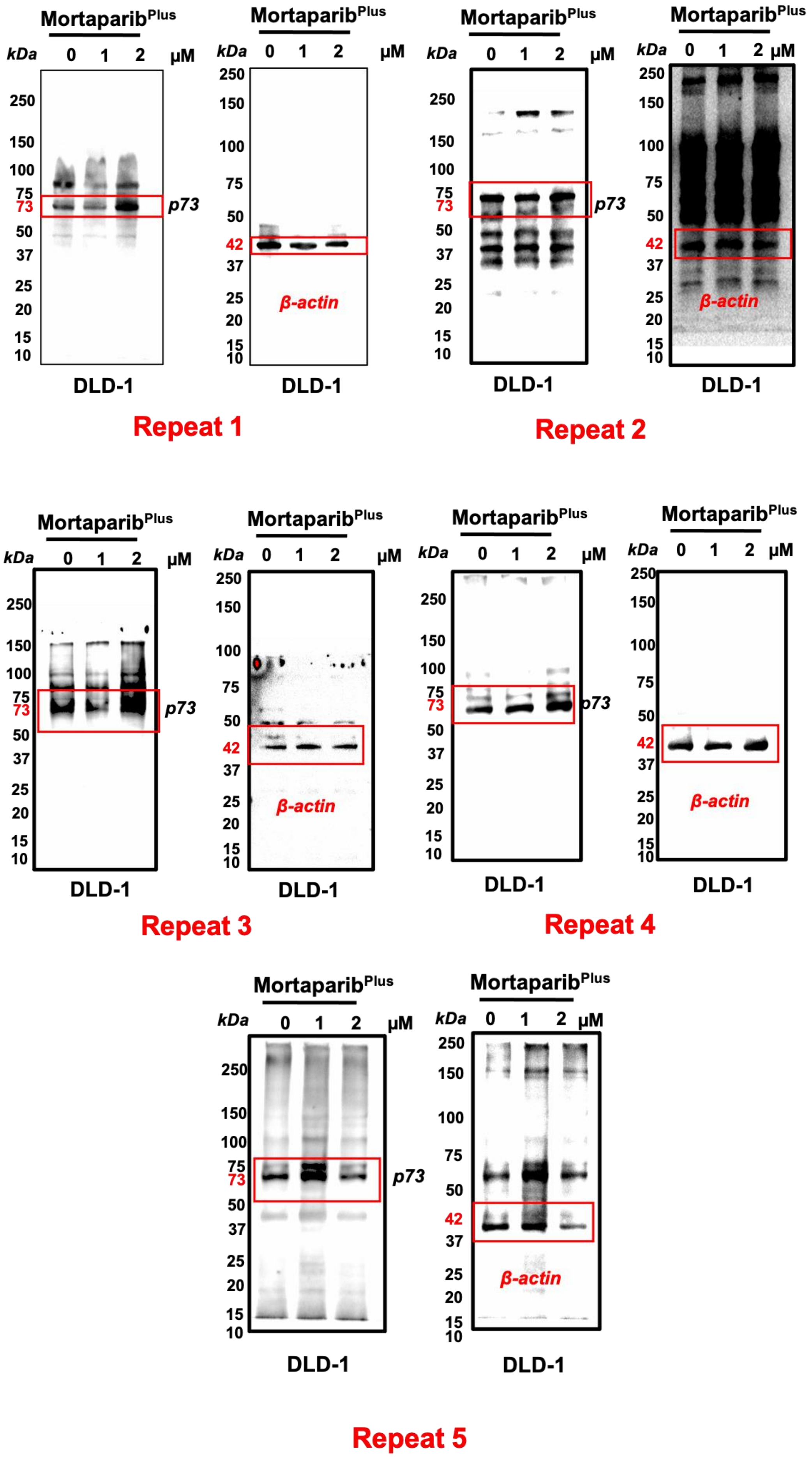
MortaparibPlus
kDa 0 1 2 µM
250 150 100 75 73 50 37 25 20 15 10
p73
β-actin
42
DLD-1
Repeat 1
Repeat 2
Repeat 3
Repeat 4
Repeat 5

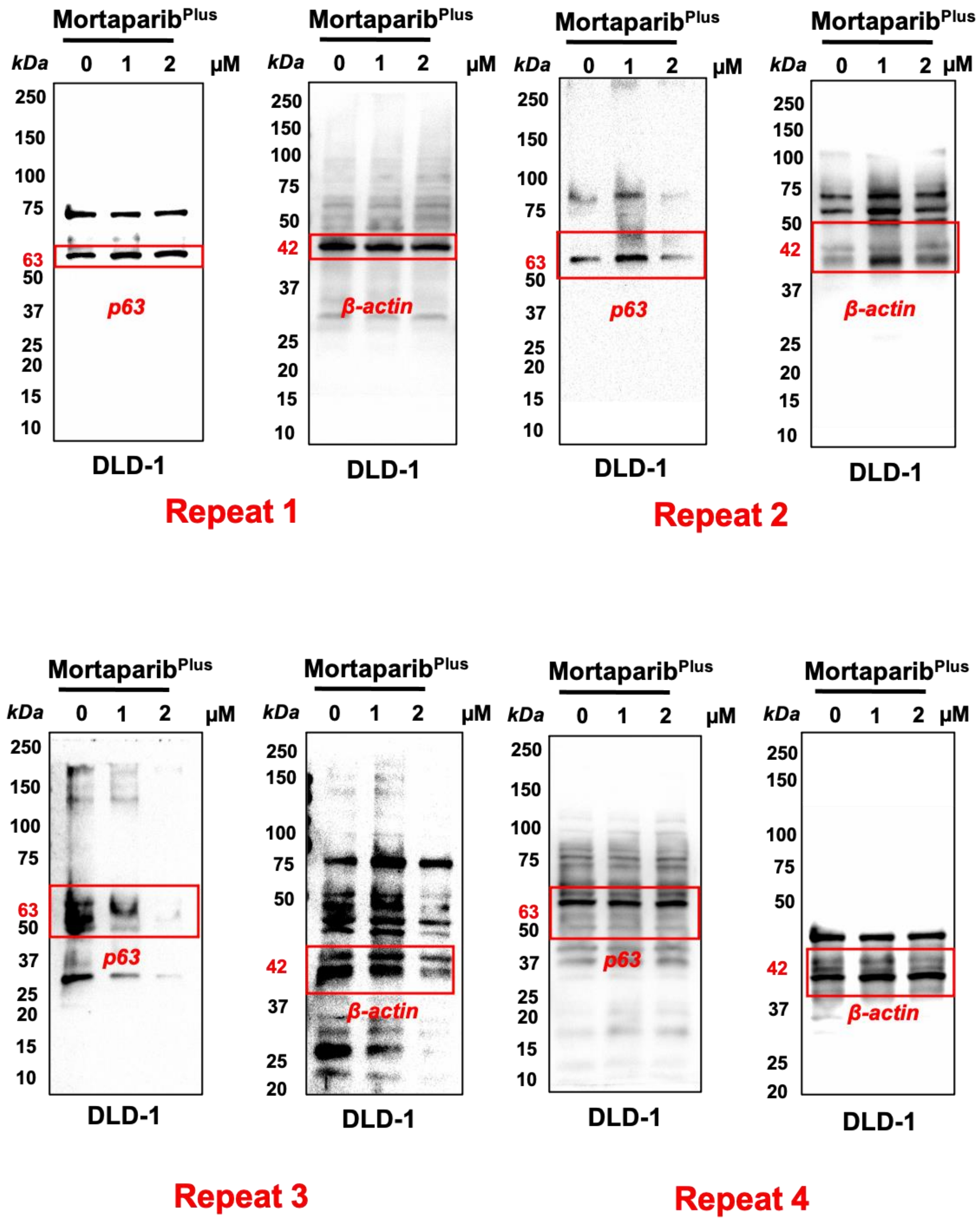

Mortaparib^Plus
kDa 0 1 2 μM
250 150 100 75 63 50 37 25 20 15 10
p63
DLD-1
Mortaparib^Plus
kDa 0 1 2 μM
250 150 100 75 50 42 37 25 20 15 10
β-actin
DLD-1
Repeat 1
Mortaparib^Plus
kDa 0 1 2 μM
250 150 100 75 63 50 37 25 20 15 10
p63
DLD-1
Mortaparib^Plus
kDa 0 1 2 μM
250 150 100 75 50 42 37 25 20 15 10
β-actin
DLD-1
Repeat 2
Mortaparib^Plus
kDa 0 1 2 μM
250 150 100 75 63 50 37 25 20 15 10
p63
DLD-1
Mortaparib^Plus
kDa 0 1 2 μM
250 150 100 75 50 42 37 25 20
β-actin
DLD-1
Repeat 3
Mortaparib^Plus
kDa 0 1 2 μM
250 150 100 75 63 50 37 25 20 15 10
p63
DLD-1
Mortaparib^Plus
kDa 0 1 2 μM
250 150 100 75 50 42 37 25 20
β-actin
DLD-1
Repeat 4

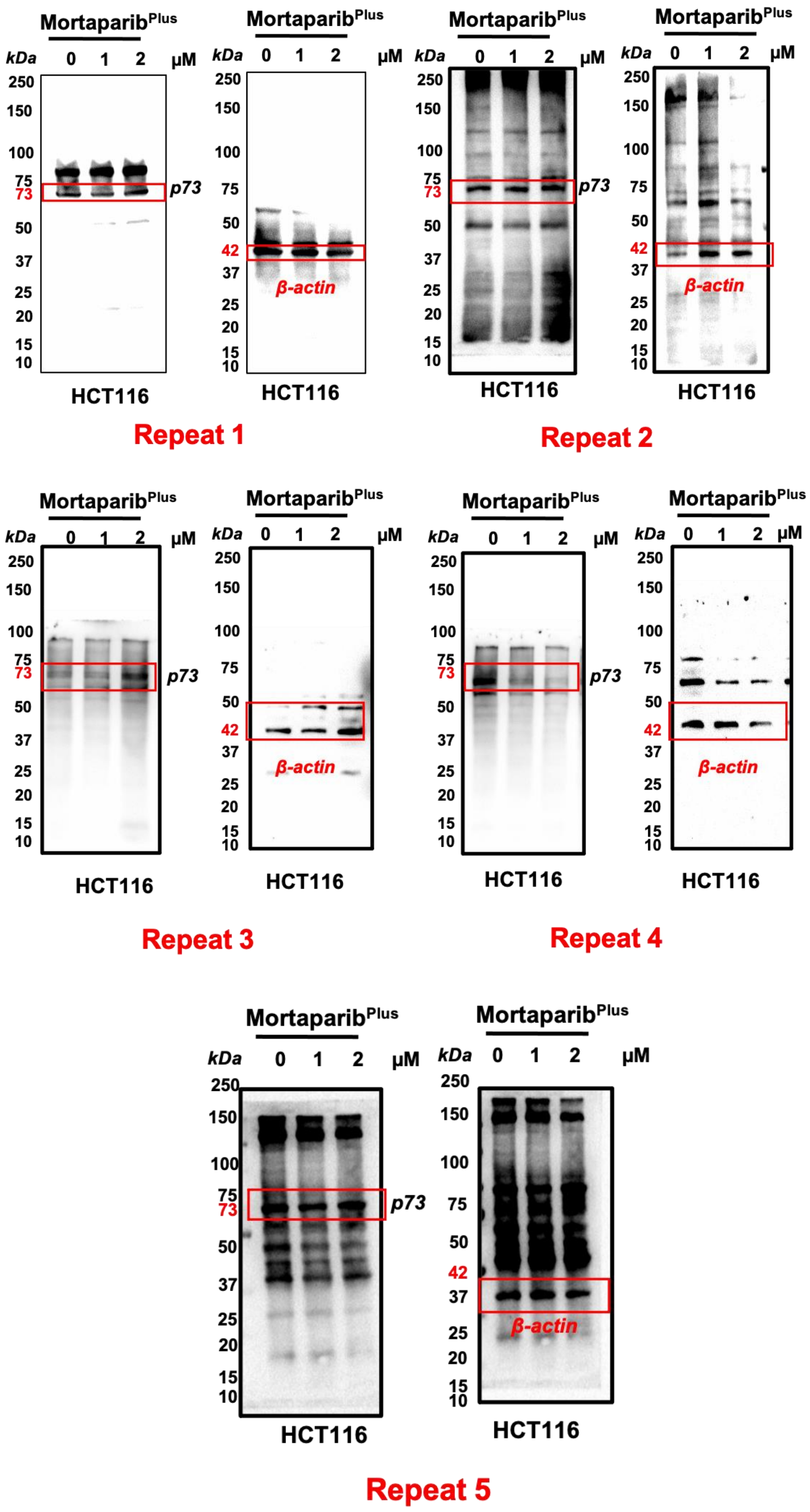
MortaparibPlus
kDa
0 1 2 µM
250
150
100
75
73
50
42
37
25
20
15
10
p73
β-actin
HCT116
Repeat 1
Repeat 2
Repeat 3
Repeat 4
Repeat 5

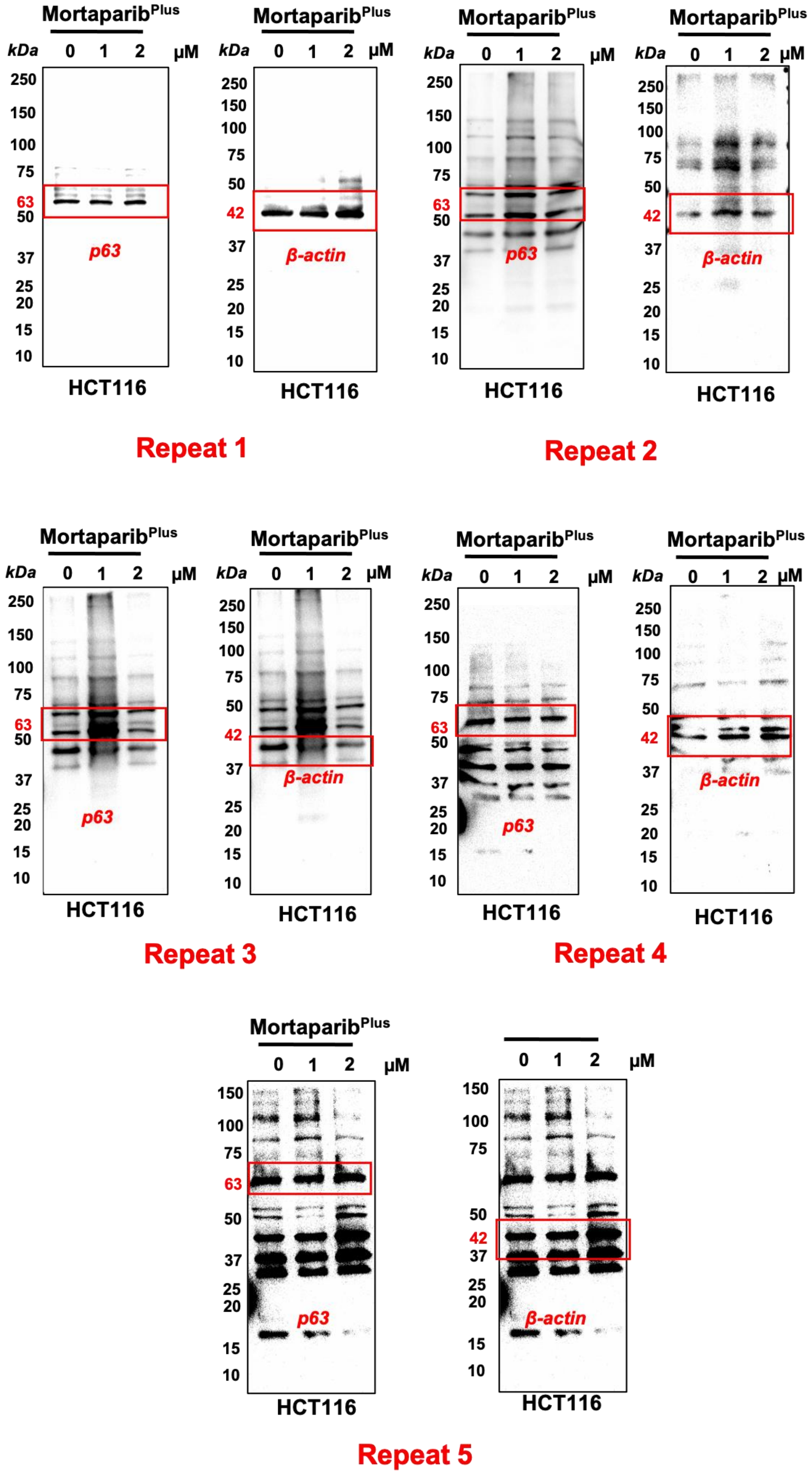

MortaparibPlus
kDa
0 1 2 µM
250
150
100
75
63
50
37
25
20
15
10
p63
42
β-actin
HCT116
Repeat 1
Repeat 2
Repeat 3
Repeat 4
Repeat 5

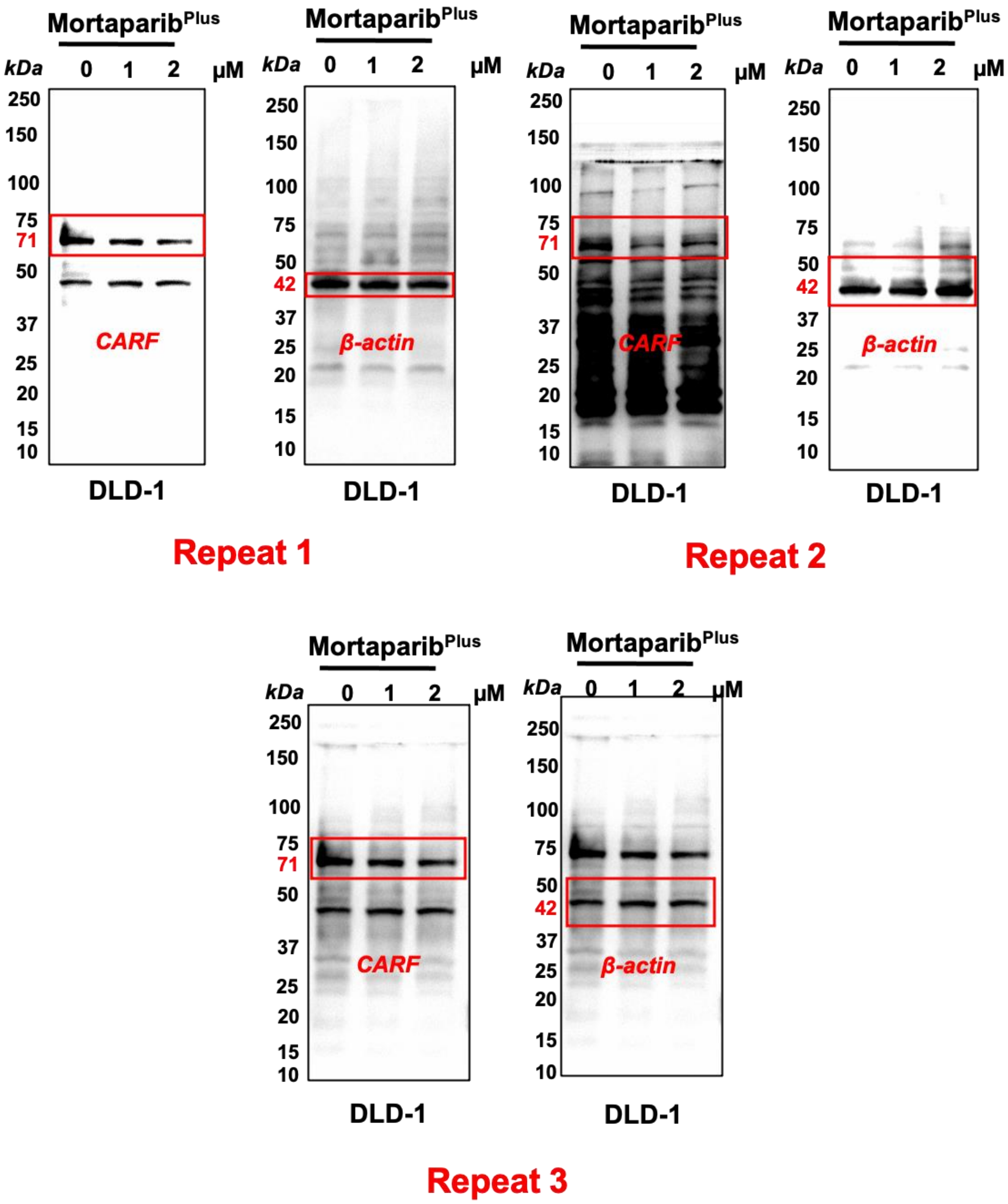

MortaparibPlus
kDa 0 1 2 μM
250 150 100 75 71 50 37 25 20 15 10
CARF
DLD-1
MortaparibPlus
kDa 0 1 2 μM
250 150 100 75 50 42 37 25 20 15 10
β-actin
DLD-1
Repeat 1
MortaparibPlus
kDa 0 1 2 μM
250 150 100 75 71 50 37 25 20 15 10
CARF
DLD-1
MortaparibPlus
kDa 0 1 2 μM
250 150 100 75 50 42 37 25 20 15 10
β-actin
DLD-1
Repeat 2
MortaparibPlus
kDa 0 1 2 μM
250 150 100 75 71 50 37 25 20 15 10
CARF
DLD-1
MortaparibPlus
kDa 0 1 2 μM
250 150 100 75 50 42 37 25 20 15 10
β-actin
DLD-1
Repeat 3

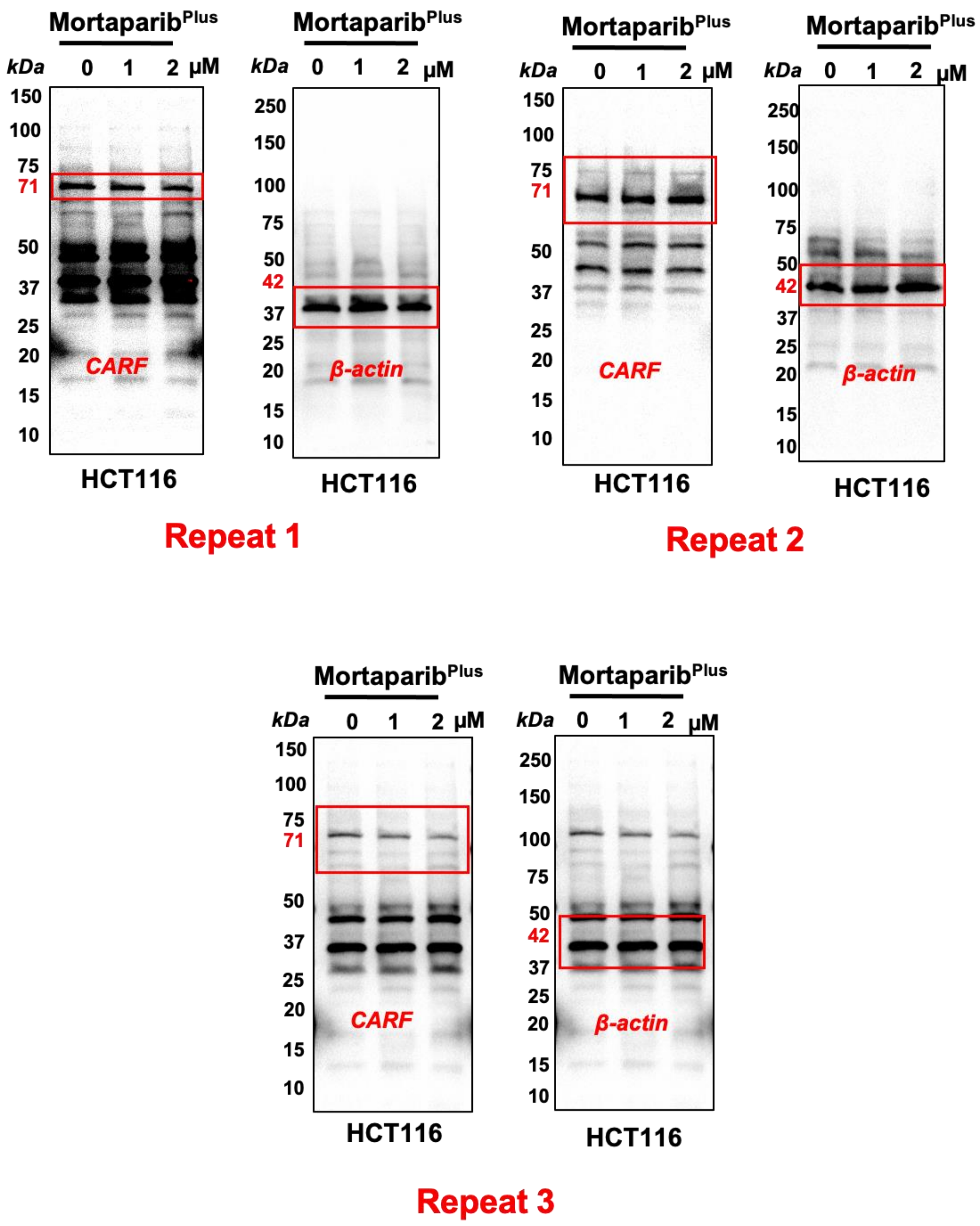

MortaparibPlus
kDa 0 1 2 μM
150 100 75 71 50 37 25 20 15 10
CARF
HCT116
MortaparibPlus
kDa 0 1 2 μM
250 150 100 75 50 42 37 25 20 15 10
β-actin
HCT116
Repeat 1
MortaparibPlus
kDa 0 1 2 μM
150 100 75 71 50 37 25 20 15 10
CARF
HCT116
MortaparibPlus
kDa 0 1 2 μM
250 150 100 75 50 42 37 25 20 15 10
β-actin
HCT116
Repeat 2
MortaparibPlus
kDa 0 1 2 μM
150 100 75 71 50 37 25 20 15 10
CARF
HCT116
MortaparibPlus
kDa 0 1 2 μM
250 150 100 75 50 42 37 25 20 15 10
β-actin
HCT116
Repeat 3

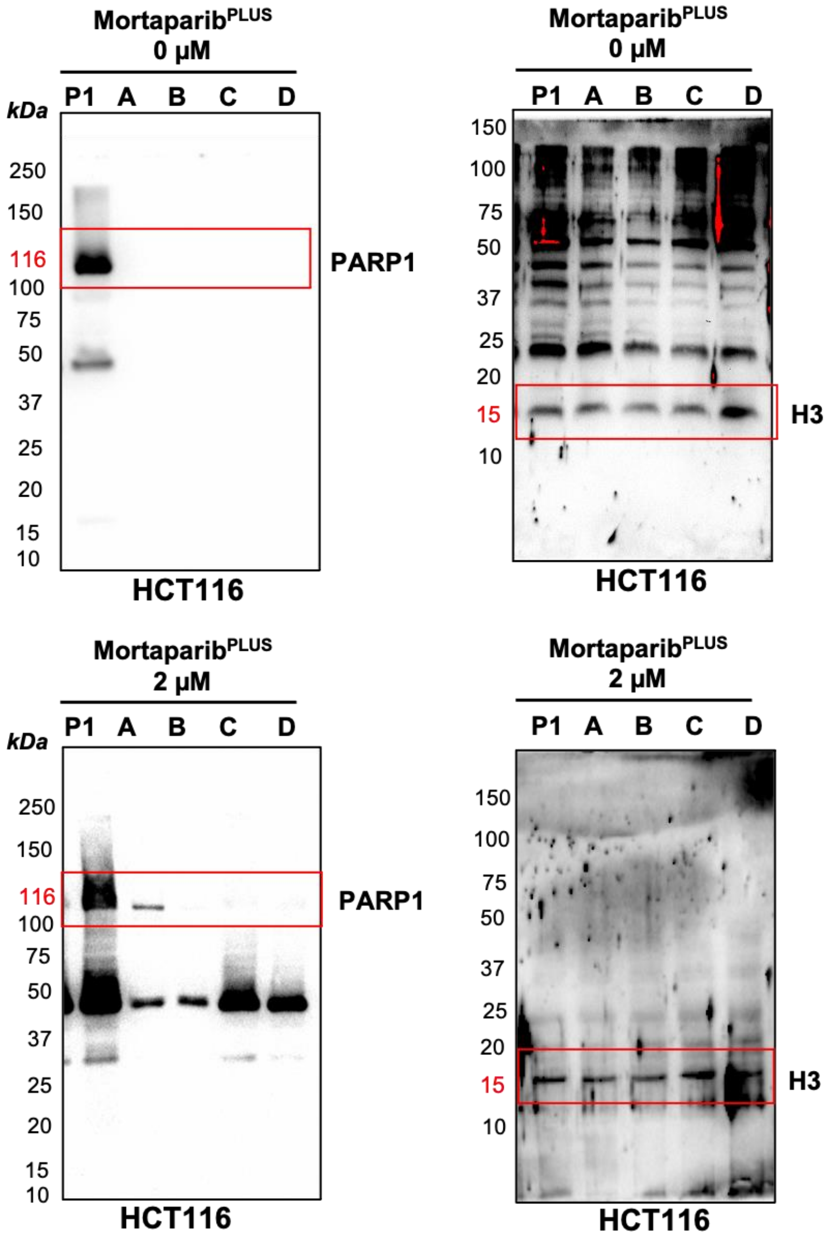

P1= Whole cell lysate in Hypotonic buffer

A= Precipitant A in buffer **A**
B= Precipitant B in buffer **B**

C= Precipitant C in buffer **C**
D= Precipitant D in buffer **D**

Repeat 1

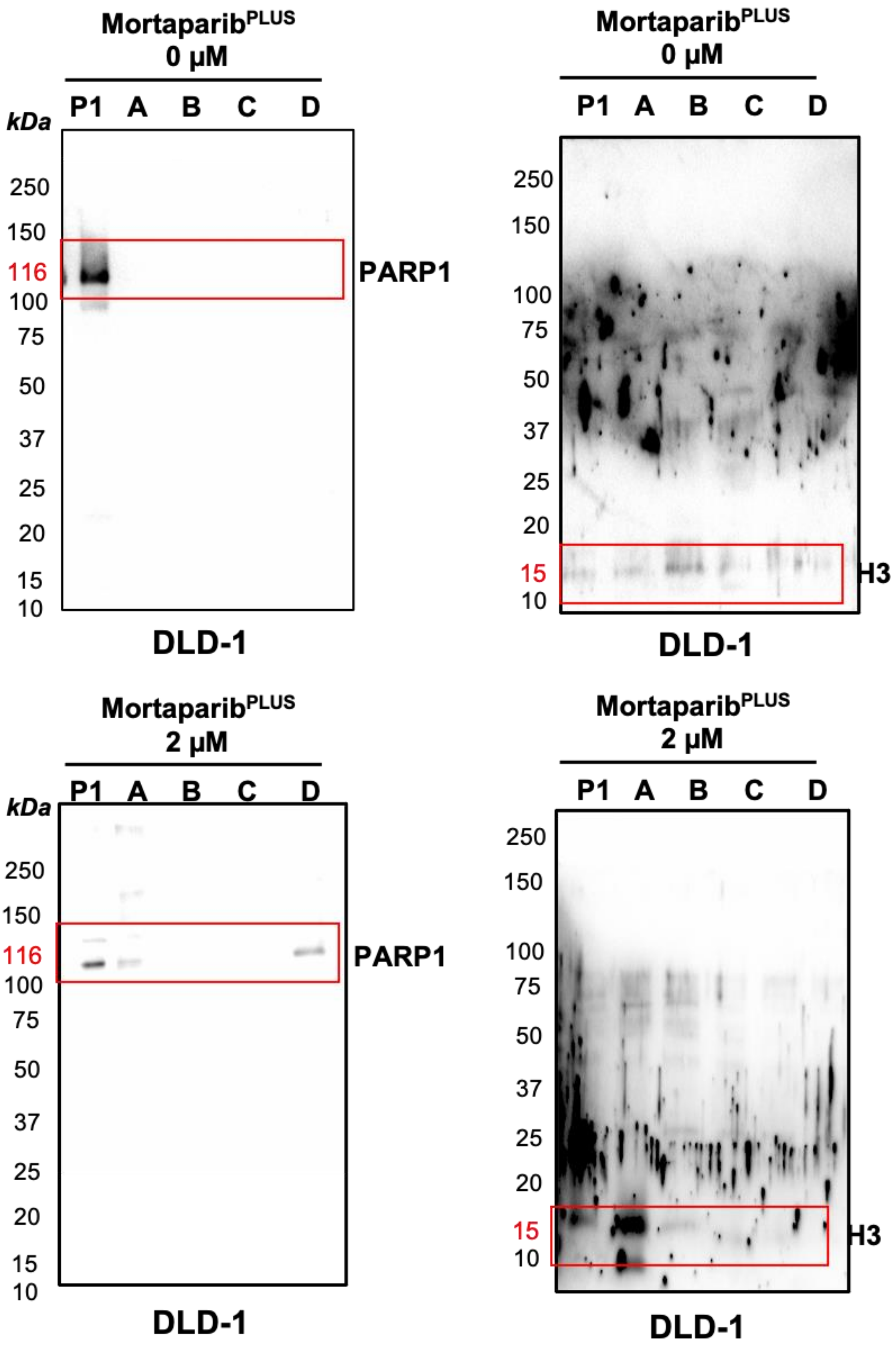

P1= Whole cell lysate in Hypotonic buffer

A= Precipitant A in buffer **A**
B= Precipitant B in buffer **B**

Repeat 2

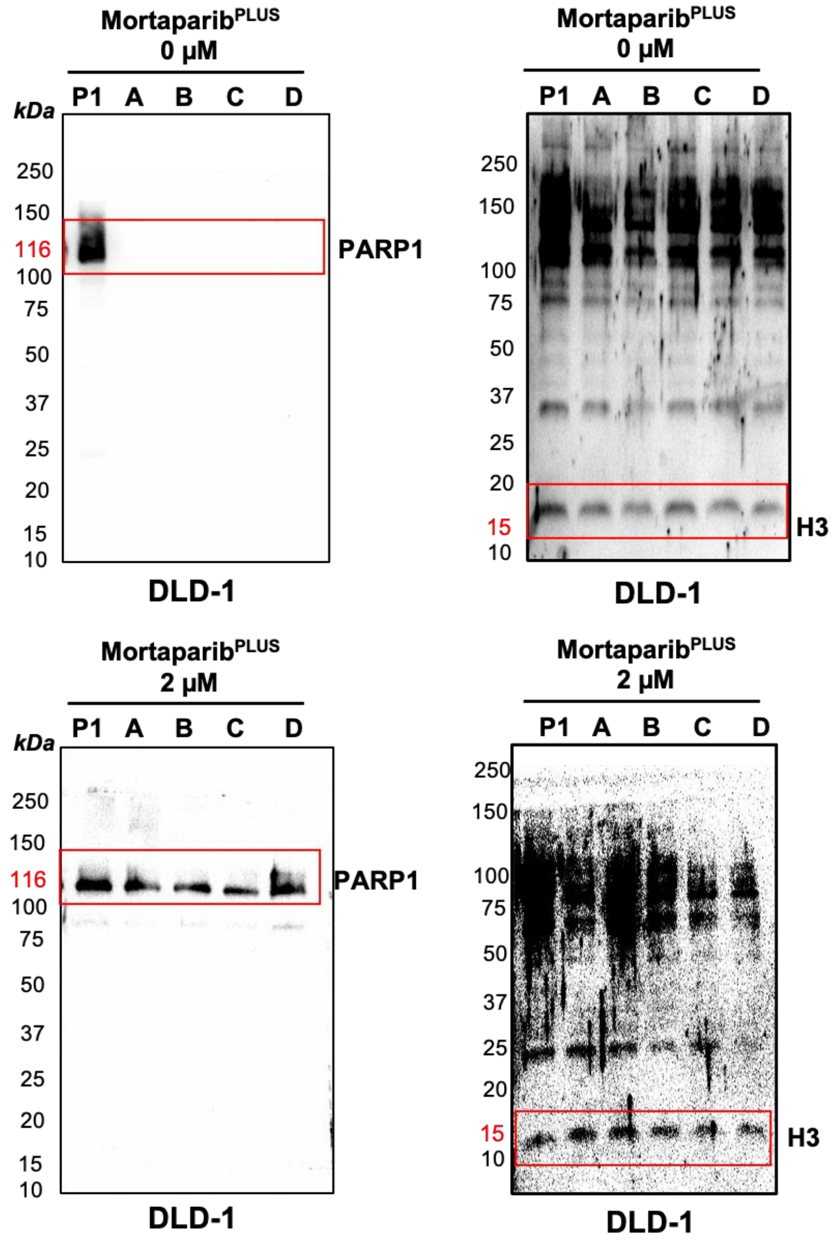

P1= Whole cell lysate in Hypotonic buffer
A= Precipitant A in buffer **A**
B= Precipitant B in buffer **B**
C= Precipitant C in buffer **C**
D= Precipitant D in buffer **D**

Repeat 3

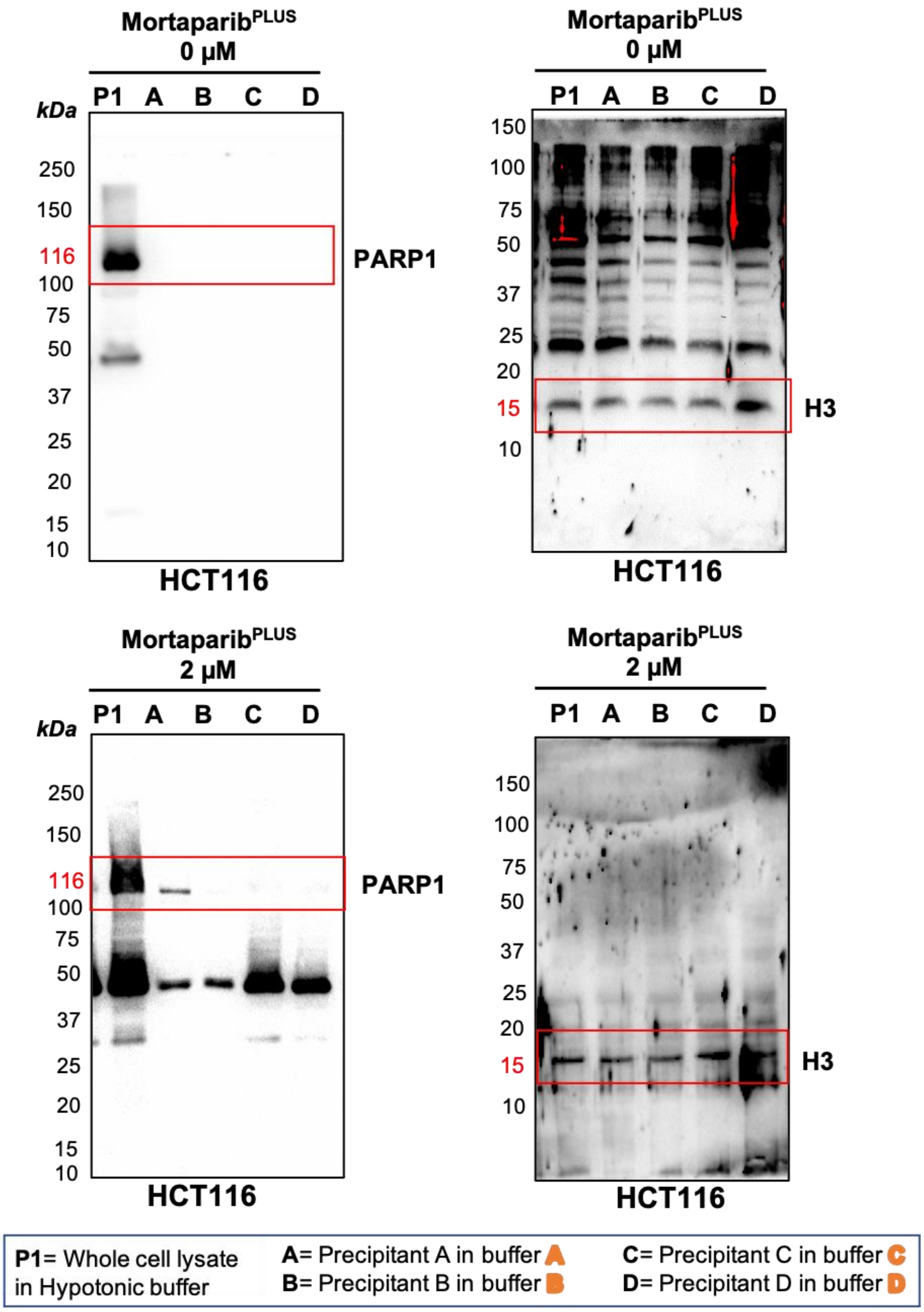

MortaparibPLUS 0 µM
P1 A B C D
kDa
250
150
116
100
75
50
37
25
20
15
10
PARP1
HCT116
MortaparibPLUS 0 µM
P1 A B C D
150
100
75
50
37
25
20
15
10
H3
HCT116
MortaparibPLUS 2 µM
P1 A B C D
kDa
250
150
116
100
75
50
37
25
20
15
10
PARP1
HCT116
MortaparibPLUS 2 µM
P1 A B C D
150
100
75
50
37
25
20
15
10
H3
HCT116
P1= Whole cell lysate in Hypotonic buffer
A= Precipitant A in buffer A
B= Precipitant B in buffer B
C= Precipitant C in buffer C
D= Precipitant D in buffer D

Repeat 1

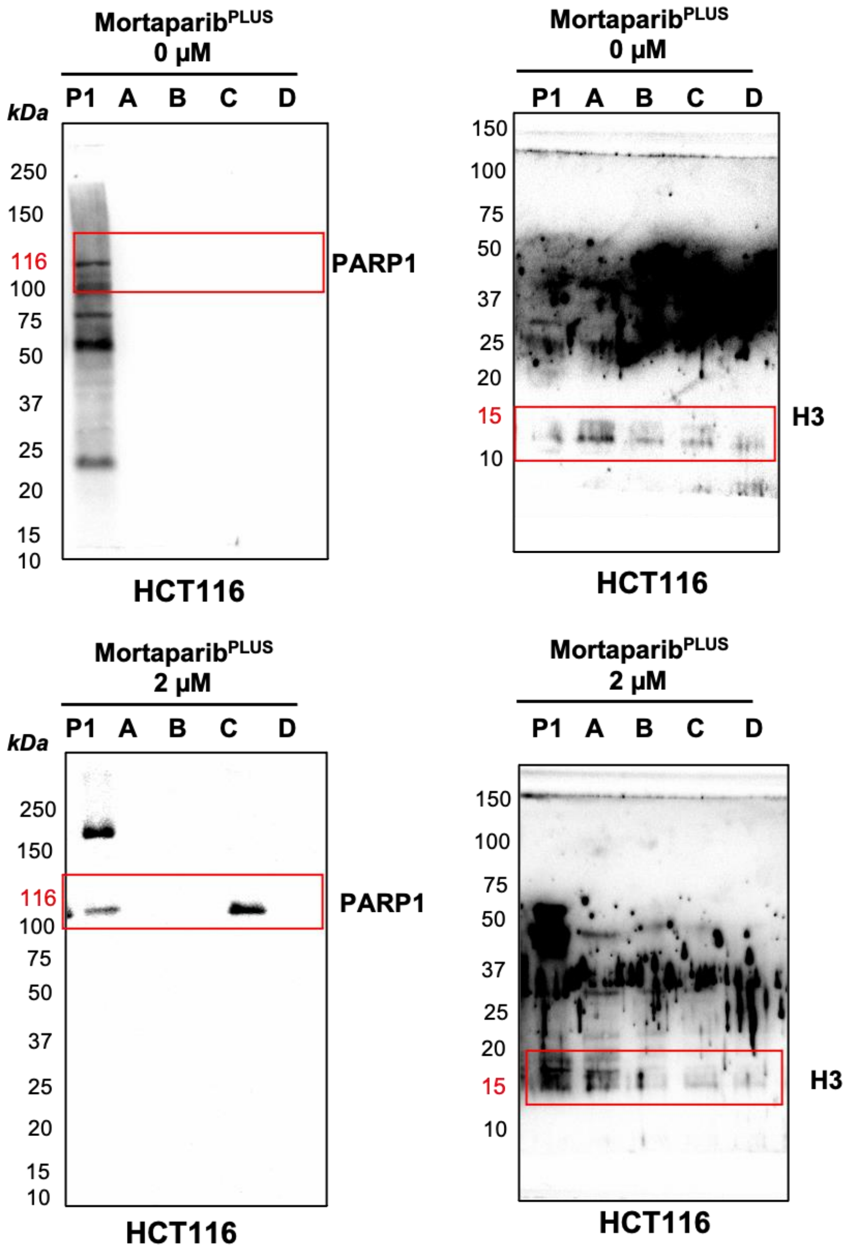

P1= Whole cell lysate in Hypotonic buffer
A= Precipitant A in buffer **A**
B= Precipitant B in buffer **B**
C= Precipitant C in buffer **C**
D= Precipitant D in buffer **D**

Repeat 2

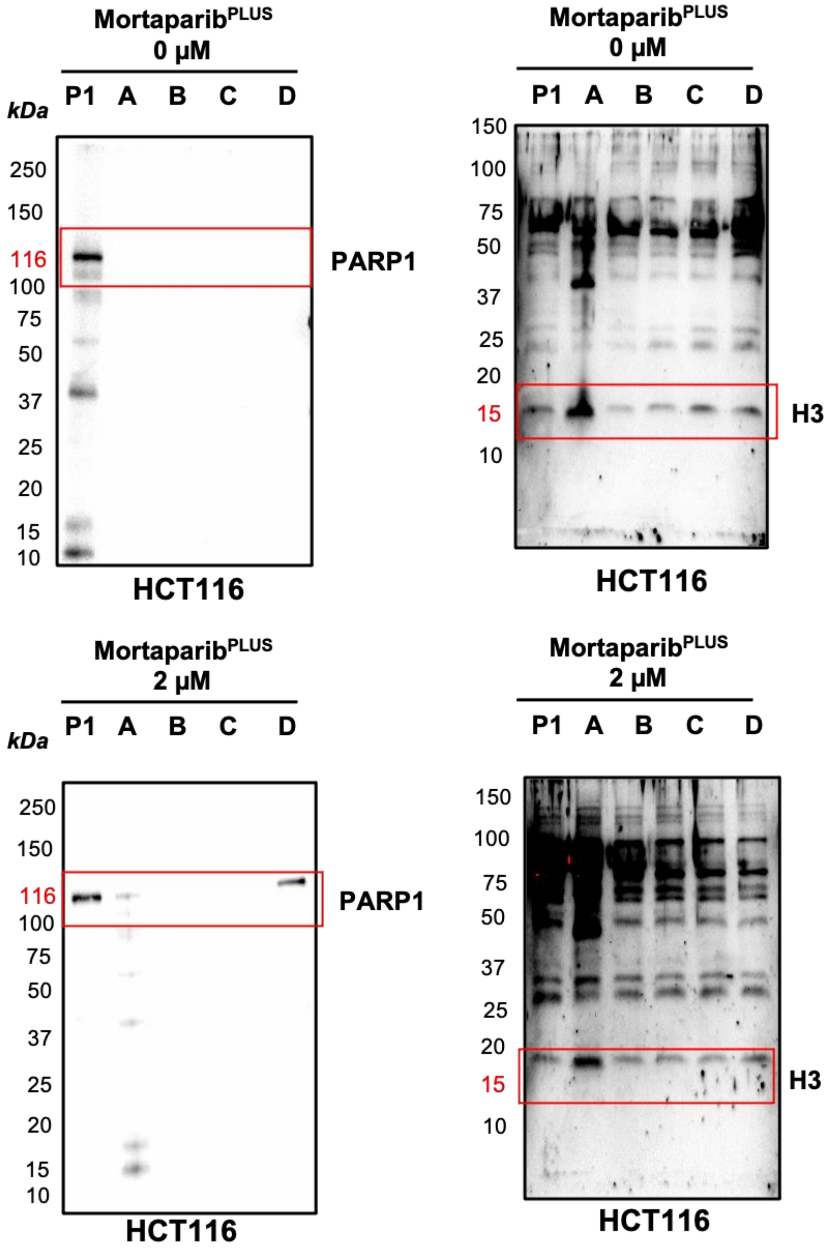

P1= Whole cell lysate in Hypotonic buffer
A= Precipitant A in buffer **A**
B= Precipitant B in buffer **B**
C= Precipitant C in buffer **C**
D= Precipitant D in buffer **D**

Repeat 3

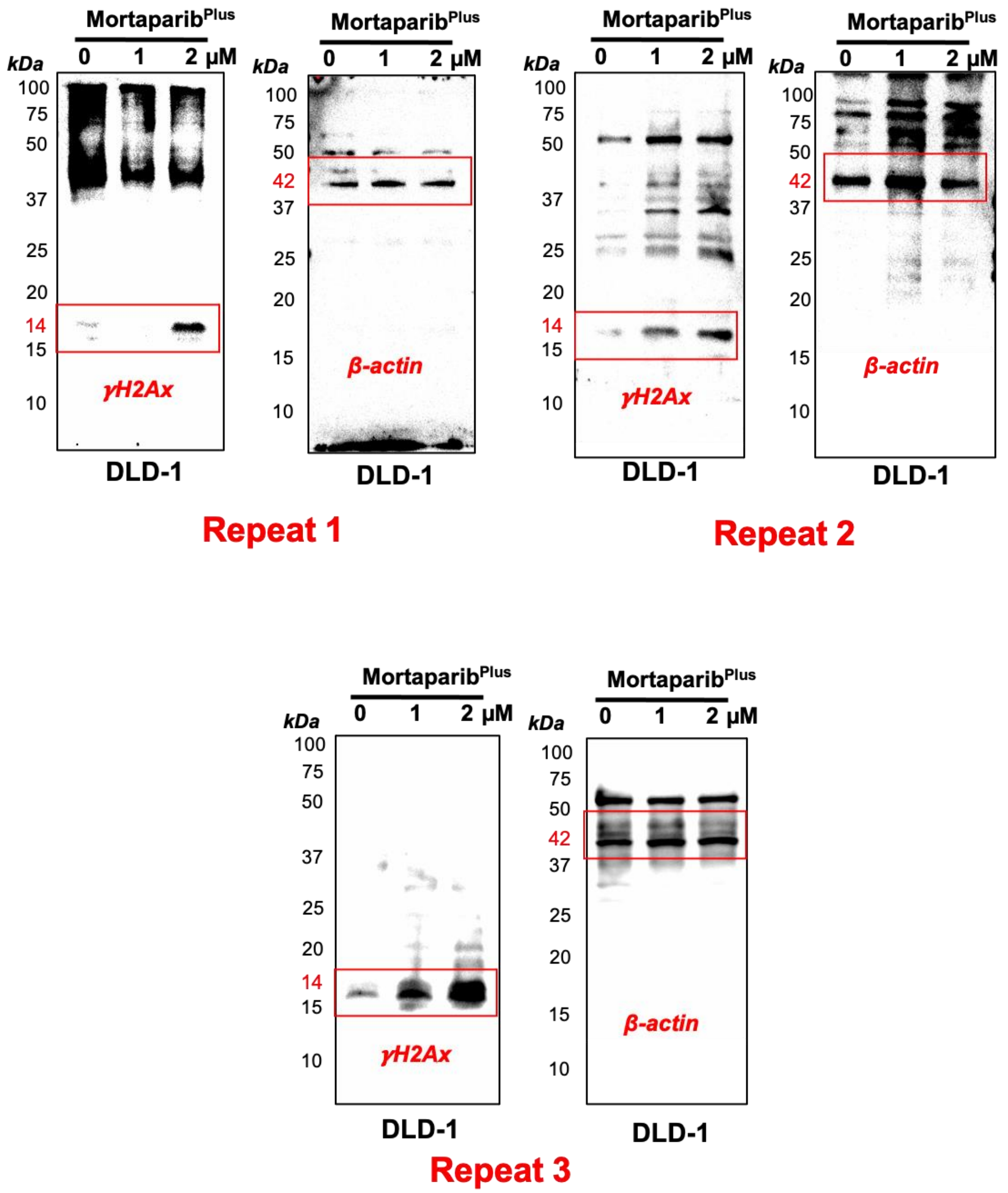
MortaparibPlus
kDa
0 1 2 μM
100
75
50
37
25
20
14
15
10
γH2Ax
β-actin
42
DLD-1
Repeat 1
Repeat 2
Repeat 3

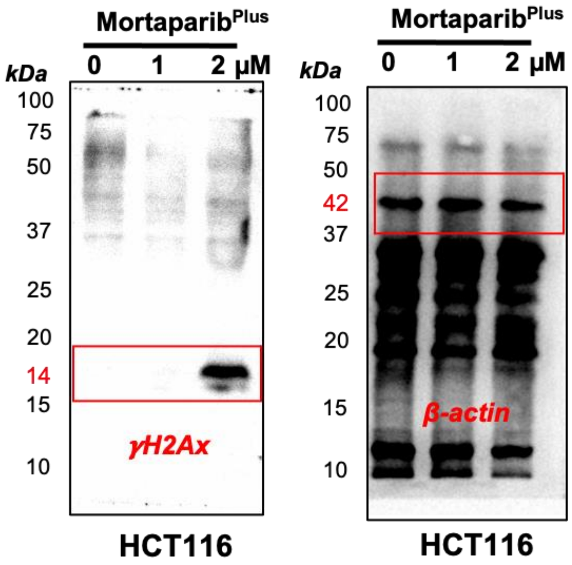

Repeat 1

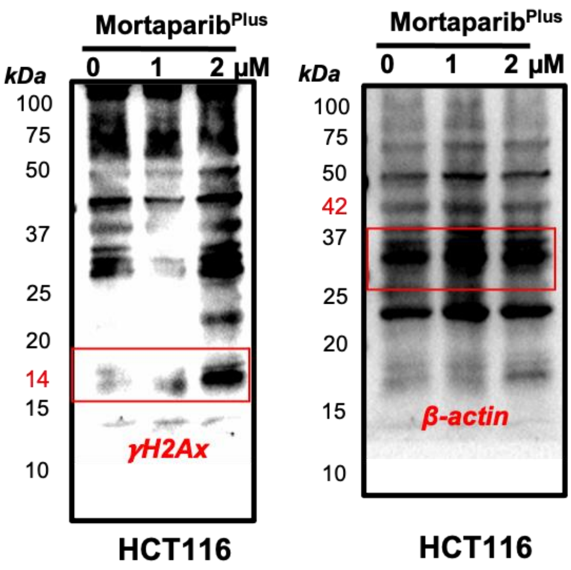

Repeat 2

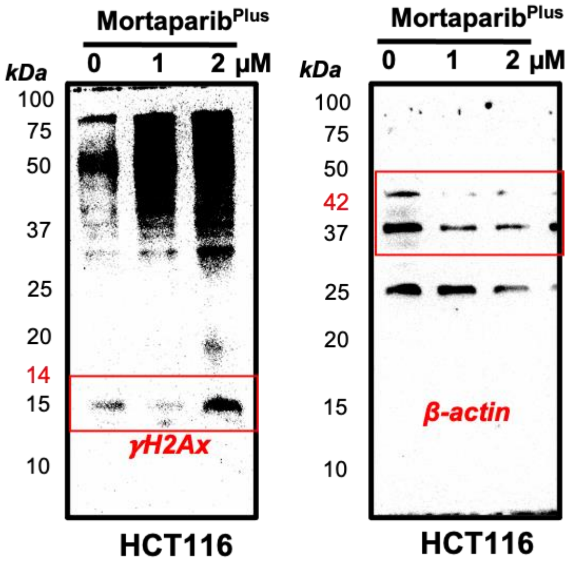

Repeat 3

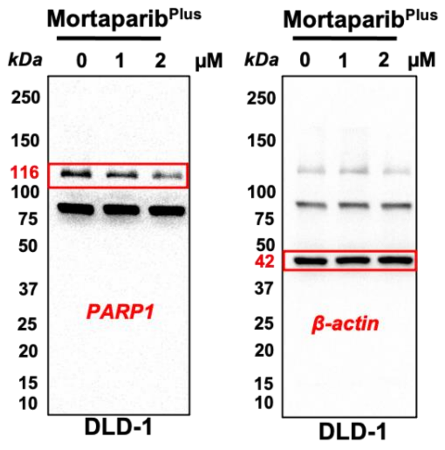

Repeat 1

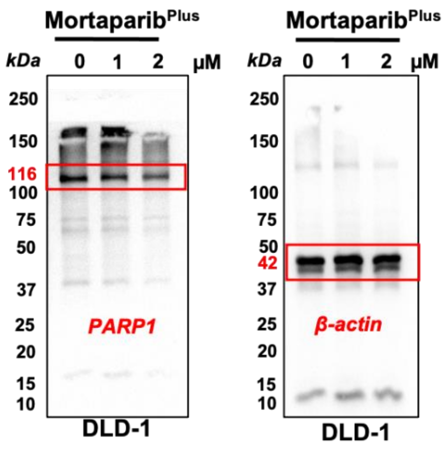

Repeat 2

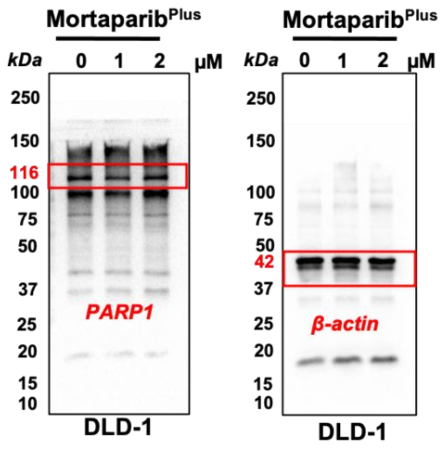

Repeat 3

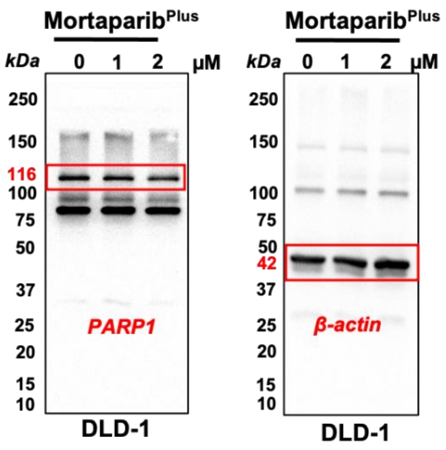

Repeat 4

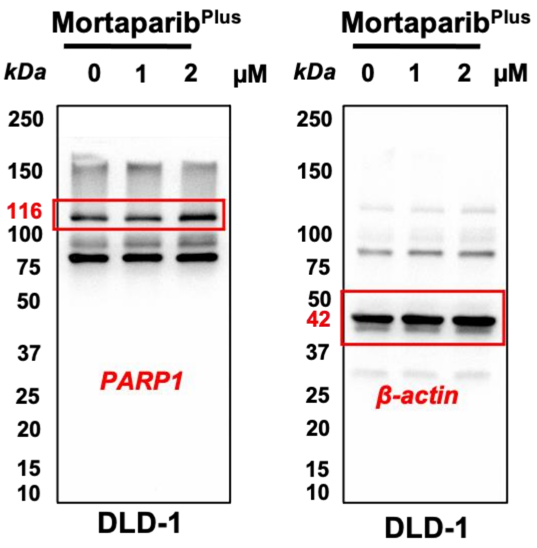

Repeat 5

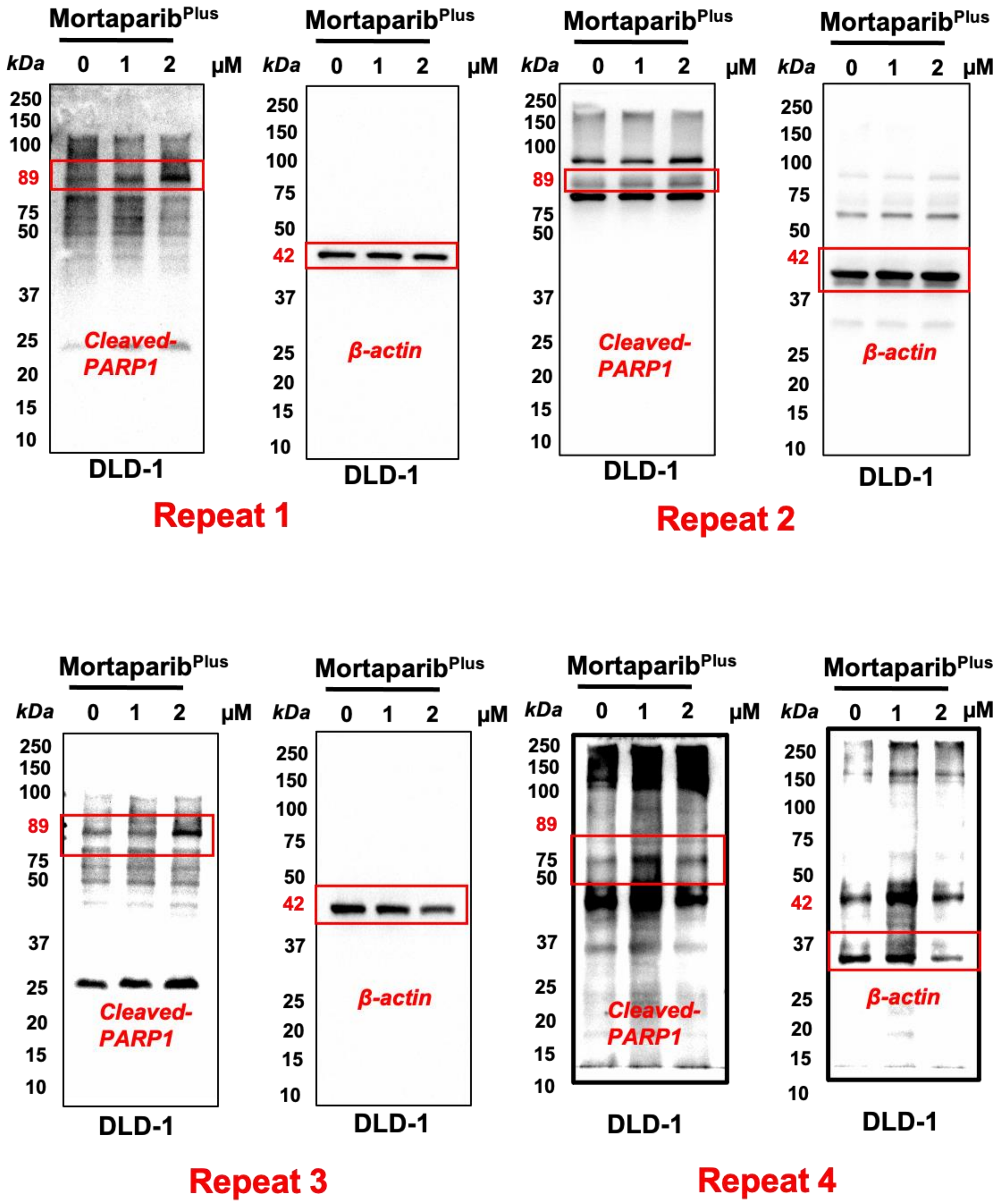
MortaparibPlus
kDa 0 1 2 μM
250
150
100
89
75
50
42
37
25
20
15
10
Cleaved-PARP1
β-actin
DLD-1
Repeat 1
Repeat 2
Repeat 3
Repeat 4

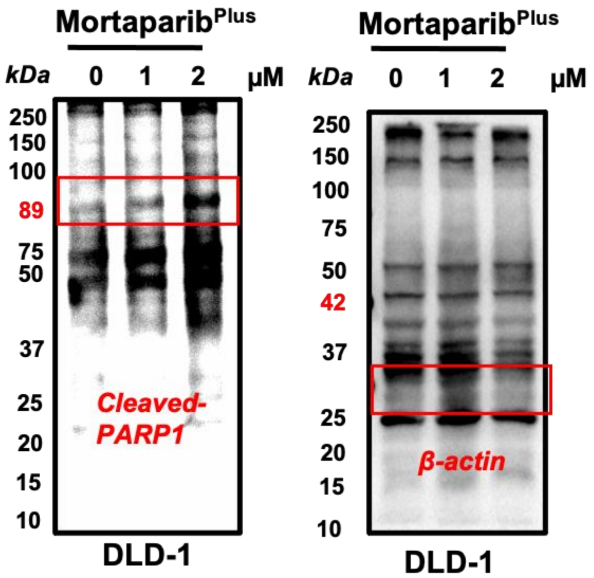

Repeat 5

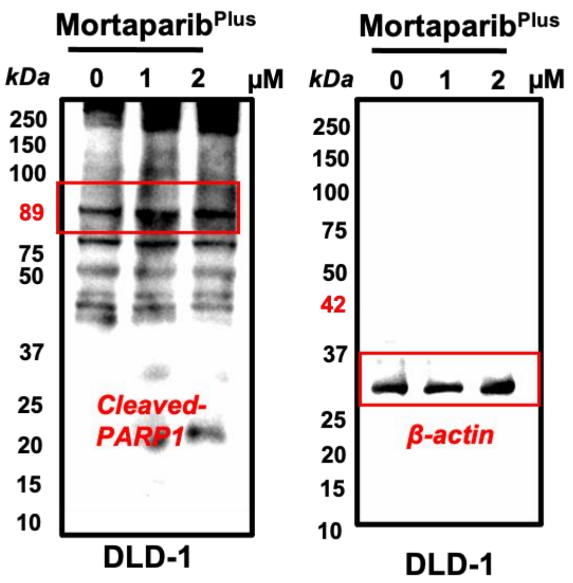

Repeat 6

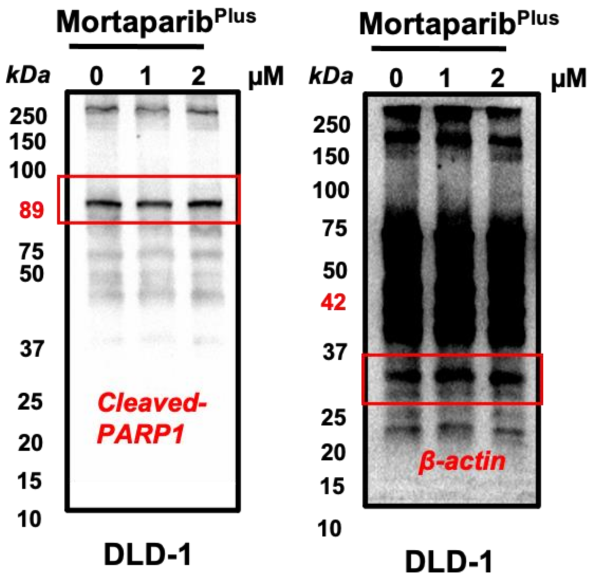

Repeat 7

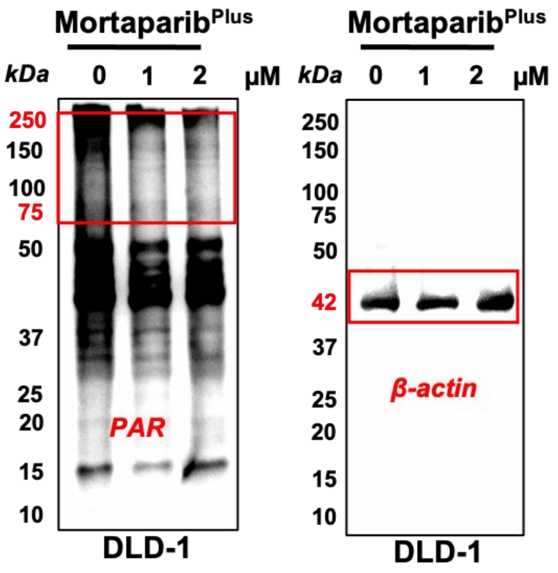

Repeat 1

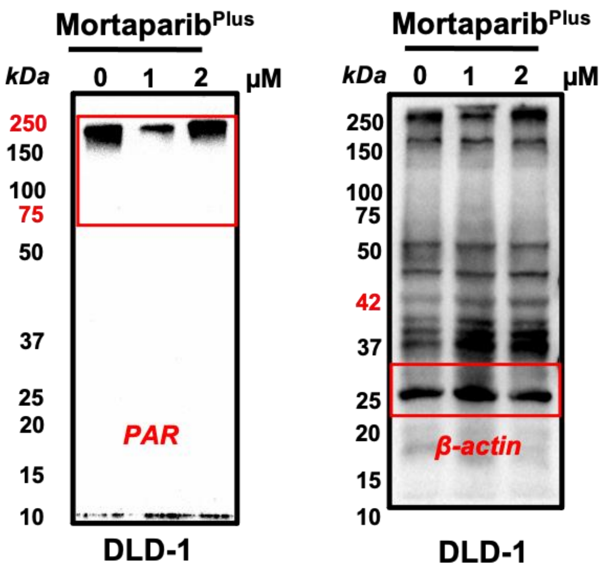

Repeat 2

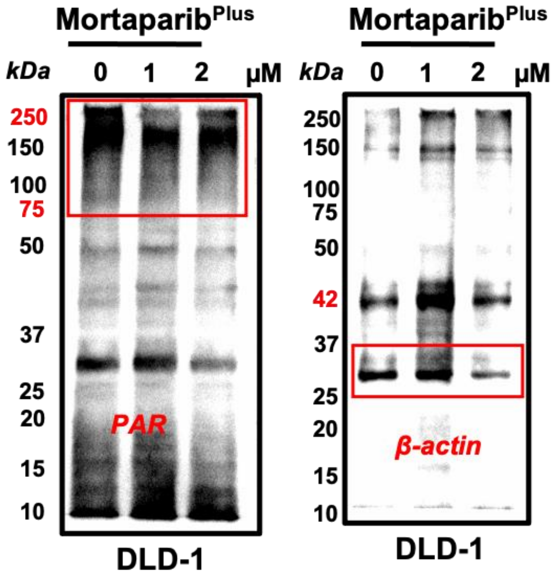

Repeat 3

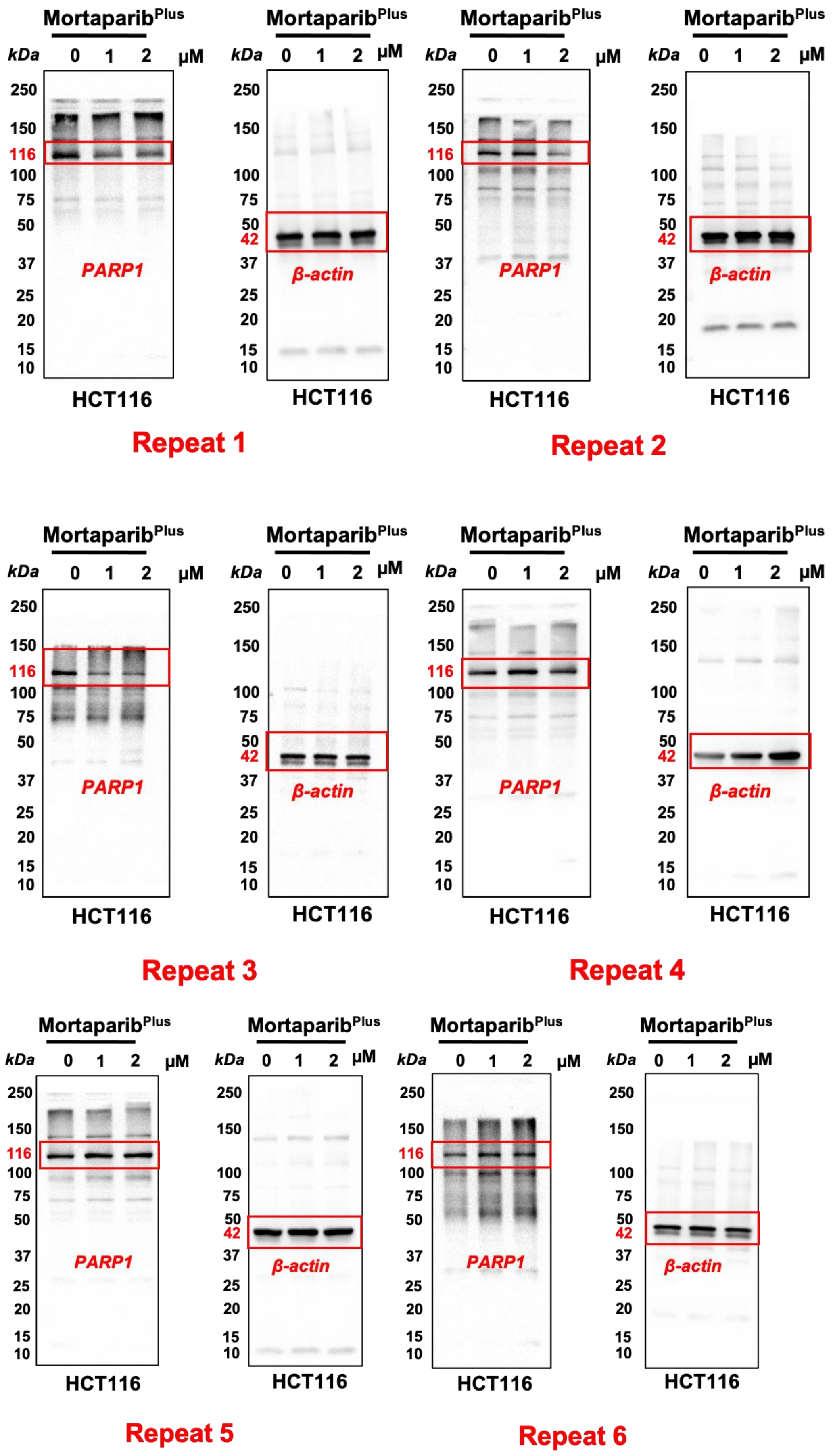
MortaparibPlus
kDa
0 1 2 μM
250
150
116
100
75
50
42
37
25
20
15
10
PARP1
β-actin
HCT116
Repeat 1
Repeat 2
Repeat 3
Repeat 4
Repeat 5
Repeat 6

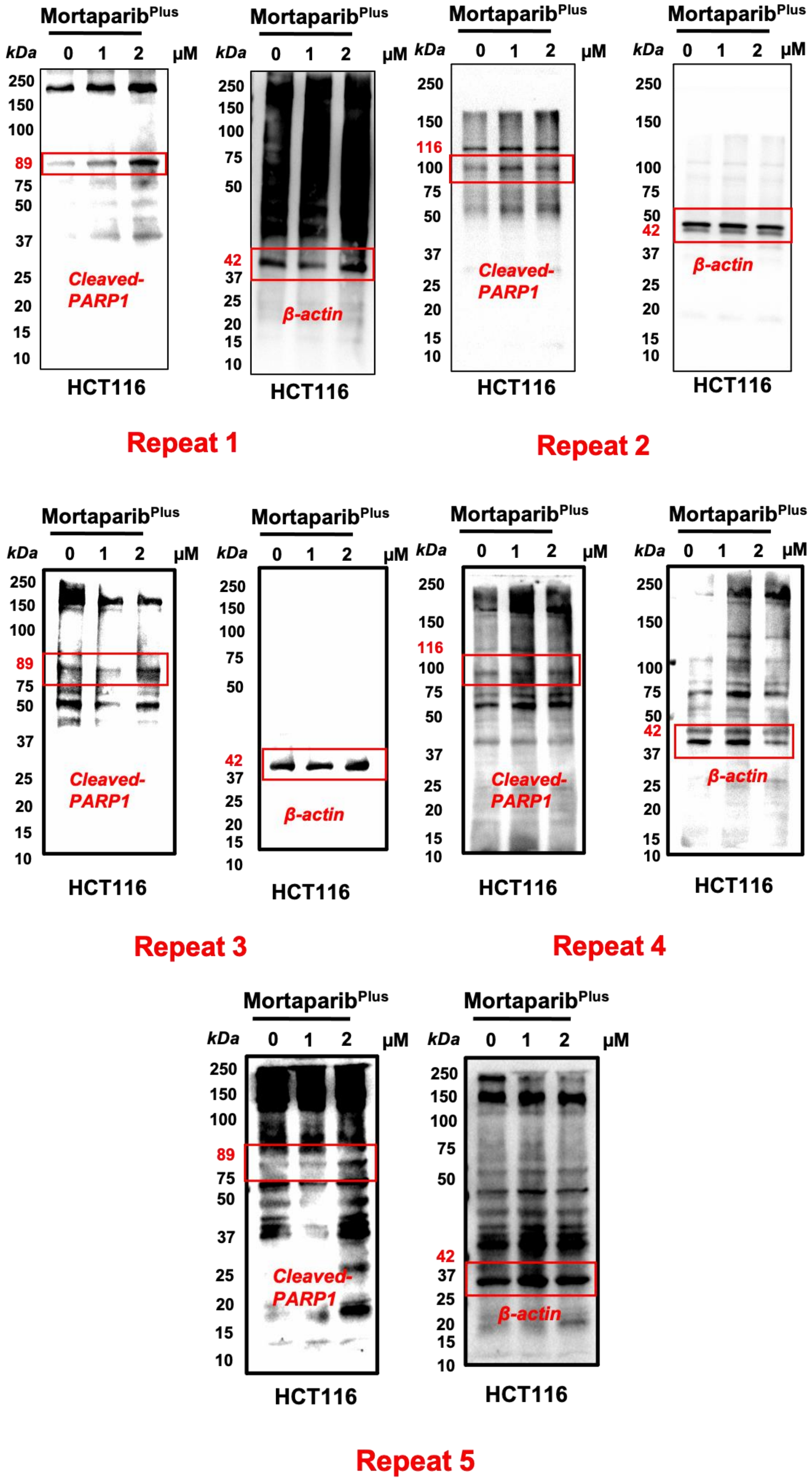
MortaparibPlus
kDa
0 1 2 μM
250
150
100
89
75
50
37
25
20
15
10
Cleaved-PARP1
HCT116
MortaparibPlus
kDa
0 1 2 μM
250
150
100
75
50
42
37
25
20
15
10
β-actin
HCT116
Repeat 1
MortaparibPlus
kDa
0 1 2 μM
250
150
116
100
75
50
37
25
20
15
10
Cleaved-PARP1
HCT116
MortaparibPlus
kDa
0 1 2 μM
250
150
100
75
50
42
37
25
20
15
10
β-actin
HCT116
Repeat 2
MortaparibPlus
kDa
0 1 2 μM
250
150
100
89
75
50
37
25
20
15
10
Cleaved-PARP1
HCT116
MortaparibPlus
kDa
0 1 2 μM
250
150
100
75
50
42
37
25
20
15
10
β-actin
HCT116
MortaparibPlus
kDa
0 1 2 μM
250
150
116
100
75
50
37
25
20
15
10
Cleaved-PARP1
HCT116
MortaparibPlus
kDa
0 1 2 μM
250
150
100
75
50
42
37
25
20
15
10
β-actin
HCT116
Repeat 3
Repeat 4
MortaparibPlus
kDa
0 1 2 μM
250
150
100
89
75
50
37
25
20
15
10
Cleaved-PARP1
HCT116
MortaparibPlus
kDa
0 1 2 μM
250
150
100
75
50
42
37
25
20
15
10
β-actin
HCT116
Repeat 5

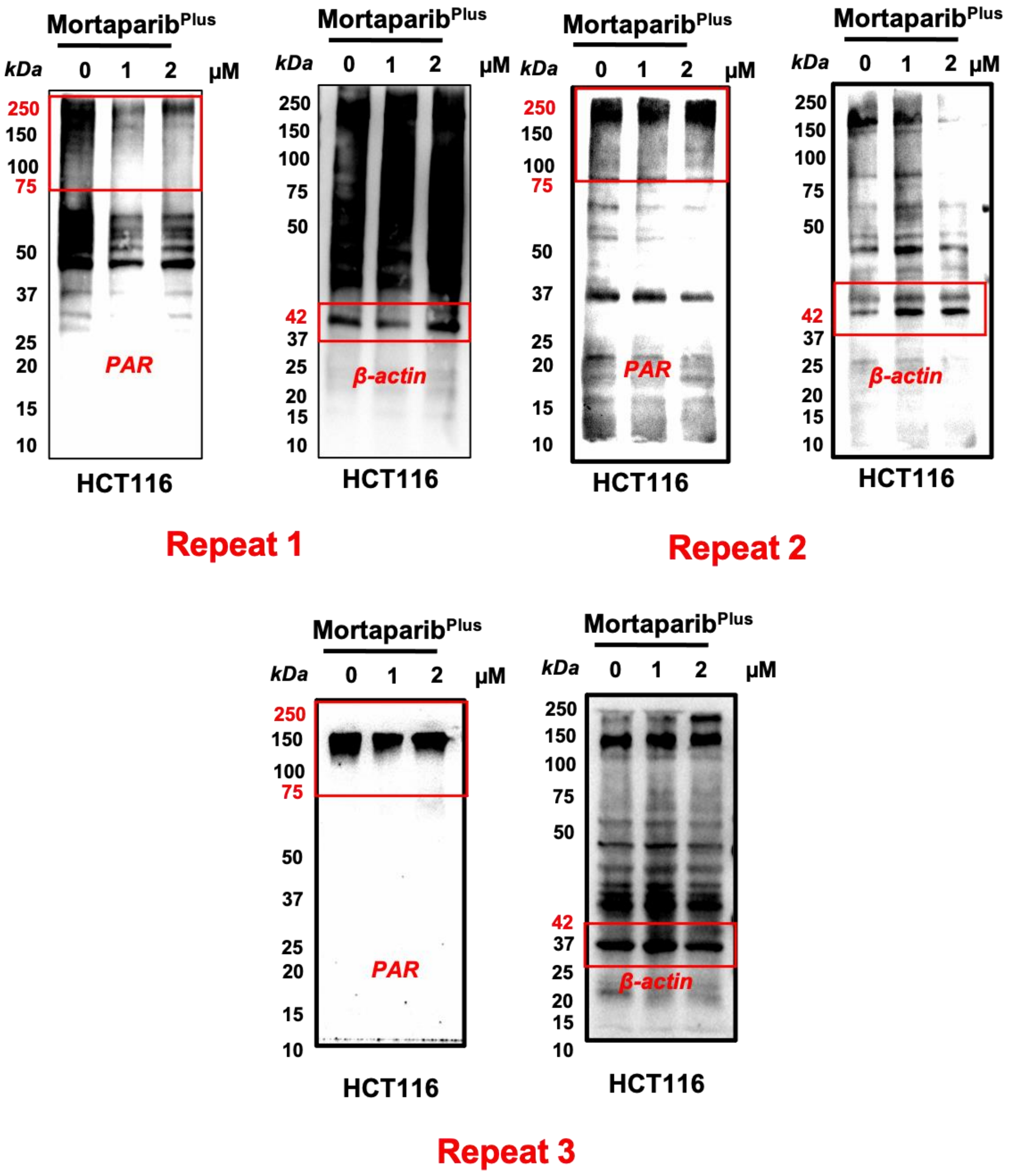

Mortaparib^Plus
kDa 0 1 2 μM
250 150 100 75 50 37 25 20 15 10
PAR
HCT116
Mortaparib^Plus
kDa 0 1 2 μM
250 150 100 75 50 42 37 25 20 15 10
β-actin
HCT116
Repeat 1
Mortaparib^Plus
kDa 0 1 2 μM
250 150 100 75 50 37 25 20 15 10
PAR
HCT116
Mortaparib^Plus
kDa 0 1 2 μM
250 150 100 75 50 42 37 25 20 15 10
β-actin
HCT116
Repeat 2
Mortaparib^Plus
kDa 0 1 2 μM
250 150 100 75 50 37 25 20 15 10
PAR
HCT116
Mortaparib^Plus
kDa 0 1 2 μM
250 150 100 75 50 42 37 25 20 15 10
β-actin
HCT116
Repeat 3
